# Fostering collective climate action and leadership: Insights from a pilot experiment involving mindfulness and compassion

**DOI:** 10.1016/j.isci.2023.106191

**Published:** 2023-02-24

**Authors:** Lena Ramstetter, Silke Rupprecht, Luis Mundaca, Walter Osika, Cecilia U.D. Stenfors, Johannes Klackl, Christine Wamsler

**Affiliations:** 1 Department of Political Science, University of Salzburg, Salzburg, Austria; 2 Centre for Applied Health Sciences, Leuphana University Lüneburg, Lüneburg; Awaris, Germany; 3 International Institute for Industrial Environmental Economics (IIIEE), Lund University, Lund, Sweden; 4 Department of Clinical Neuroscience, Karolinska Institute, Stockholm, Sweden; 5 Department of Psychology, Stockholm University, Stockholm, Sweden; 6 Department of Psychology, University of Salzburg, Salzburg, Austria; 7 Lund University Centre for Sustainability Studies (LUCSUS), Lund University, Lund, Sweden

**Keywords:** Global change, Interdisciplinary application studies, Nature conservation, Psychology

## Abstract

Recent research suggests that mindfulness, compassion, and self-compassion relate to inner transformative qualities/capacities and intermediary factors that can support increased pro-environmental behavior and attitudes across individual, collective, organizational, and system levels. However, current insights focus on the individual level, are restricted to certain sustainability fields, and wider experimental evidence is scarce and contradictory. Our pilot study addresses this gap and tests the aforementioned proposition in the context of an intervention: an EU Climate Leadership Program for high-level decision-makers. The intervention was found to have significant effects on transformative qualities/capacities, intermediary factors, and pro-environmental behaviors and engagement *across all levels*. The picture is, however, more complex for pro-environmental attitudes. With due limitations (e.g., small sample size), this preliminary evidence confirms the feasibility and potential of mindfulness- and compassion-based interventions to foster inner-outer transformation for sustainability and climate action. Aspects that should be taken into account in larger confirmatory trials are discussed.

## Highlights

• Training nourished transformative qualities and capacities
• It reduced climate anxiety and increased environmental self-identity
• It increased pro-environmental behaviors (at individual, collective, and system levels)
• It fostered political agency and climate policy integration
• The pilot has paved the way for larger confirmatory trials

## Graphical abstract

**Figure:**
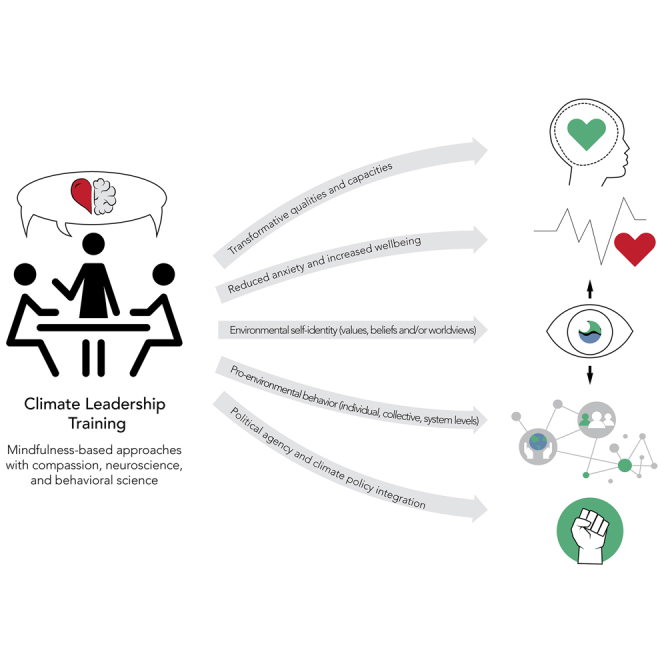

## Teaser

Global change; Interdisciplinary application studies; Psychology

## Introduction

Anthropogenic climate change poses an existential threat to all life on our planet. The conclusion of the latest International Panel on Climate Change (IPCC) report on the consequences of global warming is unequivocal: current climate policies and approaches are putting us on track to reach a 2.8°C temperature rise by the end of this century.^1^^,^^2^ The declared aim of limiting global warming to 1.5°C compared to the pre-industrial era, intended to keep the consequences of climate change at manageable levels, is receding far into the distance.^2^ Instead, we are witnessing a sharp increase in extreme weather events, along with other climate impacts, leading to diverse social crises and an estimated loss of up to 70% of plant and animal species.^1^^,^^3^

It is clear that despite decades of climate negotiations, climate leadership, and action are still far from commensurate with the task at hand. The current situation testifies to the complexity of the challenge we are faced with. Although technological advances and policy solutions exist, change has not manifested to the necessary degree.^4^^,^^5^^,^^6^

One explanation for the failure of current climate approaches is the current focus on external dimensions of climate change. Primarily recognized as a technical challenge, today’s approaches mainly revolve around external socio-economic structures, governance dynamics, and technology improvements.^6^^,^^7^ This technocratic take (or technology optimism) on the climate crisis ignores both its inherent complexity, and the inner mental states (such as climate anxiety, biases, greed, and materialism) that lie at the root of the problem and hamper adequate action.^8^ Global warming is characterized by interdependencies, multiple causations, and complex feedback loops that travel beyond scales and boundaries. When faced with such complex problems, individuals tend to resort to what they know, making attitude and behavior change unlikely, especially if overwhelming inner states such as anxiety or loss of control emerge.^9^^,^^10^^,^^11^^,^^12^^,^^13^^,^^14^ The resulting uncertainty and unpredictability hinders climate action on individual, collective or organizational, and system levels.^7^^,^^8^

More integrative approaches that link the inner and outer dimensions of sustainability are thus urgently needed and have also been highlighted by this year’s IPCC assessment reports on climate change mitigation and adaptation.^4^^,^^5^ Acknowledging the interdependency of inner and outer dimensions, climate change is increasingly understood as a complex human crisis, a crisis of disconnection between oneself, others, and the environment, which is intrinsically linked to other societal crises (e.g., health, poverty).^15^^,^^16^^,^^17^ This narrative of separation underpins a dominant paradigm of unfettered economic growth, deprioritizes care in policymaking, depresses stakeholder collaboration, and manifests in a widespread inability to think and act sustainably. It serves to uphold entrenched power structures and inequalities that limit people’s agency to create change at individual, collective, and system levels.^18^

To adequately address the inherent complexity and unsettling nature of the climate crisis, it is thus critical to better understand the inner dimensions of climate change to foster sustainable climate leadership and action. Research and practice on climate change communication is increasingly touching upon such inner dimensions of change, suggesting, for instance, ways to foster attitudinal change by circumventing the invisible walls of motivated reasoning, and biased perception.^19^ Changing the way we communicate is, however, not sufficient to address the root causes of the problem, which has to do with the human mental states of disconnection and associated social paradigms mentioned above. A growing body of scholarly research thus suggests that a broader cultural shift in mindsets is necessary.^15^^,^^18^^,^^20^^,^^21^^,^^22^ In other words, for socially and ecologically sustainable action to manifest, people’s inner dimensions, defined in the present study as the individual and collective mindsets, values, beliefs, worldviews, and associated inner human capacities, need to be addressed.^23^^,^^24^

Studies that have investigated the inner correlates of pro-environmental attitudes and climate actions suggest that a range of inner human capacities (cognitive, emotional, and relational) is relevant across different levels and is generally conceptualized under the umbrella term transformative (or transformational) qualities/capacities.^17^ Sometimes also called inner sustainable development goals, inner sustainability goals,^17^ or inner development goals,^25^ these capacities are understood to be foundational for achieving the United Nations Sustainable Development Goals.^23^^,^^25^^,^^26^^,^^27^

While the linkages between, and the importance of, inner and outer dimensions of change are increasingly recognized in both scholarly research and IPCC reporting, the nexus between transformative qualities/capacities and sustainable outcomes at individual, collective/organizational, and systems levels remains vastly underexplored.^4^^,^^5^^,^^7^^,^^17^ To better understand whether, and how, certain human qualities can foster transformations toward sustainability, more research is needed: exploring the dynamics of inner change—and how these translate into impactful action across levels—might be key for coming to terms with the climate crisis we are facing.

Existing studies suggest that meditation, and particularly compassion (toward the self, others, and nature) along with mindfulness, might play a crucial role in this context, as they can support conscious (re)connection with self, others, and nature, thereby serving as a foundation for more regenerative, sustainable systems^4^^,^^5^^,^^28^^,^^29^ (for related reviews see^30^^,^^31^^,^^32^^,^^33^^,^^34^). However, empirical evidence of the related, complex internal-external interdependencies is limited.^35^^,^^36^^,^^37^ This is particularly true in the context of professionals, notably their training, and their influence on their organizations and their working environment.^17^

To address this gap, we conducted an experimental pilot study. This 10-week intervention (the EU Climate Leadership Program) targeted high-level decision-makers from different organizations. The program was selected as it was designed to support climate leadership and action by linking inner and outer dimensions and included mindfulness and compassion training (Table 1). The focus was on people with potentially high impact and influence working in the field of sustainability and on organizations with substantial systemic influence. The objective of our pilot study was to provide insights into the potential effects of mindfulness- and compassion-based interventions to foster pro-environmental attitudes and behaviors. By definition, a pilot study aims to provide input to a larger, subsequent confirmatory trial.^38^ Our specific hypotheses are described in the following section.

**Table 1:** Beyond climate leadership program overview

Modules	Problem addressed	Goals of session	Session and home practice examples	Transformative qualities/capacities (clusters) targeted
Module 1: Welcome (Introduction and kick-off)		• Taking ownership of the development journey • Onboarding to the learning platform, app, and collaborative software used • Getting to know and connecting with participants and trainers	• Mindfulness-based practice “Breathing Space” • Group reflection about the personal motivation and understanding of leadership and sustainability	• Awareness • Purpose
Module 2: Connect (Opening the mind)	• Lack of awareness due to perception errors and biases impacts the ability to relate to the climate crisis, nature, and us	• Understanding how awareness is linked to sustainability • Learning how to cultivate openness, curiosity, and awareness, and to create space for habit change • Develop a more relational view of reality by spending time in nature, and by practicing attention and self-regulation	• Mindfulness-based practice: Open, relational awareness (linking inner and outer dimensions) • Dyads and/or small-group reflections: barriers to habit change; how to create space for habit change • Guided nature walk (1): Connecting with one’s senses • Journaling	• Awareness • Insight
Module 3: Open up (Opening the heart)	• Unawareness of emotions and their impact on our (in)activity. • Climate anxiety can negatively affect adequate and sustained engagement in pro-environmental actions across scales	• Learning how to cultivate empathy, awareness, and resilience as a way to explore and regulate the full range of emotions relating to the climate crisis • Experiencing the intelligence of such emotions, and the connection to oneself, others, and nature as a whole	• Mindfulness-based practices: body scan and gratitude meditation (linking inner and outer dimensions) • Guided nature walk (2): nature as a teacher. • Dyads and/or small-group reflections: emotions related to the state of the planet and our shared humanity • Habit change exercises (mindfulness and compassion as a way of living and acting in the world) • Journaling	• Awareness • Connection
Module 4: Care (Increasing care for oneself, others, nature)	• Feeling overwhelmed/lacking confidence to address the climate crisis adequately (informed by a limited view of ourselves in relation to the collective and systems)	• Learning how to cultivate perspective-taking and compassion • Experiencing how the relationship to internal and external events impacts well-being of oneself and the world at large • Compassion as a healing way to relate caringly to oneself and others	• Mindfulness- and compassion-based practices: Compassion for oneself and others • Guided nature walk (3): state of the planet. • Dyads and/or small-group reflection: the nature of self/shared humanity. • Habit change exercises • Journaling	• Connection • Insight
Module 5: Explore (Finding one’s own role)	• Gap in understanding of actions to address climate change • Lack of exploring and understanding the possibilities of becoming active within the work context	• Further exploring one’s own purpose and potential role in effectively addressing climate change • Draft individual and group projects/breakthrough initiatives to be undertaken in the next two weeks	• Mindfulness- and compassion-based practices: compassion for oneself, others, and nature; visioning exercise (purpose) • Guided nature walk (4): Further elaboration of one’s role in addressing the climate crisis • Dyads and/or small-group reflections: reflections on personal calling (agency) • Habit change exercises • Journaling	• Linking all previous clusters
Module 6: Collaborate (Embodying as new way of being, acting, and leading)	• Lack of clarity about which actions may have most impact • Lack of understanding of integrative approaches that link inner and outer transformation	• Developing the collaborative projects/initiatives based on universal, intrinsic values, and purpose • Complexity and systems thinking • Setting up the action lab • Supporting co-creation	• Mindfulness- and compassion-based practices: compassion for oneself, others, the world at large • Guided collaborative ideation process (supporting systems and complexity thinking and co-creation) • Guided nature walk (5): visualizing one’s project coming to life. • Journaling	• Linking previous clusters with agency
Module 7: Action lab (Breakthrough initiatives; developing projects and a community of change)	• Lack of network and community to sustain transformative actions	• Action lab: defining concrete ways in which groups of participants aim to make a difference • Building a network of transformative and compassionate leaders	• Mindfulness-based practice: gratitude • Action lab: prototyping and defining next steps • Journaling	• Linking previous clusters with agency
All modules (Linking individual, collective, and system transformation)	• Sustainability crises • Climate change as an interconnected sustainability crisis • Fragmented approaches for individual, collective, and system transformation	• Linking inner and outer transformation toward sustainability	• Linking and adapting mindfulness practices with other practices for nurturing compassion, habit change, transformational leadership, systems and complexity thinking, and nature-based approaches	• All clusters

Our findings reveal a significant increase in transformative qualities/capacities, intermediary factors (e.g., well-being, reduced climate anxiety), and pro-environmental behaviors at individual and collective/organizational levels (e.g., climate considerations in the workplace). The outcomes show, however, a more complex picture for pro-environmental attitudes. Despite its limitations, notably the small sample size, this pilot study provides some preliminary evidence about the potential of mindfulness- and compassion-based interventions to foster inner-outer transformation for sustainability and climate action. We also discuss some issues that should be taken into account in larger, confirmatory trials.

The remainder of this paper is structured as follows. First, we present the potential effect of transformative qualities/capacities on climate change attitudes and actions, by providing an overview of existing research and theory, and highlighting the present study’s contribution to the field. The following section presents our analyses and results. We conclude with a summary and discussion of the implications of our findings for scholars and policymakers, including methodological issues. Our methodological approach is outlined in the separate STAR methods section.

### Research linking inner and outer transformation

A recent review of research that links inner and outer transformation for sustainability shows that scholars and practitioners alike are increasingly recognizing the importance of inner prerequisites for addressing climate change.^17^ Policy solutions exist, technological advancements abound, but progress remains marginal. Searching for explanations, for some scholars, the research focus has shifted from the macro to the micro level, and from material to internal processes. This has revealed that the predominant focus on large-scale external action has detracted from the internal underpinnings of sustainability.^17^ The latter involves our individual and collective mindsets, values, beliefs, and worldviews, and our associated emotional/cognitive and relational capacities.^17^ (Note: Despite important advances in fields such as environmental psychology, behavioral economics, sustainability science, and education,^39^^,^^40^^,^^41^^,^^42^^,^^43^^,^^44^^,^^45^^,^^46^ existing knowledge is still fragmented, and questions remain as to how different aspects of inner dimensions relate to sustainability outcomes *across* individual, collective, and system levels and vice versa.^17^^,^^27^^,^^47^^,^^48^ This is also reflected in the calls for more integrative policy approaches and better-linking inner and outer transformation for sustainability, including in IPCC’s 2022 assessment reports.^4^^,^^5^)

Research thus increasingly highlights that if we continue to ignore the internal underpinnings of sustainability, climate action is likely to fail.^4^^,^^5^^,^^15^^,^^16^^,^^49^ In liberal democracies in particular, where policy decisions reflect citizens’ desires and needs, external actions need to be underpinned by climate consciousness at the individual level and involve citizens for impactful technological, policy, and societal changes. On the one hand, without strong demand from below, governments lack the legitimacy to enact the far-reaching policies necessary to sustainably combat climate change. On the other hand, without simultaneous change at the individual and collective levels, institutional efforts remain limited in breadth and depth. Even worse, if climate policies cannot draw on the common societal ground, not only are they unlikely to have their intended effects, but they might also lead to opposition and polarization.^50^^,^^51^

Consequently, although citizens' environmental attitudes have received ample scholarly attention,^52^^,^^53^^,^^54^ a wide array of questions remain unanswered. For instance, research to date has treated attitudes toward the environment as mostly static and correlated with given socio-economic structures and demographics (e.g., age, gender, education, income, political affiliation) that prove to be largely stable over time.^55^^,^^56^ In addition, while research into attitude and behavior change has uncovered certain psychological barriers to transformational change, such as biased perception and motivated reasoning, more in-depth studies of the processes of inner-outer transformation are lacking.^17^ What we do know is that efforts to mobilize people to take action toward sustainability simply by providing more information have proven to be difficult and short-lived.^57^ In short, research shows that our current approaches to sustainability (in research, education, and practice) have reached their limits.

These limitations are also a reflection of the dominant epistemological and ontological research paradigms, with the modern worldview of separateness being deeply engrained in our approaches, and related ideas of self, others, and nature (cf. previous section).^17^^,^^58^^,^^59^ Mirroring this separateness, research on sustainability has, so far, vastly neglected the linkages between individual, collective, and system change.

#### Mindfulness and (self-)compassion as potential deep leverage points

In the search for new pathways to foster transformation, research has started to dig deeper, and has moved from so-called shallow leverage points that manipulate external parameters (such as material incentives) to deep leverage points that address the underlying mindsets and associated inner dimensions.^15^ While the latter are considerably harder to reach, shifts at this level have the potential to induce more substantial change, as it is here where system structures and goals are (re)constructed.^60^^,^^61^ (Research on quantum social science goes even further, arguing that individuals are, in themselves, leverage points because of their capacities for building relationships with themselves, each other, nature, and change.^17^^,^^58^^,^^59^)

Although research that explores deep leverage points remains scarce and fragmented, a promising strand of literature is emerging around the effects and correlates of mindfulness, and how it can support the creation of new patterns and relationships (to self, others, nature). Studies that assess linkages between the capacity of “paying attention in a particular way: on purpose, in the present moment, and non-judgmentally,”^62^ and pro-environmentalism, suggest that mindfulness might be a way to target such deep leverage points (for an overview, see ^32^^, cf.^ ^28^).

The foundational human capacity of non-judgmental attentiveness to the present moment is closely intertwined with how people relate to others and the world around them. Mindfulness is, for instance, associated with increased attention regulation,^63^^,^^64^ emotion regulation,^65^^,^^66^ and self-awareness.^67^^,^^68^ It affects the way we relate to others and nature, for example, via values, beliefs, and worldviews.^69^^,^^70^ With regard to the latter, research also suggests that dispositional mindfulness is positively correlated with belief in climate change,^71^ motivation for climate adaptation,^72^ and self-reported pro-environmental behavior.^71^^,^^73^^,^^74^^,^^75^^,^^76^^,^^77^

These preliminary indications of a positive relationship between mindfulness and sustainability have raised expectations. Yet, experimental evidence for understanding the underlying processes and links between (induced) mindfulness and markers of pro-environmentalism in general, and climate change attitudes and action in particular, is scarce. Although experimental studies of the effects of mindfulness interventions on pro-environmentalism are valuable first steps in this direction,^35^^,^^36^^,^^37^^,^^78^ they are limited to the realm of sustainable consumption, and thus capture only a fragment of the pro-environmentalism spectrum related to sustainability and climate action, and they focus generally at the personal level; moreover, they do not include control groups.^78^

In the search for deep leverage points, two concepts that are closely intertwined with mindfulness are relevant: *self-compassion* and *compassion for others*. Research at the interface between climate change, sustainability, and mindfulness is increasingly highlighting the mediating effects of these concepts. By inducing a shift in the perspective of the self,^67^ and by taking an observer perspective,^68^ mindfulness can, for instance, enable a transformation from self-criticism to self-understanding and compassion. The relationship between self-compassion and mindfulness is, however, bidirectional: while mindfulness-based interventions have been shown to enhance self-compassion,^79^ self-compassion and the feeling of interconnectedness can enhance mindfulness.^80^ Relating to one’s own suffering with compassion and understanding can, in turn, transform the connection to others. Depersonalizing experiences of inadequacy, and embedding them in the human condition, generates feelings of empathy and compassion for others.^81^

Hence, mindfulness might also indirectly affect pro-environmentalism via an increase in self-compassion and compassion for others. Related research suggests that increases in compassion are positively linked to pro-environmental intentions,^82^ sustainable decision-making in organizational contexts,^83^ and support for climate mitigation and adaptation measures.^84^

While experimental evidence that mindfulness and (self-)compassion have a causal effect on facets of sustainability remains shallow and is questionable in a context of complex systems (e.g., emerging epistemological, ontological, and ethical understanding that underlies research on inner-outer transformation questions the focus of specific outcomes and the value of cause-effect reductionism when working with complex systems^17^), correlational and exploratory intervention studies provide a reason for optimism. In a review of the literature on the inner determinants of climate change attitudes and actions, Wamsler et al.^17^ developed a model of inner-outer transformation. They describe *five clusters of transformative qualities/capacities* that underpin sustainability: awareness, connection, insight, purpose, and agency. (Accordingly, the following definitions from Wamsler et al.^17^ have been adopted in the present paper. *Awareness*: "The ability to meet situations, people, others and one’s own thoughts and feelings with openness, presence and acceptance". *Connection*: "The ability and desire to see and meet oneself, others, and the world with care, humility and integrity, from a place of empathy and compassion". *Insight*: "The ability to see, understand, and bring in more perspectives for a broader, relational understanding of oneself, others and the whole". *Purpose*: "The ability to navigate oneself through the world, based on insights into what is important (intrinsic, universal values)". *Agency*: "The ability to see and understand broader and deeper patterns and our own role in the world in this regard, and to have the intention, optimism and courage to act on it".) These five clusters of transformative qualities/capacities, together with other intermediary factors (e.g., reduced climate anxiety, increased well-being), can influence our values, beliefs, and worldviews, which, in turn, determine how we relate to self, others, nature, and change^17^ (for an illustration of the inner-outer transformation model see Figure S9 in supplemental information). Upon closer inspection, each cluster and the associated intermediary factors relate to mindfulness and (self-)compassion, while compassion and self-compassion can be seen as foundational capacities or traits that influence our relational being, thinking, and acting.^17^ At the same time, research with both clinical and non-clinical populations consistently shows that capacities such as mindfulness and compassion can be moderately improved by training.^85^

Drawing on these theoretical insights, we derive three sets of hypotheses about the intervention outcomes within and between the groups:

##### Transformative qualities and capacities

As mindfulness and (self-)compassion are potentially linked to all five clusters of transformative qualities/capacities, and related intermediary factors, we hypothesize that participants taking part in the intervention will report a significant increase in transformative skills, i.e., mindfulness (Hypothesis 1a), self-compassion (Hypothesis 1b), compassion with others (Hypothesis 1c), connectedness with nature (Hypothesis 1d). In addition, based on the described model (see above), we expect an increase in well-being and a reduction in climate anxiety (Hypothesis 1e), with the latter being increasingly seen as an obstacle to adequate and sustained climate action.^86^^,^^87^^,^^88^^,^^89^^,^^90^^,^^91^

##### Pro-environmental attitudes

We expect participants in the intervention group to be significantly more likely to report an increase in their pro-environmental attitudes, particularly their environmental self-identity (Hypothesis 2a) and their belief in climate change (Hypothesis 2b). We also expect an increase in their willingness to “pay” for climate action at both personal and societal levels (Hypothesis 2c). As theoretical expectations regarding environmental concerns diverge,^14^ we do not propose a hypothesis for this sub-dimension.

##### Pro-environmental behaviors and engagement across levels

We expect that compared to participants in the control group, participants in the intervention group will report increased pro-environmental behaviors, understood as behaviors that protect or avoid harm to the environment (Hypothesis 3). Finally, we expect spill-over effects on people’s engagement at the organizational/system level that support climate policy integration/mainstreaming (Hypothesis 4).

## Results

This section outlines the results of our analyses and presents them in the light of our hypotheses and associated research in the field, which we presented in the previous section. In line with our theoretical underpinnings, we begin with our findings related to the inner antecedents of change (transformative qualities/capacities), then move to the outer manifestations (actions). Table 2 summarizes within- and between-groups statistical results.

**Table 2:** Within-group and between-group statistical differences for dependent variables and effect sizes

	Intervention Group (N = 65)								Control Group (N = 29)								Between-group difference: intervention effect			
	Pre			Post		Within-group difference			Pre			Post		Within-group difference						
	N	Mean	SD	Mean	SD	p value (1)	Sig	Cohen’s d (2)	N	Mean	SD	Mean	SD	p value (1)	Sig	Cohen’s d (2)	Estimate	SE	p value (3)	Sig
**Environmental attitudes**																				

Environmental attitudes: Environmental concern	128	17.17	2.28	16.89	2.71	0.2963		−0.11	76	16.13	2.73	15.39	2.76	0.0616	∗	−0.27	0.4670	0.4436	0.2950	
Environmental attitudes: Climate anxiety	130	3.82	1.06	3.37	1.10	0.0018	∗∗∗	−0.41	76	3.55	1.29	3.34	1.05	0.1860		−0.18	−0.2356	0.2154	0.2766	
Environmental attitudes: Beliefs about fighting climate change	127	12.08	2.22	11.89	2.15	0.3213		−0.10	58	11.14	2.07	11.00	2.02	0.6408		−0.07	−0.0717	0.3661	0.8451	
Environmental attitudes: Environmental self-identity	130	4.25	0.69	4.32	0.71	0.2545		0.11	58	4.34	0.67	4.17	0.60	0.0961	∗	−0.27	0.2493	0.1204	0.0412	∗∗
Environmental attitudes: Willingness to pay, personal	129	4.00	0.87	4.08	0.62	0.4392		0.10	58	3.79	0.62	3.76	0.69	0.7869		−0.05	0.1126	0.1718	0.5137	
Environmental attitudes: Willingness to pay, societal	130	4.60	0.49	4.45	0.88	0.1327		−0.20	58	4.38	0.78	4.14	1.06	0.1474		−0.25	0.0875	0.1859	0.6389	

**Environmental behavior**																				

Environmental behavior: Sum	124	55.00	11.21	56.68	10.81	0.0486	∗∗	0.15	58	45.72	10.19	46.76	9.56	0.2620		0.10	0.6604	13,584	0.6281	
Environmental behavior: Adapt	129	2.62	1.08	2.88	0.98	0.0615	∗	0.26	58	2.17	0.89	2.14	0.95	0.8455		−0.04	0.3001	0.2387	0.2118	
Environmental behavior: Agency	129	19.45	6.44	20.20	6.32	0.0480	∗∗	0.14	58	13.76	6.53	13.41	6.02	0.5662		−0.05	12,198	0.7588	0.1114	
Environmental behavior: Transport	128	15.29	3.47	15.78	3.33	0.2230		0.13	58	14.17	4.22	15.17	3.67	0.1378		0.25	−0.5714	0.6779	0.4015	
Environmental behavior: Food	128	10.05	3.06	10.25	3.14	0.2908		0.08	58	8.69	2.99	8.76	2.64	0.8132		0.02	0.1850	0.4027	0.6470	
Environmental behavior: Waste	127	7.62	1.31	7.75	1.21	0.6192		0.06	58	6.93	1.58	7.28	1.49	0.1861		0.22	−0.2642	0.2932	0.3700	

**Organizational mainstreaming**																				

Organizational mainstreaming: Do you stand up	130	4.00	0.81	3.85	0.87	0.0675	∗	−0.18	58	3.17	0.93	3.21	1.08	0.8390		0.03	−0.1883	0.1670	0.2623	
Organizational mainstreaming: Integration at work	124	29.52	8.04	31.46	9.27	0.0414	∗∗	0.20	58	27.79	10.90	23.83	9.35	0.0081	∗∗∗	−0.38	57,621	15,675	0.0004	∗∗∗
Organizational mainstreaming: Intention next 12 months	130	4.08	1.07	4.23	1.06	0.2139		0.14	58	2.66	*1.54*	3.00	*1.39*	*0.1941*		*0.23*	−0.1910	0.2518	0.4502	

**Transformative** qualities/capacities																				

Mindfulness: Sum	129	27.18	3.99	29.47	4.53	0.0003	∗∗∗	0.53	0	NaN	*NA*	NaN	*NA*	*NA*	*NA*	*NA*	*NA*	*NA*	*NA*	*NA*
Mindfulness: Non-judgment	130	6.38	2.87	5.38	2.45	0.0007	∗∗∗	−0.37	0	NaN	*NA*	NaN	*NA*	*NA*	*NA*	*NA*	*NA*	*NA*	*NA*	*NA*
Mindfulness: Non-reacting	129	6.49	1.67	6.73	1.59	0.2682		0.14	0	NaN	*NA*	NaN	*NA*	*NA*	*NA*	*NA*	*NA*	*NA*	*NA*	*NA*
Mindfulness: Acting with awareness	130	6.28	1.80	6.58	1.76	0.1589		0.17	0	NaN	*NA*	NaN	*NA*	*NA*	*NA*	*NA*	*NA*	*NA*	*NA*	*NA*
Mindfulness: Observe	130	3.52	1.13	3.45	1.16	0.6000		−0.07	0	NaN	NA	NaN	NA	NA	NA	NA	*NA*	*NA*	*NA*	*NA*
Subjective well-being: Sum	129	20.60	5.49	21.59	5.52	0.0336	∗∗	0.18	58	16.07	6.88	17.17	6.95	0.2402		0.16	−0.1347	0.9054	0.8821	
Subjective well-being: Social well-being	130	5.89	2.41	6.42	2.43	0.0368	∗∗	0.22	58	4.52	2.69	4.90	2.40	0.3702		0.15	−0.2483	0.3014	0.4122	
Subjective well-being: Emotional well-being	130	7.32	1.79	7.52	1.77	0.2156		0.11	58	5.45	2.29	5.90	2.44	0.1136		0.19	−0.0259	0.3840	0.9465	
Subjective well-being: Psychological well-being	129	7.38	2.07	7.64	2.07	0.1937		0.12	58	6.10	*2.79*	6.38	*2.69*	*0.4822*		*0.10*	0.1438	0.4604	0.7555	
Connectedness with nature	130	4.18	1.96	5.20	1.44	0.0002	∗∗∗	0.58	29	NaN	*NA*	4.14	1.16	*NA*	*NA*	*NA*	*NA*	*NA*	*NA*	*NA*
Self-Compassion: Sum	129	21.38	3.96	22.27	3.55	0.0168	∗∗	0.25	0	NaN	*NA*	NaN	NA	*NA*	*NA*	*NA*	*NA*	*NA*	*NA*	*NA*
Compassion for others: Sum	129	19.40	2.89	19.61	2.80	0.5516		0.07	0	NaN	*NA*	NaN	*NA*	*NA*	*NA*	*NA*	*NA*	*NA*	*NA*	*NA*
Compassion for others: Engage	130	7.38	1.49	7.32	1.43	0.7451		−0.04	0	NaN	*NA*	NaN	*NA*	*NA*	*NA*	*NA*	*NA*	*NA*	*NA*	*NA*
Compassion for others: Act	129	12.02	1.83	12.27	1.80	0.2886		0.14	0	NaN	*NA*	NaN	*NA*	*NA*	*NA*	*NA*	*NA*	*NA*	*NA*	*NA*

(1) based on paired t-test

(2) based on paired Cohen’s d.

(3) based on linear regression models.

### Effects on transformative qualities and capacities

**Figure 1:**
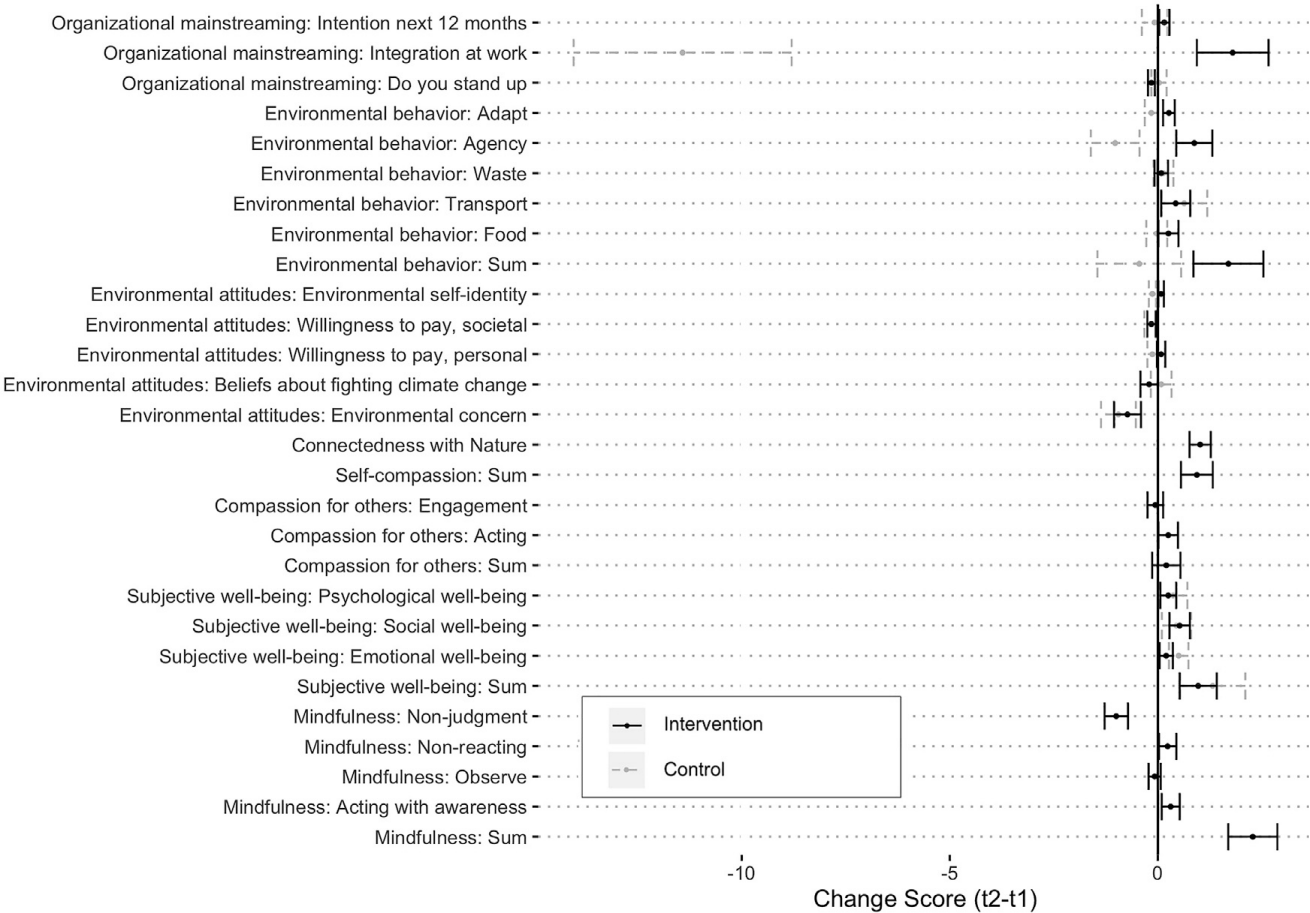
Distribution of change scores in dependent and mediating variables with standard errors Note: Mean change scores (t2-t1) by variable. Error bars represent standard errors.

In line with our hypotheses, our results suggest a significant increase in three transformative qualities in the intervention group (Hypotheses 1a–c). First, overall *mindfulness* increased significantly in the intervention group (*d* = 0.53, p < 0 .001; for a visual overview of pre-post changes, see Figure 1). Second, a strong and significant increase was found for *connectedness with nature* (*d* = 0.58, p < 0 .0001) after training. Third, *self-compassion* slightly increased after the intervention (*d* = 0.25, p = 0.017). However, as none of these variables were assessed in the control group post-intervention, it is difficult to make any causal inferences regarding its effects (i.e., between-group changes), and we must cautiously rely on within-group tests. That said, and contrary to other results reported in the literature, our findings suggest that the intervention did not affect either *compassion for others* (*d* = 0.07, p = 0.552), or its sub-dimensions.

Upon closer inspection, our results provide a more nuanced view of the effects of the intervention on mindfulness: in addition to the overall increase post-intervention, we detected a significant decrease in the *non-judgmental* sub-dimension (*d* = −0.37, p < 0 .001). Two explanations are possible. First, the intervention may have increased the participants’ awareness of their tendency to judge sensations, experiences, and actions more than it increased their tendency to judge. In other words, the decrease in the non-judgmental sub-dimension might be the result of an increase in self-reflection. The intervention thus may have increased participants’ awareness of their tendency to judge sensations, experiences, and actions. This seems the most plausible explanation, as the issue of cognitive bias was an important and integral part of the intervention (see Table 1). At the same time, this finding might also reflect the non-monotonicity of mindfulness practice, suggesting a decrease in non-judgment once a turning point has been passed.^92^

The substantial increase in *connectedness with nature* (+24.3%) in the intervention group confirms Hypothesis 1d and corroborates extant experimental research on the mindfulness-nature nexus.^93^ It supports an optimistic outlook on the efficiency of mindfulness-based programs to foster sustainability across different levels.^15^^,^^94^ In the same vein, the small but significant increase in *self-compassion* (+4.12%) supports extant findings on the effects of mindfulness-based interventions.^95^ No effect was detected for *compassion with others*. However, here, pre-training scores were very high, and a ceiling effect seems to be the most likely explanation (Scale Mean: 19.25, SD = 2.89, Scale Range 0–25). Related aspects are discussed in more detail in the Discussion and conclusions section.

In line with our hypotheses, our results also reveal some interesting changes regarding intermediary factors (Hypothesis 1e). Notably, there was a significant increase in *overall well-being* (*d* = 0.18, p = 0.03) in the intervention group, which was mostly driven by an increase in social well-being (*d* = 0.22, p = 0.04). However, the control group also reported a similar (but non-significant) increase in well-being. Related aspects are discussed in more detail in the next section.

With regard to *climate anxiety* (Hypothesis 1e), our analysis found a significant reduction (*d* = −0.41, p = 0.0018) in the intervention group. Although a similar trend was identified for participants in the control group, no significant between-group differences were detected (*b* = .−236, p = 0.277). This finding is consistent with the literature on the effects of mindfulness on emotion regulation: as the individual becomes aware of the transient nature of emotions, he or she is increasingly able to distance themselves from feelings and sensations that arise, thereby reducing emotional reactivity.^96^ In the long term, this shift in self-regulation may reduce feelings of anxiety, and thereby create sustainable action pathways. The ability to “hold[ing] one’s painful thoughts and feelings in balanced awareness rather than over-identifying with them”^97^ opens up space that was previously occupied by interpretations and anticipations, reducing habitual responding^98^ and widening the thought-action repertoire of individuals.^99^

### Effects on pro-environmental attitudes

Turning to pro-environmental attitudes, our results are mixed. On the one hand, they suggest that participants in the intervention group experienced a significant increase in *environmental self-identity* compared to the control group (*b* = 0.249, p = 0.04), lending support to Hypothesis 2a, and indicating important changes in people’s beliefs/worldviews (cf. Section on Research linking inner and outer transformation). On the other hand, participants in the intervention did not report a significant increase in their *willingness to pay* for climate action either at the individual level (*d* = 0.10, p = 0.439) or at the societal level (*d* = −0.20, p = 0.133; Hypothesis 2c); particularly if the traditional 5% significance threshold is considered. However, since the course supported an integrated (rather than current one-sided) understanding of climate action that links individual, collective, and systems approaches that question the value of isolated financial and regulatory measures,^cf.^ ^17^ this outcome was ultimately interpreted as positive. In addition, *beliefs in climate change* (*d* = −0.10, p = 0.321; Hypothesis 2b) did not change significantly after the intervention, which is likely to be related to ceiling effects in the target group (see also Discussion and conclusions section).

Finally, although we did not establish a hypothesis regarding the environmental concern, our findings warrant attention. While we found no change in the intervention group (*d* = .−11, p = 0.296), there was a small, but significant decrease in the control group (*d* = −0.27, p = 0.062), and no significant difference between groups (*b* = 0.467, p = 0.295). This reduced concern for the environment may be the result of competing claims for attention. At the time the pilot was run, the Covid-19 pandemic gripped the world, and worries about the (rather distant) effects of climate change might have paled in comparison.^100^

### Effects on pro-environmental behaviors and engagement across levels

Moving from attitudes to behaviors and actions, our results show that *pro-environmental behavior* increased significantly among individuals in the intervention group (Hypothesis 3), compared to pre-intervention levels (*d* = 0.15, p = 0.049), while between-group differences remained insignificant (*b* = 0.660, p = 0.628). Disaggregating pro-environmental behavior suggests that the increase in the intervention group was driven by a gain in adaptation behavior (*d* = 0.26, p = 0.061), and environmental/political agency (*d* = 0.14, p = 0.048). *Adaptation behavior* refers to the degree to which people take measures to prepare for potential climate impacts, while environmental/political *agency* refers to actions such as voting for environmental parties or signing petitions. Apart from *transport* behavior (*d* = 0.13, p = 0.223), which may be considered significant if levels for pilot studies are considered, *food* (*d* = 0.08, p = 0.291), and *waste* (*d* = 0.06, p = 0.619) behaviors did not change significantly. The lack of a significant effect might, in this context, point to both a ceiling effect in the intervention group and to a potential null effect due to Covid-related restrictions (for example, on transportation).

Shifting to the *organizational sphere*, our analysis confirmed that participation in the intervention led to a significant increase in organizational mainstreaming (Hypothesis 4). Individuals in the intervention group reported a small but significant increase in the integration of climate considerations in the workplace (*d* = 0.20, p = 0.008), while the control group reported a significant decline (*d* = .−38, p = 0.008), indicating that the intervention had a significant positive effect (*b* = 0.5.76, p <0 .0001). This change in the intervention group is especially important, as interventions at the organizational level were particularly hard to implement during the COVID-19 pandemic, due to related restrictions.

**Figure 2:**
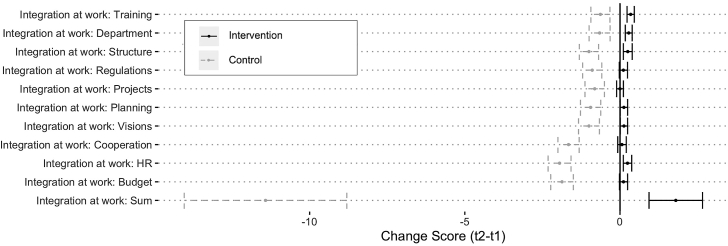
Integration in organizational sphere: distribution of change scores with standard errors *Note:* Mean change scores (t2-t1) by variable. Error bars represent standard errors.

Disaggregating the overall score for climate considerations (i.e., the extent to which climate issues are considered/integrated into the current work) by dimension (see Figure 2) revealed that the identified increase in organizational mainstreaming was driven by a change in several areas of climate policy integration: human resource allocation (*b* = 1.48, p < 0 .0001), budget allocation (*b* = 1.42, p < 0 .0001), increased consideration of climate change issues in cooperation with external stakeholders (*b* = 0.96, p = 0.001), and changes in internal working structures, such as groups or staff mandated to integrate the issue across sectors (*b* = 0.60, p = 0.029).

In addition, we found a significant decrease in the *willingness to stand up* for climate mitigation action at work in the intervention group (*d* = −0.18 p = 0.067), although there was no significant between-group effect (*b* = −0.188, p = 0.262). With regard to the *intention* to increase the integration of climate issues in the workplace over the next 12 months, potential effects were detected (*d* = 0.14, p = 0.214) if significance levels for pilot studies are considered.

Finally, the pilot study identified some exploratory outcomes which could be followed up in future larger trials. Two aspects seem to be particularly relevant in this context. First, our results identified linkages between *compassion* and *pro-environmental behavior*, indicating that an increase in compassion toward others is significantly related to acting in ways that benefit the environment (*r* = 0.29, p < 0 .05). Second, changes in mindfulness were positively correlated with an increase in environmental/political agency (*r* = 0.38, p < 0 .01), and the personal willingness to pay for climate action (*r* = 0.25, p < 0 .05). Both of these findings warrant closer attention in future trials. For an overview of other correlational analyses, see supplemental information, Table S6, and Figure S10.

## Discussion and conclusions

Our experimental pilot study investigated the effects of a 10-week Climate Leadership Program through both between-group and within-group comparisons. It responds to recent calls for more attention to inner-outer transformation and meditation in sustainability and climate policy,^4^^,^^5^ and it builds on insights from previous research which suggest that mindfulness and compassion may be positively related to a range of pro-environmental attitudes and behaviors in the private and collective spheres. The pilot study allowed us to address some outstanding gaps in current research. Most existing studies are observational, and only a few are experimental.^35^^,^^37^^,^^78^^,^^101^ Moreover, these earlier studies have some limitations, as they only captured particular sub-dimensions of sustainability such as consumption behavior, and/or drew on student samples. Consequently, there is a lack of empirical evidence regarding the complex linkages between inner and outer transformation for sustainability. This is particularly true in the context of professionals, their training, and their influence on their organizations/working environment and associated systems.^17^ Our study addresses this gap, as the Climate Leadership Program was targeted at high-level decision-makers from different European organizations.

Despite the intrinsic challenges of pilot studies,^102^^,^^103^ the outcomes of our research extend current insights into the interdependency of inner and outer transformation across different levels and provide a basis for larger confirmatory trials. With due limitations, which are discussed in more detail below, the key findings are 5-fold.

First, participants in the intervention group reported a significant increase in three capacities intrinsically linked with all five clusters of transformative qualities/capacities: mindfulness, self-compassion, and nature-connectedness. (As our exploratory correlational findings also indicate that changes in mindfulness and (self-)compassion might be mutually constitutive, the inner dimensions of sustainability warrant further attention. For instance, our results indicate that changes in self-compassion correlate positively with changes in compassion toward others [see Table S4].)

Second, participants in the intervention group also reported a significant reduction in climate anxiety and increased well-being after the intervention.

Third, by comparison with the control group, participants in the intervention group reported a significant increase in environmental self-identity, which, together with other aspects (e.g., nature connectedness) indicates a change in their values, beliefs, and/or worldviews. (Our outcomes thus point toward the potential influence of mindfulness and compassion to support a paradigm shift regarding how we see our self, others, work, and nature; it can influence how we see current social, political and institutional systems, democracy, and one’s role in them. It involves questioning current social norms that are incompatible with the climate crisis.)

Fourth, participants in the intervention group reported a significant increase in pro-environmental behaviors compared with pre-intervention levels.

Fifth, pro-environmental effects were not limited to the individual level (e.g., personally implementing adaptation measures), but also extended to the collective and organizational spheres. The latter manifested as an increased political agency (e.g., voting for environmental parties, signing petitions), and the integration of sustainability concerns into the working context (climate policy integration, e.g., through changes in budget allocation, human resource allocation, internal working structures, and stakeholder relationships) compared to both pre-intervention levels, and the control group.

While these findings are encouraging and support the inner-outer transformation model (presented in the section on Research linking inner and outer transformation), they should be confirmed in a larger trial. In addition, a number of methodological and design elements need to be taken into account in future research and associated policy interventions.

First, the small sample size and chosen significance (alpha) level underline the importance of Type 1 and Type 2 errors that analysts can encounter, particularly at this stage. At the risk of stating the obvious, like any other pilot study, our sample is rather small, which reduces its statistical power. For example, our analysis failed to detect intervention effects for several variables, the exceptions being an increase in environmental self-identity and greater integration of sustainability into different organizational dimensions (climate policy integration) if the traditional 5% threshold is used. Four explanations are possible. First, to avoid attrition, certain items that aimed to probe transformative qualities/capacities were excluded from the survey administered to participants in the control group. As expected, this severely limited between-group comparisons, and inevitably forced us to rely more on within-group tests. While we cannot exclude the possibility that certain increases were due to a factor unrelated to the intervention, our initial findings are encouraging and form a good basis for larger trials.

Second, it is important to acknowledge that our pilot study was potentially subject to sampling bias: the people who were recruited were already highly engaged in climate-related action. Notably, bias could relate, on the one hand, to the objectives of the intervention and the target group, who are likely to have had an interest in linking their expertise with inner or outer aspects of sustainability. On the other hand, participants were mainly recruited through a training company and sustainability-related professional groups, rather than a research institute. Evidence of related bias can be seen in a follow-up course that the training company ran with a less experienced sample (and no control group) after the pilot ended.^104^

Third, significant baseline differences in pro-environmental attitudes and behaviors between the intervention and control groups limit opportunities to identify significant change (see supplemental information, Table S2). As many of the participants in the intervention group ranked in the highest quartile on several sustainability scales, sensitivity to upward change was severely compromised. As control group participants reported significantly lower baseline measures, comparatively small changes were likely to outweigh any improvements in the intervention group, leading to the lack of a significant effect.

Fourth, in contrast to other studies, we applied a mixed control condition consisting of both a waitlist and an active control. As the majority of individuals in the control group participated in an extended mindfulness program that could be expected to yield similar results,^78^ the bar for an intervention effect was set high (see supplemental material S3). Since the main focus of our pilot study was to investigate interlinkages between the inner and outer dimensions of transformation, this particular decision was a conscious choice. However, it had negative side effects regarding the identification of changes in particular transformative qualities/capacities. In this context, any within-group changes become even more relevant.

Turning from between-to within-group changes, we identified a number of significant effects, particularly with respect to transformative qualities/capacities. Furthermore, we observed relatively small, but consistent changes in individual pro-environmental behaviors in the intervention group. At the same time, and potentially driven by the methodological aspects indicated above (sample size and, especially, ceiling effects), the pilot did not detect an effect of the intervention on beliefs about climate change. Overall, respondents in the intervention group reported a high level of climate awareness and pro-environmentalism at baseline (see Table 2), leaving relatively little room for improvement and any corresponding statistically significant effects post-intervention.

While the positive effects on engagement in the organizational sphere were significant, the study also raised related questions, as, at the same time, participants reported a significant decrease in their willingness to stand up for climate issues at work. The extent to which climate issues are considered in different organizational spheres deserves attention in future confirmatory trials. Our seemingly contradictory outcomes could be related to baseline sample characteristics: participants in the intervention group reported rather high levels of organizational mainstreaming already pre-intervention (mean(t1) = 4.08; see supplemental information, Table S2). Over a small range (0–5), the sensitivity to an increase is likely to be bound by a ceiling effect. In contrast, variables related to climate considerations at work were measured on a 50-point scale, which is considerably more sensitive to change, especially given the more central mean at baseline (mean(t1) = 29.52). At the same time, it is likely that participants perceived their possibilities to stand up for climate issues at work to be limited because during the training course, most were working from home due to COVID-19 restrictions. These circumstances could explain the decline in the control group and underlie the intervention’s positive effects regarding organizational mainstreaming in the intervention group.

The conditions in which the intervention took place could also help to put some other limited effects into perspective. First, as already mentioned, it was run at the height of the COVID-19 pandemic. Second, its short duration could have limited the emergence of certain complex interlinkages between the inner and outer transformation. It may be unrealistic to expect deeply engrained environmental attitudes and behaviors to change significantly during a 10-week period. Extending the time frame, and using follow-up measures at different post-intervention time points, should be considered in future trials. The latter observation echoes studies which suggest that changes in transformative qualities/capacities might have only gradual effects on sustainable behaviors and that highlight that inner and outer transformation is a long-term learning process.^37^ As in any experiment, it is crucial to ensure that related response rates are statistically significant for reliable *ex ante* vs. *ex-post* comparisons.

In addition to the aforementioned limitations, several other aspects should be considered in confirmatory trials. Future studies should naturally aim for a larger, more powerful sample, as this would facilitate the detection of, for example, confounding effects, along with attitudinal and behavioral change. Moreover, as our sample consisted mostly of highly educated individuals in leadership positions, the external validity of our findings is questionable—although we make no claims in this respect. Whether, and how, a more diverse sample would react to the same intervention needs to be considered in future trials. Furthermore, some of our measures should be treated with caution. Sustainable behaviors, on both individual and organizational levels, were self-assessed. Although these measures are widely used in research, and their psychometric properties are demonstrated, they remain susceptible to social desirability bias, misperception, and misinterpretation.^105^ To circumvent these pitfalls, future confirmatory trials could combine qualitative and quantitative approaches, and evaluate revealed rather than stated preferences.

Finally, links between our study and the existing literature also offer some important lessons and insights regarding the content of the intervention and how to improve similar courses in the future to mutually support inner and outer transformation. First, it seems advisable to link inner and outer dimensions of sustainability more closely from the beginning, so that both become natural integral conditions for transformation and engagement at individual, collective, organizational, and system levels (rather than taking a segregated, step-by-step approach).^106^ Second, this cannot be achieved by simply adjusting the content of certain modules. Instead, it requires the integration of practical project work from the outset, while at the same time offering participants a safe space to nourish transformative qualities and capacities, and to critically reflect on their worldviews, beliefs, and values.^23^^,^^107^^,^^108^ Third, the creation of communities of practice is a crucial supplementary continuation that would ensure sustained engagement and change across different levels.^23^^,^^109^^,^^110^^,^^111^ Fourth, providing participants with more input regarding the different facets and aspects of organizational change (e.g., operational processes and other climate policy integration/mainstreaming measures) could help them to translate and integrate inner dimensions into concrete outer measures.^112^^,^^113^ Finally, it is important to reconsider and strengthen the relevance of certain training methods. This requires further quantitative and qualitative studies, as it is possible that we have not yet found the best balance for adapting mindfulness and compassion-based approaches to addressing sustainability challenges, and the underlying worldviews, beliefs, and paradigms.^33^ In this context, the integration of more nature-based approaches seems a promising avenue.^114^

Such lessons are crucial, as many organizations and leaders in both the public and private sectors are increasingly looking for ways to improve their sustainability and reduce their climate impact. At the same time, mindfulness and compassion scholars and trainers are increasingly interested in offering ways to move beyond self-care and individual well-being approaches and to define pathways for responding to the world with intentional action and connecting with others in a changing environment.^115^

Training programs such as the one we investigated in our pilot study thus provide a unique opportunity to improve current knowledge and approaches. Policy support for investing in the development and evaluation of educational programs that support inner and outer transformation for different target groups (leaders, citizens, children) and simultaneously foster climate policy integration is a missing element in improving current approaches.^17^ Developing train-the-trainer programs could in this context help to upscale efforts, and reach the increasing number of people who are suffering from climate anxiety and/or want to find ways for meaningful engagement.^87^ (Note that the outcomes and learnings of this study also led to the development of the Mindfulness-Based Sustainable Transformation [MBST] course and associated train-the-trainer programs. The term MBST and the MBST concept and approach were thought up and defined by the lead contact [CW] on the basis of her research. MBST builds on Mindfulness-Based Stress Reduction [MBSR] and Mindfulness-Based Cognitive Therapy [MBCT] approaches by adapting and linking them to other methods and strategies for enabling integrative individual, collective, and system transformation.)

In sum, given the framework conditions and methodological aspects described previously, our findings suggest that we can be cautiously optimistic about designing a large confirmatory trial: even under challenging circumstances, individuals who participated in the intervention reported significant increases in transformative qualities/capacities, individual pro-environmental behaviors, and engagement at collective and organizational levels. Although seemingly distant from behavior and systems approaches, nourishing transformative qualities (or so-called inner development goals) might be a promising, complementary way to effect change in how we relate to ourselves, others, and nature, and, ultimately, support international, national, and locally set climate and sustainability goals. Based on the points outlined above, we conclude with a call for further research and policy development for advancing integrative approaches that link the inner and outer dimensions of sustainability. This involves lines of research and policy support to investigate in more depth how inner capacities and leverage points relate to the United Nations 17 Sustainable Development Goals, the design, adaptation, contextualization and measurement of related methods, and strategies that link individual, collective, and system change and direct limited resources to their exploration, systematic consideration, and integration in education, organizations, professional groups, and society at large.

## STAR★Methods

### Resource availability

#### Lead contact

Further information and requests for resources should be directed to and will be fulfilled by the lead contact, Christine Wamsler (christine.wamsler@lucsus.lu.se).

#### Materials availability

• This study did not generate new unique materials.
• The Climate Leadership course assessed in this study will be made available on request, but we may require a payment and/or a completed materials transfer agreement if there is potential for commercial application.

### Method details

To address current gaps in research, and to test our hypotheses, we conducted a controlled trial. The 10-week EU Climate Leadership Program was designed to identify changes in pro-environmental attitudes and actions across individual, collective and organizational levels. The program was part of the Inner Green Deal Initiative implemented by Awaris, an international leadership training institution with multiple branches in Europe, Canada, and Asia (The Inner Green Deal has since become an independent NGO). Five courses, each with around 20 participants, were conducted between March and July 2021 (for details, see supplemental information).

Participants in this study (N = 240) consisted of high-level decision-makers working in the field of sustainability and climate change, mainly from the European Commission, the European Parliament, other European, national or local policy institutions, and a multinational private company. The training company delivering these trainings (Awaris) had already worked within these organizations and established relationships with some human resources employees, which enabled them to advertise this new pilot training through their communication channels, such as the European Union’s internal learning website, and internal sustainability/leadership networks, as well as social media platforms.

Individuals interested in the course were offered detailed information on its content and requirements prior to registration. A screening survey ensured that vulnerable individuals were excluded from the intervention (defined as those who had experienced a mental health issue in the past six months and/or who currently felt overwhelmed, see supplemental information). All participants gave prior written informed consent to participate in this study.

Out of 240 recruited participants, 185 completed the *ex-ante* survey (77%). Of these, about half were in the intervention group (N = 94), and half in the control group (N = 91). The *ex-post* survey was completed by 120 participants (50% of the total sample, and 65% of the pre-survey sample). The attrition rate was higher in the control group (N = 42, 46%) than the intervention group (N = 23, 24%). In line with public and company data privacy regulations, no names or e-mail addresses were collected. Instead, participants were asked to create an anonymous key that was re-created as part of the *ex-post* survey, in order for their data to be matched across the two timepoints. The final sample was comprised of those who completed both surveys, and who could be matched using the personal key, yielding a total of 94 participants (N = 65 in the intervention group, and N = 29 in the control condition).

The majority of participants in both intervention and control groups (N = 94) identified as female (N = 68, 72.3%). Most were in the age range 35–54 (N = 58, 61%) and highly educated; most held a Master’s or postgraduate degree (N = 76, 80.9%). Given our research focus, the majority were working at the international or cross-country level (N = 85, 90%), in leadership/managerial positions (N = 42, 45%), in the public (N = 44, 47%), or the private (N = 39, 41%) sector. Participants in the intervention group were older, and more likely to be in a leadership position than those in the control group but did not differ regarding other key demographics. See supplemental information, Table S1 for descriptive statistics of our sample.

#### Design and procedure

Individuals in the intervention group participated in the EU Climate Leadership Program under the Inner Green Deal Initiative. The program consisted of seven, joint online sessions (modules), including an introductory session that was held one week prior to the official start. Linking mindfulness-based approaches with compassion, neuroscience, and behavioral science, the course was designed to systematically move from a focus on increasing awareness, (self-)care and compassion, and understanding habits, toward widening circles of identity, care, and responsibility. In the final two modules (6 and 7), the focus shifted more toward the organizational context. Developed in the form of an Action Lab, participants were invited to work on concrete societal and organizational challenges and discuss potential initiatives or working prototypes. Table 1 provides an overview of the modules and their objectives with respect to supporting inner and outer dimensions of transformation.

Sessions followed a similar structure to ensure a mix of reflective, contemplative, and co-creative aspects. A typical module contains the following aspects:

• Welcome and program overview.
• Meditation specific to the module (awareness, compassion, etc.) followed by group reflection.
• Review of content specific to the module (latest evidence on state of the planet, cognitive biases, working with emotions etc.).
• Break-out groups where participants reflect together on content, enter into dialogue or engage in an exercise to relate the content to their specific reality. The break-outs in later modules focus on collaboration on new sustainability initiatives that address specific societal and organizational needs.
• Group reflection and journalling to capture lessons learned.
• Next steps: review of home practice for the coming week (meditation, content on learning platform, habit change practices), actions (e.g., need to meet in focus groups to further develop initiatives).
• Check-out: sharing main insights and how people feel as they leave the session.

In addition to being required to participate in the modules, participants were asked to meet in smaller focus groups between the joint sessions. Here, the aim was to support perspective-taking and deepen the content of the modules. They were also required to incorporate a daily practice into their daily routine at home. This involved at least 10 min of formal mindfulness and/or compassion practice, along with informal exercises to support self-reflection regarding their beliefs, values, worldviews, habits, and associated change (such as paying attention to day-to-day routines, and weekly 1-h nature walks). The Awaris app, a cell phone application tailored to the needs of course participants, provided access to these practices. Finally, the program was supported by an online social learning platform which provided participants with course-related content and opportunities to support perspective-taking, interact, and learn from, and with, each other. Table 1 provides more details on the program and intervention.

As it was impossible to randomly assign participants to groups, due to administrative constraints resulting from the study’s field setting, a control group (N = 29) comprising a waitlist plus an active control group was recruited in parallel. Control group participants were mainly recruited from the same institutions as the intervention group (EU leadership networks, and the same multinational private company), and via social networks such as LinkedIn. For an overview of the sample structure and the differences between the learning activities of the intervention and active control groups, see Tables S3 and S4 in supplemental information.

#### Measures and reliability of scales

In order to test our hypotheses (cf. end of Section Research linking inner and outer transformation), participants filled in a pre- and post-program survey. This consisted of questions that probed aspects of mindfulness, compassion (toward the self, others, and nature), pro-environmental attitudes and behaviors, and their level of engagement to support change at organizational level (see supplemental information for the full questionnaire). All Cronbach alpha values reported below correspond to pre- and post-intervention values.^116^

##### Mindfulness

Mindfulness (α = 0.73/.77) was measured using a selection of items taken from the Five Facet Mindfulness Questionnaire.^117^ Discriminating between five dimensions of mindfulness (observing, describing, acting with awareness, non-judging of experiences, non-reactivity to experiences), the FFMQ is particularly suited to uncovering the underlying mechanisms of change in pro-environmentalism. In this context, the literature suggests that observing and non-reacting facets are the most relevant correlates of pro-environmentalism, while the effect of the describing dimension seems to be negligible.^74^ To reduce the burden on respondents, items related to the latter dimension were removed from our scale. The remaining facets were measured using two items per facet, yielding eight items that were rated on a five-point Likert-type scale ranging from 1 = *never or very rarely true* to 5 = *very often or always true*. Internal consistency of the additive subscales was good (acting with awareness, α = 0.60/.74; non-judgment, α = 0.90/.87; non-reacting, α = 0.67/.64).

##### Compassion (self, others, nature)

To assess the impact of self-compassion on pro-environmentalism, we administered six, slightly-rephrased items (to facilitate comparisons of responses) taken from the Self-Compassion Scale SCS^97^(α = 0.75/.73). Compassion toward others was measured using five items taken from the Compassionate Engagement and Action Scale^118^ (α = 0.72/.75). The latter scale assesses respondents’ relationship to others in general, and situations of distress in particular. Compassion with nature was assessed through participants' nature connectedness, using the visual version of the Inclusion of Nature in the Self Scale,^119^ which is both accurate and easy to assess. Participants were asked to choose among seven pairs of circles representing nature and the self, that overlapped to different degrees. Importantly, compassion toward others and nature-connectedness can be seen as transformative qualities/capacities, and as an expression of certain worldviews (cf. Section on Research linking inner and outer transformation).

##### Subjective wellbeing and climate anxiety

Wellbeing was assessed using the Mental Health Continuum–Short Form^120^ (α = 0.88/.89). This brief self-assessment tool combines three components of well-being: emotional, social, and psychological. Six of the items on the full 15-item scale were used. Participants were asked how often they had experienced certain mental and emotional states during the past month, with options ranging from 1 = *neve*r to 6 = *every day*. Climate anxiety was assessed by asking respondents how much they agreed with the following statement: “I often feel worry when I think about climate and environmental problems”, with options ranging from 1 = *strongly disagree* to 7 = *strongly agree*.

##### Pro-environmental attitudes

To assess participants’ attitudes toward the environment (α = 0.77/.72), we drew on a comprehensive set of self-report items that probed four dimensions of environmental attitudes, and their close correlates. The wording of these questions was harmonized so that all responses ranged from 1 = *strongly disagree* to 7 = *strongly agree*. The selected items probed: (a) environmental self-identity. The relationship to environmentalism was measured as environmental self-identity on a general level. Specifically, participants were asked to indicate their agreement with the statement “I see myself as an environmentally friendly person”.^121^ In addition, we included measurements of *(b) environmental concern* (α = 0.78/.74). Here, respondents were asked to indicate the threat climate change poses, and their emotional reaction to it. In this context, we also probed their perceived moral obligation to take action against climate change (for an overview of aggregated measures see supplemental information, Table S5). We also explored *(c) participants' willingness to pay on a personal level* (“I do what is good for the climate/environment even if this costs me more money or time”), and on an *(d) societal level* (“There needs to be stricter laws and regulations to protect the environment”) to assess their engagement with the environment in the face of trade-offs. Finally, *(e) beliefs about fighting climate change* (α = 0.79/.68) was assessed as respondents’ agreement with three statements referring to the role of the economy and science in climate change mitigation (e.g. “In order to protect the environment the country needs economic growth”). It should be noted that across all scales items were reversed so that higher scores indicated more pro-environmental attitudes. Finally, we added questions about moral obligation and perceptions regarding the role of the economy and science in addressing climate change. See supplemental information for the full questionnaire.

##### Pro-environmental behavior

To assess pro-environmental behavior (α = 0.87/.85), we asked participants to indicate how often they had engaged in 17 pro-environmental actions during the past two months. Possible responses ranged from 1 = *never or very rarely* to 5 = *very often or always*. These items covered the three dimensions proposed by Lynn^122^: behavior at home (e.g. minimizing waste; *waste* α = 0.69/.26), purchasing behavior (e.g. buying and eating organic; *food* α = 0.74/.74), and transport behavior (e.g. using public transportation; *transport* α = 0.72/.69). Items were selected based on their mitigation potential in the domains of food, transport, and housing.^123^ In addition, we probed participants’ engagement (*agency*; α = 0.83/.82) in environmental causes in a formal setting (e.g. voting for pro-environmental candidates), and informally (e.g. participation in protests). To probe the degree of adaptation to climate change, respondents were asked whether they “Take measures to be less affected/more prepared for climate impacts”.

##### Engagement at organizational level

In line with climate policy integration/mainstreaming theory,^124^ the integration of climate change considerations into the workplace was self-assessed (options: 1 = *not at all* to 5 = *fully*) with respect to three dimensions: *(a) Personal efforts to increase sustainability at work* (“To what extent do you stand up for climate action and seek to make sustainability central to your organization?”); *(b) The extent to which climate issues are considered in different organizational spheres* (“To what extent are climate issues considered/integrated in your current work and particularly in …”); and *(c) Personal integration intentions* (“To what extent do you intend to integrate climate change issues more in your work over the next 12 months?”). Response options ranged from: 1 = *no specific intentions/plans at this point* to 5 = *integrate fully/as much as possible*).

Table 2 provides an overview of pre- and post-intervention measures by group (intervention/control).

#### Data analysis

Our pilot study combined between- and within-group designs. Both designs have pros (e.g. fewer participants are needed, random noise is reduced) and cons (e.g., the minimization of learning effects) and are considered as complementary in experimental pilot studies.^125^^,^^126^ Within-group (repeated-measures) changes were assessed with paired *t*-tests, to account for the dependence of observations. These tests were crucially important because comparisons with the control group were not always possible, due to a lack of input data.

Between-group comparisons (which generally require a large number of participants) were run whenever possible, largely driven by data availability (see Table 2). Specifically, differences between participants in intervention and control conditions over time were assessed using a series of linear regression models. The pre-post change (*t*2 –*t*1) in the respective dependent variable was regressed on a treatment dummy.

As described in the introduction, our small-scale pilot study aimed to provide input to the design of larger, confirmatory trials. By nature, pilot studies often lack the power to achieve statistical significance at the usual 5% threshold.^38^ Therefore, and following statistical considerations for pilot studies in medical research, we also considered a Type I error rate (α-level) in the range of 0.20^127^ and 0.25^128^ when testing our hypotheses.

## Data Availability

• Anonymized intervention data have been deposited and are publicly available as of the date of publication. Data and code have been deposited at Zenodo https://doi.org/10.5281/ZENODO.7277332. • The study design and analysis plan was pre-registered in aspredicted.org. • Any additional information required to reanalyze the data reported in this paper is available from the lead contact upon request.

## References

[1] Masson-Delmotte V., Zhai P., Pirani A., Connors S.L., Péan C., Berger S., Caud N., Chen Y., Goldfarb L., Gomis M.I., IPCC (2021).

[2] UNEP (2022).

[3] Shukla P.R., Skea J., Calvo Buendia E., Masson-Delmotte V., Pörtner H.-O., Roberts D.C., Zhai P., Slade R., Connors S., van Diemen R., Ferrat M., IPCC (2019).

[4] Skea J., Shukla P., Reisinger A., Slade R., Pathak M., Khourdajie A., van Diemen R., Abdulla A., Akimoto K., Babiker M., Bai Q., Bashmakov I., Bataille C., IPCC (2022). Contribution of Working Group III to the Sixth Assessment Report of the Intergovernmental Panel on Climate Change.

[5] IPCC, Pörtner H.-O., Roberts D.C., Tignor M., Poloczanska E.S., Mintenbeck K., Alegría A., Craig M., Langsdorf S., Löschke S., Möller V., Okem A., Rama B. (2022). Contribution of Working Group II to the Sixth Assessment Report of the Intergovernmental Panel on Climate Change.

[6] Mundaca L., Sonnenschein J., Steg L., Höhne N., Ürge-Vorsatz D. (2019). The global expansion of climate mitigation policy interventions, the talanoa dialogue and the role of behavioural insights. Environ. Res. Commun..

[7] Leichenko R., O’Brien K. (2019).

[8] Wamsler C., Bristow J. (2022). At the intersection of mind and climate change: integrating inner dimensions of climate change into policymaking and practice. Clim. Change.

[9] Uhl I., Klackl J., Hansen N., Jonas E. (2018). Undesirable effects of threatening climate change information: a cross-cultural study. Group Process. Intergroup Relat..

[10] Wolfe S.E., Tubi A. (2019). Terror Management Theory and mortality awareness: a missing link in climate response studies? Wiley Interdisciplinary Reviews:. Clim. Change.

[11] Fritsche I., Hoppe A. (2018).

[12] Akil H., Robert-Demontrond P., Bouillé J. (2018). Exploitation of mortality salience in communication on climate change. Rech. Appl. Market..

[13] Brosch T. (2021). Affect and emotions as drivers of climate change perception and action: a review. Curr. Opin. Behav. Sci..

[14] Davidson D.J., Kecinski M. (2021). Emotional pathways to climate change responses. WIRE’s Climate Change.

[15] Ives C.D., Freeth R., Fischer J. (2020). Inside-out sustainability: the neglect of inner worlds. Ambio.

[16] O’Brien K. (2018). Is the 1.5°C target possible? Exploring the three spheres of transformation. Curr. Opin. Environ. Sustain..

[17] Wamsler C., Osberg G., Osika W., Herndersson H., Mundaca L. (2021). Linking internal and external transformation for sustainability and climate action: towards a new research and policy agenda. Global Environ. Change.

[18] Wamsler C., Bristow J., Cooper K., Steidle G., Taggart S., Søvold L., Bockler L., Oliver T.H., Legrand T. (2022). Report of the UNDP Conscious Food Systems Alliance (CoFSA).

[19] Druckman J.N., Lupia A. (2016). Preference change in competitive political environments. Annu. Rev. Polit. Sci..

[20] Carter D.M. (2011). Recognizing the role of positive emotions in fostering environmentally responsible behaviors. Ecopsychology.

[21] Fazey I., Schäpke N., Caniglia G., Patterson J., Hultman J., van Mierlo B., Säwe F., Wiek A., Wittmayer J., Aldunce P. (2018). Ten essentials for action-oriented and second order energy transitions, transformations and climate change research. Energy Res. Social Sci..

[22] Grušovnik T. (2012). Environmental denial: why we fail to change our environmentally damaging practices. Synth. Philos..

[23] Wamsler C., Schäpke N., Fraude C., Stasiak D., Bruhn T., Lawrence M., Schroeder H., Mundaca L. (2020). Enabling new mindsets and transformative skills for negotiating and activating climate action: lessons from UNFCCC conferences of the parties. Environ. Sci. Pol..

[24] Wamsler C. (2020). Education for sustainability: fostering a more conscious society and transformation towards sustainability. Int. J. Sustain. High Educ..

[25] Initiative I.D.G. (2021).

[26] UN (2021). https://unstats.un.org/sdgs/files/report/2021/secretary-general-sdg-report-2021%5fEN.pdf.

[27] Woiwode C., Schäpke N., Bina O., Veciana S., Kunze I., Parodi O., Schweizer-Ries P., Wamsler C. (2021). Inner transformation to sustainability as a deep leverage point: fostering new avenues for change through dialogue and reflection. Sustain. Sci..

[28] Bristow J., Bell R., Wamsler C. (2022).

[29] Richter, N., and Hunecke, M. (2022). Mindfulness, connectedness to nature, personal ecological norm and pro-environmental behavior: a daily diary study. Curr. Res. Ecol. Soc. Psychol. 3, 100038. 10.1016/j.cresp.2022.100038.

[30] Geiger S.M., Grossman P., Schrader U. (2019). Mindfulness and sustainability: correlation or causation?. Curr. Opin. Psychol..

[31] Sajjad A., Shahbaz W. (2020). Mindfulness and social sustainability: an integrative review. Soc. Indicat. Res..

[32] Thiermann U.B., Sheate W.R. (2020). The way forward in mindfulness and sustainability: a critical review and research agenda. J. Cogn. Enhanc..

[33] Wamsler C. (2018). Mind the gap: the role of mindfulness in adapting to increasing risk and climate change. Sustain. Sci..

[34] Wamsler C., Brossmann J., Hendersson H., Kristjansdottir R., McDonald C., Scarampi P. (2018). Mindfulness in sustainability science, practice, and teaching. Sustain. Sci..

[35] Böhme T., Stanszus L.S., Geiger S.M., Fischer D., Schrader U. (2018). Mindfulness training at school: away to engage adolescents with sustainable consumption?. Sustainability.

[36] Stanszus L., Fischer D., Böhme T., Frank P., Fritzsche J., Geiger S., Harfensteller J., Grossman P., Schrader U. (2017). Education for sustainable consumption through mindfulness training: development of a consumption-specific intervention. J. Teach. Educ. Sustain..

[37] Geiger S.M., Fischer D., Schrader U., Grossman P. (2020). Meditating for the planet: effects of a mindfulness-based intervention on sustainable consumption behaviors. Environ. Behav..

[38] Lee E.C., Whitehead A.L., Jacques R.M., Julious S.A. (2014). The statistical interpretation of pilot trials: should significance thresholds be reconsidered?. BMC Med. Res. Methodol..

[39] American Psychological Association (2010).

[40] Bamberg S., Möser G. (2007). Twenty years after Hines, Hungerford, and Tomera: a new meta-analysis of psycho-social determinants of pro-environmental behaviour. J. Environ. Psychol..

[41] Brundiers K., Barth M., Cebrián G., Cohen M., Diaz L., Doucette-Remington S., Dripps W., Habron G., Harré N., Jarchow M. (2021). Key competencies in sustainability in higher education—toward an agreed-upon reference framework. Sustain. Sci..

[42] Clayton S. (2019). Psychology and climate change. Curr. Biol..

[43] Clayton S., Manning C. (2018). Psychology and Climate Change: Human Perceptions, Impacts, and Responses 1st edition.

[44] Doherty T.J., Clayton S. (2011). The psychological impacts of global climate change. Am. Psychol..

[45] Hedlund-de Witt A., de Boer J., Boersema J.J. (2014). Exploring inner and outer worlds: a quantitative study of worldviews, environmental attitudes, and sustainable lifestyles. J. Environ. Psychol..

[46] Klöckner C.A. (2013). A comprehensive model of the psychology of environmental behaviour—a meta-analysis. Global Environ. Change.

[47] Parodi O., Tamm K. (2018). Personal sustainability: Exploring the far side of sustainable development.

[48] Schmitt M.T., Neufeld S.D., Mackay C.M.L., Dys-Steenbergen O. (2020). The perils of explaining climate inaction in terms of psychological barriers. J. Soc. Issues.

[49] Adger W.N., Barnett J., Brown K., Marshall N., O’Brien K. (2013). Cultural dimensions of climate change impacts and adaptation. Nat. Clim. Change.

[50] Dunlap R.E., McCright A.M., Yarosh J.H. (2016). The political divide on climate change: partisan polarization widens in the U.S. Environment.

[51] McCright A.M., Dunlap R.E., Marquart-Pyatt S.T. (2016). Political ideology and views about climate change in the European Union. Environ. Polit..

[52] Wesley Schultz P. (2001). The structure of environmental concern: concern for self, other people, and the biosphere. J. Environ. Psychol..

[53] Steg L., Vlek C. (2009). Encouraging pro-environmental behaviour: an integrative review and research agenda. J. Environ. Psychol..

[54] Milfont T.L., Duckitt J. (2010). The environmental attitudes inventory: a valid and reliable measure to assess the structure of environmental attitudes. J. Environ. Psychol..

[55] Lewis G.B., Palm R., Feng B. (2019). Cross-national variation in determinants of climate change concern. Environ. Polit..

[56] McCright A.M., Dunlap R.E. (2011). Cool dudes: the denial of climate change among conservative white males in the United States. Global Environ. Change.

[57] Ockwell D., Whitmarsh L., O’Neill S. (2009). Reorienting climate change communication for effective mitigation: forcing people to be green or fostering grass-roots engagement?. Sci. Commun..

[58] O'Brien K.L. (2016). Climate change and social transformations: is it time for a quantum leap?. WIREs Clim. Change.

[59] O’Brien K. (2021). Reflecting on the anthropocene: the call for deeper transformations. Ambio.

[60] Abson D.J., Fischer J., Leventon J., Newig J., Schomerus T., Vilsmaier U., von Wehrden H., Abernethy P., Ives C.D., Jager N.W., Lang D.J. (2017). Leverage points for sustainability transformation. Ambio.

[61] Meadows D. (1999).

[62] Kabat-Zinn J. (1994).

[63] Jha A.P., Krompinger J., Baime M.J. (2007). Mindfulness training modifies subsystems of attention. Cognit. Affect Behav. Neurosci..

[64] Lymeus F., Lundgren T., Hartig T. (2017). Attentional effort of beginning mindfulness training is offset with practice directed toward images of natural scenery. Environ. Behav..

[65] Arch J.J., Craske M.G. (2006). Mechanisms of mindfulness: emotion regulation following a focused breathing induction. Behav. Res. Ther..

[66] Hayes A.M., Feldman G., Gables C. (2004). Clarifying the construct of mindfulness in the context of emotion regulation and the process of change in Therapy. Clin. Psychol. Sci. Pract..

[67] Hölzel B.K., Lazar S.W., Gard T., Schuman-Olivier Z., Vago D.R., Ott U. (2011). How does mindfulness meditation work? Proposing mechanisms of action from a conceptual and neural perspective. Perspect. Psychol. Sci..

[68] Kerr C.E., Josyula K., Littenberg R. (2011). Developing an observing attitude: an analysis of meditation diaries in an MBSR clinical trial. Clin. Psychol. Psychother..

[69] Barragan-Jason G., de Mazancourt C., Parmesan C., Singer M.C., Loreau M. (2022). Human–nature connectedness as a pathway to sustainability: a global meta-analysis. Conserv. Lett..

[70] Fröding B., Osika W. (2015).

[71] Panno A., Giacomantonio M., Carrus G., Maricchiolo F., Pirchio S., Mannetti L. (2018). Mindfulness, pro-environmental behavior, and belief in climate change: the mediating role of social dominance. Environ. Behav..

[72] Wamsler C., Brink E. (2018). Mindsets for sustainability: exploring the link between mindfulness and sustainable climate adaptation. Ecol. Econ..

[73] Amel E.L., Manning C.M., Scott B.A. (2009). Mindfulness and sustainable behavior: pondering attention and awareness as means for increasing green behavior. Ecopsychology.

[74] Barbaro N., Pickett S.M. (2016). Mindfully green: examining the effect of connectedness to nature on the relationship between mindfulness and engagement in pro-environmental behavior. Pers. Indiv. Differ..

[75] Brown K.W., Kasser T., Ryan R.M., Alex Linley P., Orzech K. (2009). When what one has is enough: mindfulness, financial desire discrepancy, and subjective well-being. J. Res. Pers..

[76] Hanley A.W., Bettmann J.E., Kendrick C.E., Deringer A., Norton C.L. (2020). Dispositional mindfulness is associated with greater nature connectedness and self-reported ecological behavior. Ecopsychology.

[77] Loy L.S., Clemens A., Reese G. (2022). Mind–body practice is related to pro-environmental engagement through self-compassion and global identity rather than to self-enhancement. Mindfulness.

[78] Ray T.N., Franz S.A., Jarrett N.L., Pickett S.M. (2020). Nature Enhanced Meditation: Effects on mindfulness, connectedness to nature, and pro-environmental behavior. Environ. Behav..

[79] Birnie K., Speca M., Carlson L.E. (2010). Exploring self-compassion and empathy in the context of mindfulness-based stress reduction (MBSR). Stress Health.

[80] Shapiro S.L., Brown K.W., Biegel G.M. (2007). Teaching self-care to caregivers: effects of mindfulness-based stress reduction on the mental health of therapists in training. Training and Education in Professional Psychology.

[81] Ramstetter L. (2021). The political consequences of Be(com)ing mindful. How mindfulness might affect political attitudes. Front. Polit. Sci..

[82] Pfattheicher S., Sassenrath C., Schindler S. (2016). Feelings for the suffering of others and the environment: compassion fosters proenvironmental tendencies. Environ. Behav..

[83] Engel Y., Ramesh A., Steiner N. (2020). Powered by compassion: the effect of loving-kindness meditation on entrepreneurs’ sustainable decision-making. J. Bus. Ventur..

[84] Lu H., Schuldt J.P. (2016). Compassion for climate change victims and support for mitigation policy. J. Environ. Psychol..

[85] van Agteren J., Iasiello M., Lo L., Bartholomaeus J., Kopsaftis Z., Carey M., Kyrios M. (2021). A systematic review and meta-analysis of psychological interventions to improve mental wellbeing. Nat. Human Behav..

[86] Doppelt B. (2016).

[87] Baudon P., Jachens L. (2021). A scoping review of interventions for the treatment of eco-anxiety. Int. J. Environ. Res. Publ. Health.

[88] Soutar C., Wand A.P.F. (2022). Understanding the spectrum of anxiety responses to climate change: a systematic review of the qualitative literature. Int. J. Environ. Res. Publ. Health.

[89] Clayton S. (2020). Climate anxiety: psychological responses to climate change. J. Anxiety Disord..

[90] Moser S.C., Dillinger L., Moser S.C., Dillinger L. (2008). Creating a Climate for Change: Communicating Climate Change and Facilitating Social Change.

[91] Norgaard K.M. (2011).

[92] Britton W.B. (2019). Can mindfulness be too much of a good thing? The value of a middle way. Curr. Opin. Psychol..

[93] Schutte N.S., Malouff J.M. (2018). Mindfulness and connectedness to nature: a meta-analytic investigation. Pers. Indiv. Differ..

[94] Ives C.D., Giusti M., Fischer J., Abson D.J., Klaniecki K., Dorninger C., Laudan J., Barthel S., Abernethy P., Martín-López B. (2017). Human–nature connection: a multidisciplinary review. Curr. Opin. Environ. Sustain..

[95] Keng S.L., Smoski M.J., Robins C.J., Ekblad A.G., Brantley J.G. (2012). Mechanisms of change in mindfulness-based stress reduction: self-compassion and mindfulness as mediators of intervention outcomes. J. Cognit. Psychother..

[96] Taylor V.A., Grant J., Daneault V., Scavone G., Breton E., Roffe-Vidal S., Courtemanche J., Lavarenne A.S., Beauregard M. (2011). Impact of mindfulness on the neural responses to emotional pictures in experienced and beginner meditators. Neuroimage.

[97] Neff K.D. (2003). The development and validation of a scale to measure self-compassion. Self Ident..

[98] Chatzisarantis N.L.D., Hagger M.S. (2007). Mindfulness and the intention- behavior relationship within the theory of planned behavior. Pers. Soc. Psychol. Bull..

[99] Fredrickson B. (2003). The Value of Positive Emotions: The emerging science of positive psychology is coming to understand why it’s good to feel good. Am. Sci..

[100] Weber E.U. (2010). WIREs. Clim. Change.

[101] Stanszus L.S., Frank P., Geiger S.M. (2019). Healthy eating and sustainable nutrition through mindfulness? Mixed method results of a controlled intervention study. Appetite.

[102] Freedland K.E. (2020). Pilot trials in health-related behavioral intervention research: problems, solutions, and recommendations. Health Psychol..

[103] Thabane L., Ma J., Chu R., Cheng J., Ismaila A., Rios L.P., Robson R., Thabane M., Giangregorio L., Goldsmith C.H. (2010). A tutorial on pilot studies: the what, why and how. BMC Med. Res. Methodol..

[104] Awaris (2022).

[105] Brutus S., Aguinis H., Wassmer U. (2013). Self-reported limitations and future directions in scholarly reports: analysis and recommendations. J. Manag..

[106] Sharma M. (2017).

[107] Kisfalvi V., Oliver D. (2015). Creating and maintaining a safe space in experiential learning. J. Manag. Educ..

[108] Pereira L.M., Karpouzoglou T., Frantzeskaki N., Olsson P. (2018). Designing transformative spaces for sustainability in social-ecological systems. Ecol. Soc..

[109] Hards S. (2011). Social practice and the evolution of personal environmental values. Environ. Values.

[110] Stuckey B., Smith J.D., Hildreth P., Kimble C. (2004). Knowledge Networks: Innovation Through Communities Of Practice.

[111] Wenger E. (2010). An introduction to communities of practice. Encyclopedia of Knowledge Management.

[112] van Asselt H., Rayner T., Persson A., Bäckstrand K., Lövbrand E. (2015). Research Handbook on Climate Governance.

[113] Wamsler C., Osberg G. (2022). Transformative climate policy mainstreaming – engaging the political and the personal. Glob. Sustain..

[114] Djernis D., Lerstrup I., Poulsen D., Stigsdotter U., Dahlgaard J., O’toole M. (2019). A systematic review and meta-analysis of nature-based mindfulness: effects of moving mindfulness training into an outdoor natural setting. Int. J. Environ. Res. Publ. Health.

[115] Bristow J. (2019). Mindfulness in politics and public policy. Curr. Opin. Psychol..

[116] Heo M., Kim N., Faith M.S. (2015). Statistical power as a function of Cronbach alpha of instrument questionnaire items. BMC Med. Res. Methodol..

[117] Baer R.A., Smith G.T., Lykins E., Button D., Krietemeyer J., Sauer S., Walsh E., Duggan D., Williams J.M.G., Walsh E. (2008). Construct validity of the five facet mindfulness questionnaire in meditating and nonmeditating samples. Assessment.

[118] Gilbert P., Catarino F., Duarte C., Matos M., Kolts R., Stubbs J., Ceresatto L., Duarte J., Pinto-Gouveia J., Basran J. (2017). The development of compassionate engagement and action scales for self and others. J. Compassionate Health Care.

[119] Schultz W.P., Schmuck P., P. Wesley schultz, and T. L. Milfont (2002). Psychology Of Sustainable Development.

[120] Lamers S.M.A., Westerhof G.J., Bohlmeijer E.T., Ten Klooster P.M., Keyes C.L.M. (2011). Evaluating the psychometric properties of the mental health Continuum-Short Form (MHC-SF). J. Clin. Psychol..

[121] Van der Werff E., Steg L., Keizer K. (2013). The value of environmental self-identity: the relationship between biospheric values, environmental self-identity and environmental preferences, intentions and behaviour. J. Environ. Psychol..

[122] Lynn P. (2014). Distinguishing dimensions of pro-environmental behaviour. ISER working paper series.

[123] Ivanova D., Barrett J., Wiedenhofer D., Macura B., Callaghan M., Creutzig F. (2020). Quantifying the potential for climate change mitigation of consumption options. Environ. Res. Lett..

[124] Runhaar H., Wilk B., Persson Å., Uittenbroek C., Wamsler C. (2018). Mainstreaming climate adaptation: taking stock about “what works” from empirical research worldwide. Reg. Environ. Change.

[125] Zhang H., Zhang A., Liu C., Xiao J., Wang K. (2021). A brief online mindfulness-based group intervention for psychological distress among Chinese residents during COVID-19: a pilot randomized controlled trial. Mindfulness.

[126] Shearer J., Wodak A., Van Beek I., Mattick R.P., Lewis J. (2003). Pilot randomized double blind placebo-controlled study of dexamphetamine for cocaine dependence. Addiction.

[127] Stallard N. (2012). Optimal sample sizes for phase II clinical trials and pilot studies. Stat. Med..

[128] Schoenfeld D. (1980). Statistical considerations for pilot studies. Int. J. Radiat. Oncol. Biol. Phys..

