# Supplementary material for: Fostering collective climate action and leadership: Insights from a pilot experiment involving mindfulness and compassion
Source: iScience. 2023 Feb 24;26(3):106191. doi: 10.1016/j.isci.2023.106191 (PMC10040966; doi:10.1016/j.isci.2023.106191)

iScience, Volume *26*

Supplemental information

Fostering collective climate action and leadership: Insights from a pilot experiment involving mindfulness and compassion

Lena Ramstetter, Silke Rupprecht, Luis Mundaca, Walter Osika, Cecilia U.D. Stenfors, Johannes Klackl, and Christine Wamsler

Supplemental information

1 Survey

Data S1. Questionnaire. Related to STAR Methods

This survey helps you reflect on the linkage between mindset and environmental behaviour. It is part of a collaboration between the [redacted] and research from [redacted]. Survey completion takes approximately 10-15 minutes. The anonymous data will be analysed by [redacted]. Read the data privacy policy here. For more information about the [redacted]

First, please create your personal code. This code is extremely important to match your data across time points. Please save this code and/or create it according to the instructions below.

The code consists of five characters and is structured as follows: First two letters of your mother's first name (e.g. EM for Emma). Second numeral of your mother's day of birth (e.g. 6 for a birthday on January 06). First two letters of your father's first name (e.g. PE for Peter). The sample code would be EM6PE. Please enter your 5-digit personal code here:

Please enter your Project ID (e.g., E20-ABC-000, you can find it in your welcome letter)

First, please answer some demographic questions.

Your age
1. Under 18
2. 18-24
3. 25-34
4. 35-44
5. 45-54
6. 55-64
7. Over 64

Your gender
1. Female
2. Male
3. Other

Your highest education level
1. Primary and/or secondary school
2. High school
3. Bachelor’s degree
4. Technical/Vocational degree
5. Master’s degree
6. Postgraduate degree

Which best describes your professional role/ position?
1. Policymaker
2. Leader/ manager
3. Academic/ researcher
4. Advocate/ campaigner
5. Innovator/ entrepreneur

6. Writer/ thinker /communicator
7. Other __________

Which area do you work in primarily?
1. Parliament or other legislature
2. Public sector
3. Private sector
4. Non-governmental organisations
5. Education/ Academic sector

At what level do you mainly work?
1. Global level
2. EU level (or another cross-country region)
3. National level
4. Regional or local level (e.g. city)

Next, please reflect on your level of experience with mindfulness and other techniques.

Have you practiced some of the following techniques in the past?

	Yes	No
Moving meditation (such as Yoga, TaiChi, Qi gong, walking meditation).	❏	❏
Relaxation meditation (such as body scan, breathing or related mindfulness exercises).	❏	❏
Insight meditation (such as compassion, kindness or related mindfulness exercises).	❏	❏

Are you currently practicing some of these techniques?

	No	Yes, several times per year	Yes, several times per month	Yes, several times per week	Yes, daily
Moving meditation (such as Yoga, TaiChi, Qi gong, walking meditation).	❏	❏	❏	❏	❏
Relaxation meditation (such as body scan, breathing or related mindfulness exercises).	❏	❏	❏	❏	❏
Insight meditation (such as compassion, kindness or related mindfulness exercises).	❏	❏	❏	❏	❏

For how long have you been practicing the indicated techniques in the past (Please answer independently if you still practice or not).
1. Less than 1 year
2. 1-5 years
3. 5-10 years
4. More than 10 years
5. More than 20 years
6. Does not apply

Please choose the answer that best reflects your experience in the past month.

Below is a collection of statements about your everyday experience. Please indicate how frequently or infrequently you have had each experience in the past month. Please answer according to what really reflects your experience rather than what you think your experience should be.

	Never or very rarely true	Rarely true	Sometimes true	Often true	Very often or always true
In the past month, I felt difficulties were piling up so high that I could not overcome them.	❑	❑	❑	❑	❑
In general, I think that I can obtain outcomes that are important to me.	❑	❑	❑	❑	❑
I feel hopeful for the future.	❑	❑	❑	❑	❑

Please choose the response that best represents how often you have experienced or felt the following during the past month. How often have you felt ...

	Never	Once or twice	About once a week	About 2 or 3 times a week	Almost every day	Every day
... happy with life	❑	❑	❑	❑	❑	❑
... satisfied with life	❑	❑	❑	❑	❑	❑
... that you had something important to contribute to society	❑	❑	❑	❑	❑	❑
... that you belonged to a community (like a social group, or your neighborhood)	❑	❑	❑	❑	❑	❑
... that you had warm and trusting relationships with others	❑	❑	❑	❑	❑	❑
... that your life has a sense of direction or meaning to it	❑	❑	❑	❑	❑	❑

Please let us know about your experience with sustainability or climate change related issues

Do you work in sustainability and/or climate change related issues?
1. Yes
2. No

If yes, please specify (field of work, focus):

____

Please mark/ select the statements you most agree with

The most important thing in my life is …
1. To be successful and have people recognize my achievements
2. To be of service to my family, community and/or country
3. To do things my own way and forge my own path in life
4. To support my own and others' inner potential in the service of humanity

To what extent do you stand up for climate action and/or seek to make sustainability central to your organisation?

1. Fully
2. To a great extent
3. Partly
4. To a low extent
5. Not at all

To what extent are climate issues considered/ integrated in your current work, and particularly ….

	Fully	To a great extent	Partly	To a low extent	Not at all	Does not apply
... the type of activities/ projects you are involved in?	❑	❑	❑	❑	❑	❑
... your department/unit's field of operations (activities, projects, production processes)?	❑	❑	❑	❑	❑	❑
... the strategic priorities, aims and/or vision of your department/unit?	❑	❑	❑	❑	❑	❑
... your department/unit's internal regulations and policies?	❑	❑	❑	❑	❑	❑
... the planning, monitoring and evaluation tools you use?	❑	❑	❑	❑	❑	❑
... offered training, learning & development activities?	❑	❑	❑	❑	❑	❑
... internal working structures (e.g. groups or staff mandated to integrate the issue across sectors)?	❑	❑	❑	❑	❑	❑
... strategic cooperation with external stakeholders?	❑	❑	❑	❑	❑	❑
... budget allocations (financial resources)?	❑	❑	❑	❑	❑	❑
... human resource allocations?	❑	❑	❑	❑	❑	❑

What is your level of influence on ...

	Very high	Relatively high	Moderate	Relatively low	Very low
... the type of activities/ projects you are involved in?	❑	❑	❑	❑	❑
... your department/unit's field of operations (activities, projects, production processes)?	❑	❑	❑	❑	❑
... the strategic priorities, aims and/or vision of your department/unit?	❑	❑	❑	❑	❑
... your department/unit's internal regulations and policies?	❑	❑	❑	❑	❑
... the planning, monitoring and evaluation tools you use?	❑	❑	❑	❑	❑
... offered training, learning & development activities?	❑	❑	❑	❑	❑
... internal working structures (e.g. groups or staff mandated to integrate the issue across sectors)?	❑	❑	❑	❑	❑
... strategic cooperation with external stakeholders?	❑	❑	❑	❑	❑
... budget allocations (financial resources)?	❑	❑	❑	❑	❑
... human resource allocations?	❑	❑	❑	❑	❑

To what extent do you intend to integrate climate issues more in your work over the next 12 months?

1. No specific intentions/ plans at this point
2. Integrate fully – as much as possible
3. Integrate to a great extent
4. Integrate partly
5. Integrate to a low extent
6. Not relevant for my organisation

Please tell us for each of the following statements how much you agree or disagree

	agree strongly	agree to some extent	neutral/ neither agree nor disagree	disagree to some extent	disagree strongly
I see myself as an environmentally-friendly person.	❑	❑	❑	❑	❑
I do not see a big conflict between how environmentally friendly I want to live and how I actually live.	❑	❑	❑	❑	❑
If we continue like this, we will head for a climate/ an environmental catastrophe.	❑	❑	❑	❑	❑
I often feel worry when I think about climate and environmental problems. By "worry" we mean that it is something that weighs you down or makes you feel overwhelmed.	❑	❑	❑	❑	❑
It makes me sad when I think about the loss of species and territories due to global warming.	❑	❑	❑	❑	❑
It is just too difficult for someone like me to do much about the environment.	❑	❑	❑	❑	❑
Modern science will solve our environmental problems with little change to our way of living.	❑	❑	❑	❑	❑
There need to be stricter laws and regulations to protect the environment.	❑	❑	❑	❑	❑
In order to protect the environment the country needs economic growth.	❑	❑	❑	❑	❑
We worry too much about the future of the environment and not enough about prices and jobs.	❑	❑	❑	❑	❑
I do not see a big conflict between a good quality life and a sustainable life.	❑	❑	❑	❑	❑
I do what is good for the climate/ environment even if this costs me more money or time.	❑	❑	❑	❑	❑
I would feel guilty if I did not participate in actions to protect the environment/ address climate change.	❑	❑	❑	❑	❑
I feel morally obliged to participate in actions to protect the environment/ address climate change.	❑	❑	❑	❑	❑

Please tell us for each of the following statements how much you agree or disagree. Have you done any of the following in the past two months for environmental reasons?

	never or very rarely	rarely	sometimes	often	very often or always
Reduce car transport.	❑	❑	❑	❑	❑
Cut down on the amount I fly (e.g. no transatlantic and short-distance flights).	❑	❑	❑	❑	❑
Use public transportation whenever possible.	❑	❑	❑	❑	❑
Walk or cycle for non-recreational purposes.	❑	❑	❑	❑	❑
Adopt a vegetarian diet.	❑	❑	❑	❑	❑
Adopt a vegan diet.	❑	❑	❑	❑	❑
Buy/ eat organic food.	❑	❑	❑	❑	❑
Reduce material consumption (e.g. reusing, less clothes/ electronics, minimalist lifestyle).	❑	❑	❑	❑	❑
Minimize waste (e.g. food, packaging).	❑	❑	❑	❑	❑
Take measures to be less affected/ more prepared for climate impacts.	❑	❑	❑	❑	❑
Write a letter/email or signing a petition for supporting an environmental issue.	❑	❑	❑	❑	❑
Take part in a social movements/ protest about an environmental issue.	❑	❑	❑	❑	❑
Vote or support environmentally-oriented political candidates/ parties.	❑	❑	❑	❑	❑
Support environmental organizations/ groups (e.g. through donations or volunteering).	❑	❑	❑	❑	❑
Be an active member in an environmental organization.	❑	❑	❑	❑	❑
Actively spread awareness (e.g. through social media or other means).	❑	❑	❑	❑	❑
Create initiatives that addresses specific environmental needs/ challenges.	❑	❑	❑	❑	❑

Have you done any of the following in the past two months for environmental reasons?

	Yes	No
Live car-free.	❑	❑
Use a battery electric car.	❑	❑
Refurbish or renovate to reduce energy consumption.	❑	❑
Produce your own renewable electricity (e.g., solar PV).	❑	❑
Buy electricity generated with renewable energy sources (e.g., wind, solar).	❑	❑

2 Descriptives by group

Table S1. Sociodemographic Characteristics of the Final Sample (N=94). Related to STAR Methods

Variable	N	Overall (N = 94)[1]	Control (N = 29)[1]	Intervention (N = 65)[1]	p-value[2]
**Gender**	94				0.5
Female		68 (72%)	23 (79%)	45 (69%)	
Male		26 (28%)	6 (21%)	20 (31%)	
**Age**	94				0.024
25-34		21 (22%)	11 (38%)	10 (15%)	
35-44		23 (24%)	9 (31%)	14 (22%)	
45-54		35 (37%)	5 (17%)	30 (46%)	
55-64		14 (15%)	4 (14%)	10 (15%)	
Over 64		1 (1.1%)	0 (0%)	1 (1.5%)	
**Education**	94				0.089
High School		2 (2.1%)	2 (6.9%)	0 (0%)	
Bachelor's degree		15 (16%)	6 (21%)	9 (14%)	
Technical/Vocational degree		1 (1.1%)	1 (3.4%)	0 (0%)	
Master's degree		55 (59%)	14 (48%)	41 (63%)	
Postgraduate degree		21 (22%)	6 (21%)	15 (23%)	
**Area of work**	94				0.3
Parliament or other legislature		2 (2.1%)	1 (3.4%)	1 (1.5%)	
Public sector		44 (47%)	15 (52%)	29 (45%)	
Private sector		39 (41%)	13 (45%)	26 (40%)	
Non-governmental organisations		6 (6.4%)	0 (0%)	6 (9.2%)	
Education/Academic sector		3 (3.2%)	0 (0%)	3 (4.6%)	
**Role at work**	94				<0.001
Policymaker		6 (6.4%)	5 (17%)	1 (1.5%)	
Leader/manager		42 (45%)	5 (17%)	37 (57%)	
Academic/ researcher		6 (6.4%)	3 (10%)	3 (4.6%)	
Advocate/campaigner		4 (4.3%)	3 (10%)	1 (1.5%)	
Innovater/entrepreneur		5 (5.3%)	0 (0%)	5 (7.7%)	
Writer/thinker/communicator		10 (11%)	4 (14%)	6 (9.2%)	
other		21 (22%)	9 (31%)	12 (18%)	
**Level of work**	94				0.10
Global level		36 (38%)	8 (28%)	28 (43%)	

EU level (or another cross-country region)	49 (52%)	16 (55%)	33 (51%)
National level	7 (7.4%)	3 (10%)	4 (6.2%)
Regional or local level (e.g., city)	2 (2.1%)	2 (6.9%)	0 (0%)

[1] Mean (SD); n (%)

[2] Wilcoxon rank sum test; Fisher's exact test

Table S2. Descriptive statistics by group (complete). Related to STAR Methods

	Control (N=29)	Intervention (N=65)	Overall (N=94)	P-value
t1_Age				
Mean (SD)	4.07 (1.07)	4.66 (0.973)	4.48 (1.03)	0.0357
Median [Min, Max]	4.00 [3.00, 6.00]	5.00 [3.00, 7.00]	5.00 [3.00, 7.00]	
female				
1	23 (79.3%)	45 (69.2%)	68 (72.3%)	NA
0	6 (20.7%)	20 (30.8%)	26 (27.7%)	
t1_Edu				
Mean (SD)	4.55 (1.24)	4.95 (0.891)	4.83 (1.02)	0.211
Median [Min, Max]	5.00 [2.00, 6.00]	5.00 [3.00, 6.00]	5.00 [2.00, 6.00]	
t1_Role				
Mean (SD)	4.24 (2.42)	3.58 (2.11)	3.79 (2.22)	0.416
Median [Min, Max]	4.00 [1.00, 7.00]	2.00 [1.00, 7.00]	2.00 [1.00, 7.00]	
t1_Area				
Mean (SD)	2.41 (0.568)	2.71 (0.843)	2.62 (0.777)	0.238
Median [Min, Max]	2.00 [1.00, 3.00]	3.00 [1.00, 5.00]	3.00 [1.00, 5.00]	
t1_Level				
Mean (SD)	1.97 (0.823)	1.63 (0.601)	1.73 (0.691)	0.0932
Median [Min, Max]	2.00 [1.00, 4.00]	2.00 [1.00, 3.00]	2.00 [1.00, 4.00]	
intervention				
0	29 (100%)	0 (0%)	29 (30.9%)	<0.001
1	0 (0%)	65 (100%)	65 (69.1%)	
course				
Control_active	14 (48.3%)	0 (0%)	14 (14.9%)	<0.001
Control_waitlist	15 (51.7%)	0 (0%)	15 (16.0%)	

	Control (N=29)	Intervention (N=65)	Overall (N=94)	P-value
Awaris	0 (0%)	19 (29.2%)	19 (20.2%)	
EU	0 (0%)	29 (44.6%)	29 (30.9%)	
Lanxess	0 (0%)	17 (26.2%)	17 (18.1%)	
t1_PID				
D21-CTR-001	4 (13.8%)	0 (0%)	4 (4.3%)	<0.001
D21-EUC-076	14 (48.3%)	0 (0%)	14 (14.9%)	
D21-LAX-083	11 (37.9%)	0 (0%)	11 (11.7%)	
D21AWA054	0 (0%)	6 (9.2%)	6 (6.4%)	
D21AWA066	0 (0%)	13 (20.0%)	13 (13.8%)	
D21EUC008	0 (0%)	29 (44.6%)	29 (30.9%)	
D21LAX010	0 (0%)	17 (26.2%)	17 (18.1%)	
t2_PID				
D21-CTR-001	4 (13.8%)	0 (0%)	4 (4.3%)	<0.001
D21-EUC-076	14 (48.3%)	0 (0%)	14 (14.9%)	
D21-LAX-083	11 (37.9%)	0 (0%)	11 (11.7%)	
D21AWA054	0 (0%)	6 (9.2%)	6 (6.4%)	
D21AWA066	0 (0%)	13 (20.0%)	13 (13.8%)	
D21EUC008	0 (0%)	29 (44.6%)	29 (30.9%)	
D21LAX010	0 (0%)	17 (26.2%)	17 (18.1%)	
t1_PEAtt_1r				
Mean (SD)	4.34 (0.670)	4.25 (0.685)	4.28 (0.678)	0.81
Median [Min, Max]	4.00 [3.00, 5.00]	4.00 [2.00, 5.00]	4.00 [2.00, 5.00]	
t1_PEAtt_2				
Mean (SD)	2.76 (1.18)	2.95 (1.11)	2.89 (1.13)	0.743
Median [Min, Max]	3.00 [1.00, 5.00]	3.00 [1.00, 5.00]	3.00 [1.00, 5.00]	
t1_PEAtt_3r				
Mean (SD)	4.52 (0.634)	4.68 (0.562)	4.63 (0.586)	0.476
Median [Min, Max]	5.00 [3.00, 5.00]	5.00 [3.00, 5.00]	5.00 [3.00, 5.00]	
t1_PEAtt_4r				
Mean (SD)	3.48 (1.33)	3.82 (1.06)	3.71 (1.15)	0.433
Median [Min, Max]	4.00 [1.00, 5.00]	4.00 [1.00, 5.00]	4.00 [1.00, 5.00]	
t1_PEAtt_5r				
Mean (SD)	4.38 (0.942)	4.72 (0.545)	4.62 (0.705)	0.0905

	Control (N=29)	Intervention (N=65)	Overall (N=94)	P-value
Median [Min, Max]	5.00 [1.00, 5.00]	5.00 [3.00, 5.00]	5.00 [1.00, 5.00]	
t1_PEAtt_6				
Mean (SD)	3.62 (1.12)	3.69 (1.03)	3.67 (1.05)	0.955
Median [Min, Max]	4.00 [1.00, 5.00]	4.00 [2.00, 5.00]	4.00 [1.00, 5.00]	
t1_PEAtt_7				
Mean (SD)	4.03 (0.731)	4.15 (0.988)	4.12 (0.914)	0.844
Median [Min, Max]	4.00 [3.00, 5.00]	4.00 [1.00, 5.00]	4.00 [1.00, 5.00]	
t1_PEAtt_8r				
Mean (SD)	4.38 (0.775)	4.60 (0.494)	4.53 (0.599)	0.256
Median [Min, Max]	5.00 [2.00, 5.00]	5.00 [4.00, 5.00]	5.00 [2.00, 5.00]	
t1_PEAtt_9				
Mean (SD)	3.10 (0.976)	3.58 (1.07)	3.43 (1.06)	0.132
Median [Min, Max]	3.00 [1.00, 5.00]	4.00 [1.00, 5.00]	3.00 [1.00, 5.00]	
Missing	0 (0%)	1 (1.5%)	1 (1.1%)	
t1_PEAtt_10				
Mean (SD)	4.00 (0.926)	4.35 (0.765)	4.24 (0.830)	0.171
Median [Min, Max]	4.00 [2.00, 5.00]	5.00 [2.00, 5.00]	4.00 [2.00, 5.00]	
Missing	0 (0%)	2 (3.1%)	2 (2.1%)	
t1_PEAtt_11r				
Mean (SD)	3.55 (1.12)	4.11 (0.986)	3.93 (1.06)	0.0602
Median [Min, Max]	4.00 [1.00, 5.00]	4.00 [1.00, 5.00]	4.00 [1.00, 5.00]	
Missing	0 (0%)	2 (3.1%)	2 (2.1%)	
t1_PEAtt_12r				
Mean (SD)	3.79 (0.620)	4.00 (0.873)	3.94 (0.805)	0.518
Median [Min, Max]	4.00 [3.00, 5.00]	4.00 [1.00, 5.00]	4.00 [1.00, 5.00]	
Missing	0 (0%)	1 (1.5%)	1 (1.1%)	
t1_PEAtt_13r				
Mean (SD)	3.76 (0.872)	3.73 (1.03)	3.74 (0.982)	0.992
Median [Min, Max]	4.00 [2.00, 5.00]	4.00 [1.00, 5.00]	4.00 [1.00, 5.00]	
Missing	0 (0%)	2 (3.1%)	2 (2.1%)	

	Control (N=29)	Intervention (N=65)	Overall (N=94)	P-value
t1_PEAtt_14r				
Mean (SD)	3.62 (0.942)	4.02 (0.924)	3.89 (0.943)	0.174
Median [Min, Max]	4.00 [2.00, 5.00]	4.00 [2.00, 5.00]	4.00 [2.00, 5.00]	
Missing	0 (0%)	2 (3.1%)	2 (2.1%)	
t1_pea_sum				
Mean (SD)	43.4 (5.45)	45.9 (5.08)	45.1 (5.30)	0.112
Median [Min, Max]	44.0 [30.0, 53.0]	46.0 [31.0, 55.0]	45.0 [30.0, 55.0]	
Missing	0 (0%)	3 (4.6%)	3 (3.2%)	
t1_pea_ec				
Mean (SD)	19.8 (3.50)	21.0 (2.96)	20.6 (3.17)	0.218
Median [Min, Max]	20.0 [9.00, 25.0]	21.0 [14.0, 25.0]	21.0 [9.00, 25.0]	
Missing	0 (0%)	2 (3.1%)	2 (2.1%)	
t1_pea_fcc				
Mean (SD)	11.1 (2.07)	12.1 (2.22)	11.8 (2.21)	0.163
Median [Min, Max]	11.0 [8.00, 15.0]	12.0 [7.00, 15.0]	12.0 [7.00, 15.0]	
Missing	0 (0%)	2 (3.1%)	2 (2.1%)	
t1_pea_esi				
Mean (SD)	4.34 (0.670)	4.25 (0.685)	4.28 (0.678)	0.81
Median [Min, Max]	4.00 [3.00, 5.00]	4.00 [2.00, 5.00]	4.00 [2.00, 5.00]	
t1_pea_wtpp				
Mean (SD)	3.79 (0.620)	4.00 (0.873)	3.94 (0.805)	0.518
Median [Min, Max]	4.00 [3.00, 5.00]	4.00 [1.00, 5.00]	4.00 [1.00, 5.00]	
Missing	0 (0%)	1 (1.5%)	1 (1.1%)	
t1_pea_wtps				
Mean (SD)	4.38 (0.775)	4.60 (0.494)	4.53 (0.599)	0.256
Median [Min, Max]	5.00 [2.00, 5.00]	5.00 [4.00, 5.00]	5.00 [2.00, 5.00]	
t2_PEAtt_1r				
Mean (SD)	4.17 (0.602)	4.32 (0.709)	4.28 (0.678)	0.611
Median [Min, Max]	4.00 [3.00, 5.00]	4.00 [1.00, 5.00]	4.00 [1.00, 5.00]	
t2_PEAtt_2				
Mean (SD)	2.90 (1.08)	2.94 (1.04)	2.93 (1.05)	0.984

	Control (N=29)	Intervention (N=65)	Overall (N=94)	P-value
Median [Min, Max]	3.00 [1.00, 5.00]	3.00 [1.00, 5.00]	3.00 [1.00, 5.00]	
t2_PEAtt_3r				
Mean (SD)	4.52 (0.688)	4.60 (0.725)	4.57 (0.711)	0.873
Median [Min, Max]	5.00 [3.00, 5.00]	5.00 [2.00, 5.00]	5.00 [2.00, 5.00]	
t2_PEAtt_4r				
Mean (SD)	3.34 (1.11)	3.37 (1.10)	3.36 (1.10)	0.995
Median [Min, Max]	4.00 [1.00, 5.00]	3.00 [1.00, 5.00]	3.50 [1.00, 5.00]	
t2_PEAtt_5r				
Mean (SD)	4.24 (0.912)	4.57 (0.706)	4.47 (0.786)	0.173
Median [Min, Max]	4.00 [2.00, 5.00]	5.00 [1.00, 5.00]	5.00 [1.00, 5.00]	
t2_PEAtt_6				
Mean (SD)	3.59 (0.780)	3.80 (1.16)	3.73 (1.06)	0.666
Median [Min, Max]	4.00 [2.00, 5.00]	4.00 [1.00, 5.00]	4.00 [1.00, 5.00]	
t2_PEAtt_7				
Mean (SD)	4.07 (0.842)	4.14 (1.06)	4.12 (0.993)	0.952
Median [Min, Max]	4.00 [2.00, 5.00]	4.00 [1.00, 5.00]	4.00 [1.00, 5.00]	
t2_PEAtt_8r				
Mean (SD)	4.14 (1.06)	4.45 (0.884)	4.35 (0.947)	0.346
Median [Min, Max]	4.00 [1.00, 5.00]	5.00 [1.00, 5.00]	5.00 [1.00, 5.00]	
t2_PEAtt_9				
Mean (SD)	3.03 (1.12)	3.56 (1.05)	3.40 (1.09)	0.0965
Median [Min, Max]	3.00 [1.00, 5.00]	4.00 [1.00, 5.00]	3.00 [1.00, 5.00]	
Missing	0 (0%)	1 (1.5%)	1 (1.1%)	
t2_PEAtt_10				
Mean (SD)	3.90 (1.11)	4.14 (0.982)	4.06 (1.02)	0.573
Median [Min, Max]	4.00 [1.00, 5.00]	4.00 [1.00, 5.00]	4.00 [1.00, 5.00]	
t2_PEAtt_11r				
Mean (SD)	3.66 (0.897)	4.08 (1.15)	3.95 (1.09)	0.223
Median [Min, Max]	4.00 [2.00, 5.00]	4.00 [1.00, 5.00]	4.00 [1.00, 5.00]	
t2_PEAtt_12r				

	Control (N=29)	Intervention (N=65)	Overall (N=94)	P-value
Mean (SD)	3.76 (0.689)	4.08 (0.620)	3.98 (0.655)	0.0924
Median [Min, Max]	4.00 [2.00, 5.00]	4.00 [2.00, 5.00]	4.00 [2.00, 5.00]	
t2_PEAtt_13r				
Mean (SD)	3.28 (1.03)	3.82 (1.09)	3.65 (1.09)	0.0858
Median [Min, Max]	4.00 [1.00, 5.00]	4.00 [1.00, 5.00]	4.00 [1.00, 5.00]	
t2_PEAtt_14r				
Mean (SD)	3.34 (1.08)	3.91 (1.13)	3.73 (1.14)	0.0842
Median [Min, Max]	4.00 [1.00, 5.00]	4.00 [1.00, 5.00]	4.00 [1.00, 5.00]	
t2_pea_sum				
Mean (SD)	41.8 (5.12)	45.0 (5.19)	44.0 (5.35)	0.0291
Median [Min, Max]	42.0 [30.0, 53.0]	45.0 [30.0, 55.0]	44.0 [30.0, 55.0]	
Missing	0 (0%)	1 (1.5%)	1 (1.1%)	
t2_pea_ec				
Mean (SD)	18.7 (3.46)	20.3 (3.42)	19.8 (3.49)	0.141
Median [Min, Max]	19.0 [11.0, 25.0]	21.0 [8.00, 25.0]	20.0 [8.00, 25.0]	
t2_pea_fcc				
Mean (SD)	11.0 (2.02)	11.9 (2.15)	11.6 (2.14)	0.175
Median [Min, Max]	10.0 [8.00, 15.0]	12.0 [7.00, 15.0]	12.0 [7.00, 15.0]	
Missing	0 (0%)	1 (1.5%)	1 (1.1%)	
t2_pea_esi				
Mean (SD)	4.17 (0.602)	4.32 (0.709)	4.28 (0.678)	0.611
Median [Min, Max]	4.00 [3.00, 5.00]	4.00 [1.00, 5.00]	4.00 [1.00, 5.00]	
t2_pea_wtpp				
Mean (SD)	3.76 (0.689)	4.08 (0.620)	3.98 (0.655)	0.0924
Median [Min, Max]	4.00 [2.00, 5.00]	4.00 [2.00, 5.00]	4.00 [2.00, 5.00]	
t2_pea_wtps				
Mean (SD)	4.14 (1.06)	4.45 (0.884)	4.35 (0.947)	0.346
Median [Min, Max]	4.00 [1.00, 5.00]	5.00 [1.00, 5.00]	5.00 [1.00, 5.00]	
t1_PEA_1				
Mean (SD)	3.52 (1.35)	3.72 (1.10)	3.66 (1.18)	0.737

	Control (N=29)	Intervention (N=65)	Overall (N=94)	P-value
Median [Min, Max]	4.00 [1.00, 5.00]	4.00 [1.00, 5.00]	4.00 [1.00, 5.00]	
t1_PEA_2				
Mean (SD)	3.38 (1.50)	3.73 (1.20)	3.62 (1.30)	0.476
Median [Min, Max]	4.00 [1.00, 5.00]	4.00 [1.00, 5.00]	4.00 [1.00, 5.00]	
Missing	0 (0%)	1 (1.5%)	1 (1.1%)	
t1_PEA_3				
Mean (SD)	3.62 (1.59)	3.73 (1.22)	3.70 (1.34)	0.931
Median [Min, Max]	4.00 [1.00, 5.00]	4.00 [1.00, 5.00]	4.00 [1.00, 5.00]	
Missing	0 (0%)	1 (1.5%)	1 (1.1%)	
t1_PEA_4				
Mean (SD)	3.66 (1.26)	4.12 (1.13)	3.98 (1.18)	0.207
Median [Min, Max]	4.00 [1.00, 5.00]	4.00 [1.00, 5.00]	4.00 [1.00, 5.00]	
t1_PEA_5				
Mean (SD)	3.03 (1.50)	3.54 (1.23)	3.38 (1.33)	0.236
Median [Min, Max]	3.00 [1.00, 5.00]	4.00 [1.00, 5.00]	4.00 [1.00, 5.00]	
t1_PEA_6				
Mean (SD)	2.00 (1.28)	2.52 (1.32)	2.36 (1.33)	0.209
Median [Min, Max]	1.00 [1.00, 5.00]	2.00 [1.00, 5.00]	2.00 [1.00, 5.00]	
t1_PEA_7				
Mean (SD)	3.66 (0.936)	3.98 (1.12)	3.88 (1.08)	0.391
Median [Min, Max]	4.00 [1.00, 5.00]	4.00 [1.00, 5.00]	4.00 [1.00, 5.00]	
t1_PEA_8				
Mean (SD)	3.48 (0.949)	3.78 (0.812)	3.68 (0.864)	0.313
Median [Min, Max]	4.00 [2.00, 5.00]	4.00 [2.00, 5.00]	4.00 [2.00, 5.00]	
Missing	0 (0%)	2 (3.1%)	2 (2.1%)	
t1_PEA_9				
Mean (SD)	3.45 (0.870)	3.85 (0.815)	3.72 (0.848)	0.108
Median [Min, Max]	3.00 [2.00, 5.00]	4.00 [2.00, 5.00]	4.00 [2.00, 5.00]	
t1_PEA_10				
Mean (SD)	2.17 (0.889)	2.63 (1.08)	2.48 (1.04)	0.149

	Control (N=29)	Intervention (N=65)	Overall (N=94)	P-value
Median [Min, Max]	2.00 [1.00, 4.00]	2.00 [1.00, 5.00]	2.00 [1.00, 5.00]	
Missing	0 (0%)	1 (1.5%)	1 (1.1%)	
t1_PEA_11				
Mean (SD)	2.00 (1.13)	2.52 (1.26)	2.36 (1.24)	0.168
Median [Min, Max]	2.00 [1.00, 5.00]	2.00 [1.00, 5.00]	2.00 [1.00, 5.00]	
t1_PEA_12				
Mean (SD)	1.62 (1.08)	2.17 (1.28)	2.00 (1.24)	0.141
Median [Min, Max]	1.00 [1.00, 5.00]	2.00 [1.00, 5.00]	1.00 [1.00, 5.00]	
t1_PEA_13				
Mean (SD)	2.86 (1.57)	3.95 (1.26)	3.62 (1.45)	0.00281
Median [Min, Max]	3.00 [1.00, 5.00]	4.00 [1.00, 5.00]	4.00 [1.00, 5.00]	
t1_PEA_14				
Mean (SD)	2.34 (1.40)	3.22 (1.34)	2.95 (1.41)	0.0205
Median [Min, Max]	2.00 [1.00, 5.00]	3.00 [1.00, 5.00]	3.00 [1.00, 5.00]	
t1_PEA_15				
Mean (SD)	1.38 (1.08)	2.14 (1.36)	1.90 (1.32)	0.0345
Median [Min, Max]	1.00 [1.00, 5.00]	2.00 [1.00, 5.00]	1.00 [1.00, 5.00]	
Missing	0 (0%)	1 (1.5%)	1 (1.1%)	
t1_PEA_16				
Mean (SD)	2.17 (1.34)	2.97 (1.31)	2.72 (1.36)	0.0311
Median [Min, Max]	2.00 [1.00, 5.00]	3.00 [1.00, 5.00]	3.00 [1.00, 5.00]	
t1_PEA_17				
Mean (SD)	1.38 (0.979)	2.35 (1.27)	2.05 (1.26)	0.0022
Median [Min, Max]	1.00 [1.00, 5.00]	2.00 [1.00, 5.00]	1.50 [1.00, 5.00]	
t1_PEA_18				
Mean (SD)	1.72 (0.455)	1.71 (0.458)	1.71 (0.455)	0.987
Median [Min, Max]	2.00 [1.00, 2.00]	2.00 [1.00, 2.00]	2.00 [1.00, 2.00]	
t1_PEA_19				
Mean (SD)	1.93 (0.258)	1.85 (0.364)	1.87 (0.336)	0.527

	Control (N=29)	Intervention (N=65)	Overall (N=94)	P-value
Median [Min, Max]	2.00 [1.00, 2.00]	2.00 [1.00, 2.00]	2.00 [1.00, 2.00]	
t1_PEA_20				
Mean (SD)	1.79 (0.412)	1.49 (0.504)	1.59 (0.495)	0.0235
Median [Min, Max]	2.00 [1.00, 2.00]	1.00 [1.00, 2.00]	2.00 [1.00, 2.00]	
t1_PEA_21				
Mean (SD)	1.90 (0.310)	1.85 (0.364)	1.86 (0.347)	0.81
Median [Min, Max]	2.00 [1.00, 2.00]	2.00 [1.00, 2.00]	2.00 [1.00, 2.00]	
t1_PEA_22				
Mean (SD)	1.45 (0.506)	1.47 (0.503)	1.46 (0.501)	0.984
Median [Min, Max]	1.00 [1.00, 2.00]	1.00 [1.00, 2.00]	1.00 [1.00, 2.00]	
Missing	0 (0%)	1 (1.5%)	1 (1.1%)	
t1_peb_sum				
Mean (SD)	45.7 (10.2)	55.0 (11.2)	52.0 (11.7)	0.00162
Median [Min, Max]	45.0 [26.0, 72.0]	54.5 [26.0, 81.0]	52.0 [26.0, 81.0]	
Missing	0 (0%)	3 (4.6%)	3 (3.2%)	
t1_peb_transport				
Mean (SD)	14.2 (4.22)	15.3 (3.47)	14.9 (3.74)	0.414
Median [Min, Max]	15.0 [5.00, 20.0]	16.0 [4.00, 20.0]	16.0 [4.00, 20.0]	
Missing	0 (0%)	2 (3.1%)	2 (2.1%)	
t1_peb_food				
Mean (SD)	8.69 (2.99)	10.0 (3.06)	9.63 (3.09)	0.143
Median [Min, Max]	8.00 [3.00, 14.0]	10.0 [3.00, 15.0]	10.0 [3.00, 15.0]	
t1_peb_waste				
Mean (SD)	6.93 (1.58)	7.62 (1.31)	7.40 (1.43)	0.0988
Median [Min, Max]	7.00 [4.00, 10.0]	8.00 [5.00, 10.0]	7.00 [4.00, 10.0]	
Missing	0 (0%)	2 (3.1%)	2 (2.1%)	
t1_peb_adapt				
Mean (SD)	2.17 (0.889)	2.63 (1.08)	2.48 (1.04)	0.149
Median [Min, Max]	2.00 [1.00, 4.00]	2.00 [1.00, 5.00]	2.00 [1.00, 5.00]	
Missing	0 (0%)	1 (1.5%)	1 (1.1%)	

	Control (N=29)	Intervention (N=65)	Overall (N=94)	P-value
t1_peb_agency				
Mean (SD)	13.8 (6.53)	19.5 (6.44)	17.7 (6.96)	0.00101
Median [Min, Max]	12.0 [7.00, 32.0]	19.5 [7.00, 34.0]	17.0 [7.00, 34.0]	
Missing	0 (0%)	1 (1.5%)	1 (1.1%)	
t2_PEA_1				
Mean (SD)	3.97 (0.944)	3.72 (1.02)	3.80 (1.00)	0.556
Median [Min, Max]	4.00 [1.00, 5.00]	4.00 [1.00, 5.00]	4.00 [1.00, 5.00]	
t2_PEA_2				
Mean (SD)	3.83 (1.34)	4.09 (1.27)	4.01 (1.29)	0.657
Median [Min, Max]	4.00 [1.00, 5.00]	5.00 [1.00, 5.00]	4.00 [1.00, 5.00]	
t2_PEA_3				
Mean (SD)	3.41 (1.40)	3.80 (1.25)	3.68 (1.31)	0.416
Median [Min, Max]	4.00 [1.00, 5.00]	4.00 [1.00, 5.00]	4.00 [1.00, 5.00]	
t2_PEA_4				
Mean (SD)	3.97 (1.24)	4.17 (1.07)	4.11 (1.12)	0.719
Median [Min, Max]	4.00 [1.00, 5.00]	5.00 [1.00, 5.00]	4.00 [1.00, 5.00]	
t2_PEA_5				
Mean (SD)	3.03 (1.40)	3.56 (1.23)	3.40 (1.30)	0.193
Median [Min, Max]	3.00 [1.00, 5.00]	4.00 [1.00, 5.00]	4.00 [1.00, 5.00]	
Missing	0 (0%)	1 (1.5%)	1 (1.1%)	
t2_PEA_6				
Mean (SD)	2.10 (1.29)	2.72 (1.34)	2.53 (1.35)	0.124
Median [Min, Max]	2.00 [1.00, 5.00]	3.00 [1.00, 5.00]	2.00 [1.00, 5.00]	
Missing	0 (0%)	1 (1.5%)	1 (1.1%)	
t2_PEA_7				
Mean (SD)	3.62 (0.775)	3.98 (1.07)	3.87 (0.997)	0.262
Median [Min, Max]	4.00 [2.00, 5.00]	4.00 [1.00, 5.00]	4.00 [1.00, 5.00]	
t2_PEA_8				
Mean (SD)	3.66 (0.857)	3.95 (0.785)	3.86 (0.815)	0.263
Median [Min, Max]	4.00 [1.00, 5.00]	4.00 [2.00, 5.00]	4.00 [1.00, 5.00]	

	Control (N=29)	Intervention (N=65)	Overall (N=94)	P-value
Missing	0 (0%)	1 (1.5%)	1 (1.1%)	
t2_PEA_9				
Mean (SD)	3.62 (0.903)	3.82 (0.827)	3.76 (0.851)	0.593
Median [Min, Max]	4.00 [2.00, 5.00]	4.00 [2.00, 5.00]	4.00 [2.00, 5.00]	
t2_PEA_10				
Mean (SD)	2.14 (0.953)	2.88 (0.976)	2.65 (1.02)	0.00474
Median [Min, Max]	2.00 [1.00, 5.00]	3.00 [1.00, 5.00]	3.00 [1.00, 5.00]	
t2_PEA_11				
Mean (SD)	1.72 (0.960)	2.54 (1.30)	2.29 (1.26)	0.0139
Median [Min, Max]	1.00 [1.00, 4.00]	2.00 [1.00, 5.00]	2.00 [1.00, 5.00]	
t2_PEA_12				
Mean (SD)	1.48 (0.829)	2.26 (1.19)	2.02 (1.15)	0.00882
Median [Min, Max]	1.00 [1.00, 4.00]	2.00 [1.00, 5.00]	2.00 [1.00, 5.00]	
t2_PEA_13				
Mean (SD)	2.59 (1.43)	3.74 (1.42)	3.38 (1.51)	0.0025
Median [Min, Max]	3.00 [1.00, 5.00]	4.00 [1.00, 5.00]	4.00 [1.00, 5.00]	
t2_PEA_14				
Mean (SD)	2.41 (1.32)	3.25 (1.33)	2.99 (1.38)	0.0246
Median [Min, Max]	2.00 [1.00, 5.00]	3.00 [1.00, 5.00]	3.00 [1.00, 5.00]	
t2_PEA_15				
Mean (SD)	1.41 (0.946)	2.29 (1.45)	2.02 (1.38)	0.0156
Median [Min, Max]	1.00 [1.00, 5.00]	2.00 [1.00, 5.00]	1.00 [1.00, 5.00]	
t2_PEA_16				
Mean (SD)	2.31 (1.34)	3.20 (1.28)	2.93 (1.35)	0.0122
Median [Min, Max]	2.00 [1.00, 5.00]	3.00 [1.00, 5.00]	3.00 [1.00, 5.00]	
t2_PEA_17				
Mean (SD)	1.48 (1.02)	2.92 (1.27)	2.48 (1.37)	<0.001
Median [Min, Max]	1.00 [1.00, 5.00]	3.00 [1.00, 5.00]	2.00 [1.00, 5.00]	
t2_PEA_18				

	Control (N=29)	Intervention (N=65)	Overall (N=94)	P-value
Mean (SD)	1.72 (0.455)	1.74 (0.443)	1.73 (0.444)	0.99
Median [Min, Max]	2.00 [1.00, 2.00]	2.00 [1.00, 2.00]	2.00 [1.00, 2.00]	
t2_PEA_19				
Mean (SD)	1.93 (0.258)	1.86 (0.348)	1.88 (0.323)	0.63
Median [Min, Max]	2.00 [1.00, 2.00]	2.00 [1.00, 2.00]	2.00 [1.00, 2.00]	
t2_PEA_20				
Mean (SD)	1.79 (0.412)	1.63 (0.486)	1.68 (0.469)	0.3
Median [Min, Max]	2.00 [1.00, 2.00]	2.00 [1.00, 2.00]	2.00 [1.00, 2.00]	
t2_PEA_21				
Mean (SD)	1.90 (0.310)	1.85 (0.364)	1.86 (0.347)	0.81
Median [Min, Max]	2.00 [1.00, 2.00]	2.00 [1.00, 2.00]	2.00 [1.00, 2.00]	
t2_PEA_22				
Mean (SD)	1.52 (0.509)	1.51 (0.504)	1.51 (0.503)	0.996
Median [Min, Max]	2.00 [1.00, 2.00]	2.00 [1.00, 2.00]	2.00 [1.00, 2.00]	
t2_peb_sum				
Mean (SD)	46.8 (9.56)	56.7 (10.8)	53.5 (11.4)	<0.001
Median [Min, Max]	45.0 [25.0, 69.0]	56.0 [30.0, 84.0]	54.0 [25.0, 84.0]	
Missing	0 (0%)	3 (4.6%)	3 (3.2%)	
t2_peb_transport				
Mean (SD)	15.2 (3.67)	15.8 (3.33)	15.6 (3.43)	0.727
Median [Min, Max]	16.0 [6.00, 20.0]	16.0 [5.00, 20.0]	16.0 [5.00, 20.0]	
t2_peb_food				
Mean (SD)	8.76 (2.64)	10.3 (3.14)	9.78 (3.06)	0.0911
Median [Min, Max]	9.00 [5.00, 14.0]	11.0 [3.00, 15.0]	10.0 [3.00, 15.0]	
Missing	0 (0%)	2 (3.1%)	2 (2.1%)	
t2_peb_waste				
Mean (SD)	7.28 (1.49)	7.75 (1.21)	7.60 (1.31)	0.271
Median [Min, Max]	7.00 [3.00, 10.0]	8.00 [6.00, 10.0]	8.00 [3.00, 10.0]	
Missing	0 (0%)	1 (1.5%)	1 (1.1%)	
t2_peb_adapt				

	Control (N=29)	Intervention (N=65)	Overall (N=94)	P-value
Mean (SD)	2.14 (0.953)	2.88 (0.976)	2.65 (1.02)	0.00474
Median [Min, Max]	2.00 [1.00, 5.00]	3.00 [1.00, 5.00]	3.00 [1.00, 5.00]	
t2_peb_agency				
Mean (SD)	13.4 (6.02)	20.2 (6.32)	18.1 (6.95)	<0.001
Median [Min, Max]	12.0 [7.00, 32.0]	20.0 [8.00, 35.0]	18.0 [7.00, 35.0]	
t1_Q11_leader				
Mean (SD)	NA (NA)	3.48 (0.903)	3.48 (0.903)	1
Median [Min, Max]	NA [NA, NA]	3.00 [1.00, 5.00]	3.00 [1.00, 5.00]	
Missing	29 (100%)	0 (0%)	29 (30.9%)	
t1_Q11_manager				
Mean (SD)	NA (NA)	3.28 (1.12)	3.28 (1.12)	1
Median [Min, Max]	NA [NA, NA]	3.00 [1.00, 5.00]	3.00 [1.00, 5.00]	
Missing	29 (100%)	1 (1.5%)	30 (31.9%)	
t1_Q11_peers				
Mean (SD)	NA (NA)	3.28 (0.826)	3.28 (0.826)	1
Median [Min, Max]	NA [NA, NA]	3.00 [1.00, 5.00]	3.00 [1.00, 5.00]	
Missing	29 (100%)	1 (1.5%)	30 (31.9%)	
t1_Q11_staff				
Mean (SD)	NA (NA)	3.43 (1.01)	3.43 (1.01)	1
Median [Min, Max]	NA [NA, NA]	3.00 [1.00, 5.00]	3.00 [1.00, 5.00]	
Missing	29 (100%)	5 (7.7%)	34 (36.2%)	
t1_Q11_you				
Mean (SD)	3.17 (0.928)	4.00 (0.810)	3.74 (0.927)	<0.001
Median [Min, Max]	3.00 [1.00, 5.00]	4.00 [2.00, 5.00]	4.00 [1.00, 5.00]	
t1_org_standup				
Mean (SD)	NA (NA)	17.6 (3.48)	17.6 (3.48)	1
Median [Min, Max]	NA [NA, NA]	17.0 [9.00, 25.0]	17.0 [9.00, 25.0]	
Missing	29 (100%)	5 (7.7%)	34 (36.2%)	
t1_Q12_projects				
Mean (SD)	2.66 (1.52)	3.42 (1.10)	3.18 (1.29)	0.0287

	Control (N=29)	Intervention (N=65)	Overall (N=94)	P-value
Median [Min, Max]	3.00 [1.00, 6.00]	3.00 [1.00, 5.00]	3.00 [1.00, 6.00]	
t1_Q12_department				
Mean (SD)	2.66 (1.37)	3.14 (0.990)	2.99 (1.14)	0.161
Median [Min, Max]	3.00 [1.00, 6.00]	3.00 [1.00, 5.00]	3.00 [1.00, 6.00]	
Missing	0 (0%)	1 (1.5%)	1 (1.1%)	
t1_Q12_visions				
Mean (SD)	2.79 (1.35)	3.31 (1.07)	3.15 (1.18)	0.143
Median [Min, Max]	3.00 [1.00, 6.00]	3.00 [1.00, 5.00]	3.00 [1.00, 6.00]	
Missing	0 (0%)	1 (1.5%)	1 (1.1%)	
t1_Q12_regulations				
Mean (SD)	2.66 (1.08)	3.00 (1.04)	2.89 (1.06)	0.346
Median [Min, Max]	3.00 [1.00, 5.00]	3.00 [1.00, 5.00]	3.00 [1.00, 5.00]	
Missing	0 (0%)	1 (1.5%)	1 (1.1%)	
t1_Q12_planning				
Mean (SD)	2.24 (1.33)	2.72 (0.983)	2.57 (1.12)	0.16
Median [Min, Max]	2.00 [1.00, 6.00]	3.00 [1.00, 5.00]	3.00 [1.00, 6.00]	
Missing	0 (0%)	1 (1.5%)	1 (1.1%)	
t1_Q12_training				
Mean (SD)	2.31 (1.04)	2.88 (1.02)	2.70 (1.05)	0.0544
Median [Min, Max]	2.00 [1.00, 4.00]	3.00 [1.00, 5.00]	3.00 [1.00, 5.00]	
Missing	0 (0%)	1 (1.5%)	1 (1.1%)	
t1_Q12_structure				
Mean (SD)	2.62 (1.45)	2.84 (0.946)	2.77 (1.12)	0.676
Median [Min, Max]	3.00 [1.00, 6.00]	3.00 [1.00, 5.00]	3.00 [1.00, 6.00]	
Missing	0 (0%)	1 (1.5%)	1 (1.1%)	
t1_Q12_cooperation				
Mean (SD)	3.10 (1.82)	2.98 (1.05)	3.02 (1.33)	0.924
Median [Min, Max]	3.00 [1.00, 6.00]	3.00 [1.00, 5.00]	3.00 [1.00, 6.00]	
Missing	0 (0%)	2 (3.1%)	2 (2.1%)	
t1_Q12_budget				
Mean (SD)	3.41 (1.96)	2.77 (0.913)	2.98 (1.36)	0.11

	Control (N=29)	Intervention (N=65)	Overall (N=94)	P-value
Median [Min, Max]	3.00 [1.00, 6.00]	3.00 [1.00, 5.00]	3.00 [1.00, 6.00]	
Missing	0 (0%)	3 (4.6%)	3 (3.2%)	
t1_Q12_hr				
Mean (SD)	3.34 (2.04)	2.63 (0.938)	2.86 (1.41)	0.0793
Median [Min, Max]	3.00 [1.00, 6.00]	3.00 [1.00, 5.00]	3.00 [1.00, 6.00]	
Missing	0 (0%)	2 (3.1%)	2 (2.1%)	
t1_org_integrated				
Mean (SD)	27.8 (10.9)	29.5 (8.04)	29.0 (9.04)	0.698
Median [Min, Max]	28.0 [10.0, 51.0]	28.0 [13.0, 50.0]	28.0 [10.0, 51.0]	
Missing	0 (0%)	4 (6.2%)	4 (4.3%)	
t1_org_integrate_future				
Mean (SD)	2.66 (1.54)	4.08 (1.07)	3.64 (1.39)	<0.001
Median [Min, Max]	3.00 [1.00, 6.00]	4.00 [1.00, 5.00]	4.00 [1.00, 6.00]	
t2_Q11_you				
Mean (SD)	3.21 (1.08)	3.85 (0.870)	3.65 (0.981)	0.0131
Median [Min, Max]	3.00 [1.00, 5.00]	4.00 [2.00, 5.00]	4.00 [1.00, 5.00]	
t2_Q12_projects				
Mean (SD)	2.41 (1.18)	3.42 (1.01)	3.11 (1.16)	<0.001
Median [Min, Max]	2.00 [1.00, 5.00]	3.00 [1.00, 5.00]	3.00 [1.00, 5.00]	
t2_Q12_department				
Mean (SD)	2.72 (1.19)	3.42 (1.03)	3.20 (1.12)	0.021
Median [Min, Max]	3.00 [1.00, 5.00]	4.00 [2.00, 5.00]	3.00 [1.00, 5.00]	
t2_Q12_visions				
Mean (SD)	2.69 (1.11)	3.42 (1.17)	3.19 (1.19)	0.0217
Median [Min, Max]	3.00 [1.00, 5.00]	3.50 [1.00, 5.00]	3.00 [1.00, 5.00]	
Missing	0 (0%)	1 (1.5%)	1 (1.1%)	
t2_Q12_regulations				
Mean (SD)	2.62 (1.01)	3.11 (0.986)	2.96 (1.02)	0.0978
Median [Min, Max]	3.00 [1.00, 5.00]	3.00 [1.00, 5.00]	3.00 [1.00, 5.00]	
t2_Q12_planning				

	Control (N=29)	Intervention (N=65)	Overall (N=94)	P-value
Mean (SD)	2.24 (1.09)	2.85 (1.20)	2.66 (1.20)	0.0755
Median [Min, Max]	2.00 [1.00, 4.00]	3.00 [1.00, 5.00]	3.00 [1.00, 5.00]	
t2_Q12_training				
Mean (SD)	2.45 (1.09)	3.22 (1.08)	2.98 (1.14)	0.00942
Median [Min, Max]	2.00 [1.00, 4.00]	3.00 [1.00, 5.00]	3.00 [1.00, 5.00]	
t2_Q12_structure				
Mean (SD)	2.28 (1.10)	3.09 (1.04)	2.84 (1.12)	0.00425
Median [Min, Max]	2.00 [1.00, 4.00]	3.00 [1.00, 5.00]	3.00 [1.00, 5.00]	
t2_Q12_cooperation				
Mean (SD)	2.21 (1.08)	3.06 (1.24)	2.80 (1.25)	0.00832
Median [Min, Max]	2.00 [1.00, 4.00]	3.00 [1.00, 5.00]	3.00 [1.00, 5.00]	
t2_Q12_budget				
Mean (SD)	2.10 (1.05)	2.89 (1.13)	2.65 (1.16)	0.00891
Median [Min, Max]	2.00 [1.00, 5.00]	3.00 [1.00, 5.00]	2.50 [1.00, 5.00]	
t2_Q12_hr				
Mean (SD)	2.10 (1.01)	2.91 (1.05)	2.66 (1.10)	0.00425
Median [Min, Max]	2.00 [1.00, 4.00]	3.00 [1.00, 5.00]	2.00 [1.00, 5.00]	
Missing	0 (0%)	1 (1.5%)	1 (1.1%)	
t2_org_integrated				
Mean (SD)	23.8 (9.35)	31.5 (9.27)	29.1 (9.91)	0.00234
Median [Min, Max]	22.0 [10.0, 42.0]	32.0 [15.0, 50.0]	29.0 [10.0, 50.0]	
Missing	0 (0%)	2 (3.1%)	2 (2.1%)	
t2_org_integrate_future				
Mean (SD)	3.00 (1.39)	4.23 (1.06)	3.85 (1.29)	<0.001
Median [Min, Max]	3.00 [1.00, 5.00]	5.00 [1.00, 5.00]	4.00 [1.00, 5.00]	
t1_Q12_projects_inf				

	Control (N=29)	Intervention (N=65)	Overall (N=94)	P-value
Mean (SD)	3.17 (0.889)	3.89 (0.935)	3.66 (0.975)	0.00412
Median [Min, Max]	3.00 [1.00, 5.00]	4.00 [2.00, 5.00]	4.00 [1.00, 5.00]	
Missing	0 (0%)	2 (3.1%)	2 (2.1%)	
t1_Q12_department_inf				
Mean (SD)	2.69 (1.04)	3.48 (0.998)	3.23 (1.07)	0.00409
Median [Min, Max]	3.00 [1.00, 5.00]	4.00 [1.00, 5.00]	3.00 [1.00, 5.00]	
Missing	0 (0%)	2 (3.1%)	2 (2.1%)	
t1_Q12_visions_inf				
Mean (SD)	2.28 (0.882)	3.63 (0.979)	3.20 (1.14)	<0.001
Median [Min, Max]	2.00 [1.00, 4.00]	4.00 [1.00, 5.00]	3.00 [1.00, 5.00]	
Missing	0 (0%)	3 (4.6%)	3 (3.2%)	
t1_Q12_regulations_inf				
Mean (SD)	2.21 (0.940)	3.29 (1.09)	2.95 (1.16)	<0.001
Median [Min, Max]	2.00 [1.00, 4.00]	3.00 [1.00, 5.00]	3.00 [1.00, 5.00]	
Missing	0 (0%)	3 (4.6%)	3 (3.2%)	
t1_Q12_planning_inf				
Mean (SD)	2.62 (1.15)	3.24 (1.21)	3.04 (1.22)	0.0752
Median [Min, Max]	3.00 [1.00, 5.00]	3.00 [1.00, 5.00]	3.00 [1.00, 5.00]	
Missing	0 (0%)	3 (4.6%)	3 (3.2%)	
t1_Q12_training_inf				
Mean (SD)	2.52 (1.12)	3.31 (1.20)	3.05 (1.22)	0.0152
Median [Min, Max]	3.00 [1.00, 4.00]	3.00 [1.00, 5.00]	3.00 [1.00, 5.00]	
Missing	0 (0%)	3 (4.6%)	3 (3.2%)	
t1_Q12_structure_inf				
Mean (SD)	2.38 (1.01)	3.03 (1.17)	2.82 (1.16)	0.0422
Median [Min, Max]	2.00 [1.00, 4.00]	3.00 [1.00, 5.00]	3.00 [1.00, 5.00]	
Missing	0 (0%)	3 (4.6%)	3 (3.2%)	
t1_Q12_cooperation_inf				

	Control (N=29)	Intervention (N=65)	Overall (N=94)	P-value
Mean (SD)	1.76 (0.739)	3.11 (1.25)	2.68 (1.28)	<0.001
Median [Min, Max]	2.00 [1.00, 3.00]	3.00 [1.00, 5.00]	3.00 [1.00, 5.00]	
Missing	0 (0%)	4 (6.2%)	4 (4.3%)	
t1_Q12_budget_inf				
Mean (SD)	1.41 (0.568)	2.48 (1.14)	2.14 (1.11)	<0.001
Median [Min, Max]	1.00 [1.00, 3.00]	2.50 [1.00, 5.00]	2.00 [1.00, 5.00]	
Missing	0 (0%)	3 (4.6%)	3 (3.2%)	
t1_Q12_hr_inf				
Mean (SD)	1.41 (0.628)	2.47 (1.17)	2.14 (1.14)	<0.001
Median [Min, Max]	1.00 [1.00, 3.00]	2.50 [1.00, 5.00]	2.00 [1.00, 5.00]	
Missing	0 (0%)	1 (1.5%)	1 (1.1%)	
t1_influence_sum				
Mean (SD)	22.4 (6.14)	31.9 (9.07)	28.8 (9.33)	<0.001
Median [Min, Max]	22.0 [10.0, 35.0]	32.0 [11.0, 50.0]	28.0 [10.0, 50.0]	
Missing	0 (0%)	5 (7.7%)	5 (5.3%)	
experience_max				
Mean (SD)	0.667 (0.784)	1.24 (1.37)	1.04 (1.22)	0.146
Median [Min, Max]	1.00 [0, 3.00]	1.00 [0, 4.00]	1.00 [0, 4.00]	
Missing	2 (6.9%)	14 (21.5%)	16 (17.0%)	
t1_NJ_1r				
Mean (SD)	NA (NA)	4.00 (1.00)	4.00 (1.00)	1
Median [Min, Max]	NA [NA, NA]	4.00 [1.00, 5.00]	4.00 [1.00, 5.00]	
Missing	29 (100%)	0 (0%)	29 (30.9%)	
t1_NJ_2r				
Mean (SD)	NA (NA)	3.80 (1.09)	3.80 (1.09)	1
Median [Min, Max]	NA [NA, NA]	4.00 [1.00, 5.00]	4.00 [1.00, 5.00]	
Missing	29 (100%)	0 (0%)	29 (30.9%)	
t1_NJ_3r				
Mean (SD)	NA (NA)	3.82 (1.06)	3.82 (1.06)	1
Median [Min, Max]	NA [NA, NA]	4.00 [2.00, 5.00]	4.00 [2.00, 5.00]	
Missing	29 (100%)	0 (0%)	29 (30.9%)	
t1_NR_1				

	Control (N=29)	Intervention (N=65)	Overall (N=94)	P-value
Mean (SD)	NA (NA)	3.45 (0.919)	3.45 (0.919)	1
Median [Min, Max]	NA [NA, NA]	4.00 [1.00, 5.00]	4.00 [1.00, 5.00]	
Missing	29 (100%)	0 (0%)	29 (30.9%)	
t1_NR_2				
Mean (SD)	NA (NA)	3.05 (1.01)	3.05 (1.01)	1
Median [Min, Max]	NA [NA, NA]	3.00 [1.00, 5.00]	3.00 [1.00, 5.00]	
Missing	29 (100%)	0 (0%)	29 (30.9%)	
t1_AA_1				
Mean (SD)	NA (NA)	3.52 (1.13)	3.52 (1.13)	1
Median [Min, Max]	NA [NA, NA]	4.00 [1.00, 5.00]	4.00 [1.00, 5.00]	
Missing	29 (100%)	0 (0%)	29 (30.9%)	
t1_AA_2r				
Mean (SD)	NA (NA)	3.14 (0.899)	3.14 (0.899)	1
Median [Min, Max]	NA [NA, NA]	3.00 [1.00, 5.00]	3.00 [1.00, 5.00]	
Missing	29 (100%)	0 (0%)	29 (30.9%)	
t1_AA_3r				
Mean (SD)	NA (NA)	3.14 (0.899)	3.14 (0.899)	1
Median [Min, Max]	NA [NA, NA]	3.00 [1.00, 5.00]	3.00 [1.00, 5.00]	
Missing	29 (100%)	0 (0%)	29 (30.9%)	
t1_ffmq_sum				
Mean (SD)	NA (NA)	27.2 (3.99)	27.2 (3.99)	1
Median [Min, Max]	NA [NA, NA]	27.0 [16.0, 36.0]	27.0 [16.0, 36.0]	
Missing	29 (100%)	0 (0%)	29 (30.9%)	
t1_ffmq_nj				
Mean (SD)	NA (NA)	6.38 (2.87)	6.38 (2.87)	1
Median [Min, Max]	NA [NA, NA]	6.00 [3.00, 14.0]	6.00 [3.00, 14.0]	
Missing	29 (100%)	0 (0%)	29 (30.9%)	
t1_ffmq_nr				
Mean (SD)	NA (NA)	6.49 (1.67)	6.49 (1.67)	1
Median [Min, Max]	NA [NA, NA]	7.00 [3.00, 10.0]	7.00 [3.00, 10.0]	
Missing	29 (100%)	0 (0%)	29 (30.9%)	

	Control (N=29)	Intervention (N=65)	Overall (N=94)	P-value
t1_ffmq_aa				
Mean (SD)	NA (NA)	6.28 (1.80)	6.28 (1.80)	1
Median [Min, Max]	NA [NA, NA]	6.00 [2.00, 10.0]	6.00 [2.00, 10.0]	
Missing	29 (100%)	0 (0%)	29 (30.9%)	
t1_ffmq_obs				
Mean (SD)	NA (NA)	3.52 (1.13)	3.52 (1.13)	1
Median [Min, Max]	NA [NA, NA]	4.00 [1.00, 5.00]	4.00 [1.00, 5.00]	
Missing	29 (100%)	0 (0%)	29 (30.9%)	
t2_NJ_1r				
Mean (SD)	NA (NA)	4.20 (0.905)	4.20 (0.905)	1
Median [Min, Max]	NA [NA, NA]	4.00 [2.00, 5.00]	4.00 [2.00, 5.00]	
Missing	29 (100%)	0 (0%)	29 (30.9%)	
t2_NJ_2r				
Mean (SD)	NA (NA)	4.29 (0.897)	4.29 (0.897)	1
Median [Min, Max]	NA [NA, NA]	5.00 [2.00, 5.00]	5.00 [2.00, 5.00]	
Missing	29 (100%)	0 (0%)	29 (30.9%)	
t2_NJ_3r				
Mean (SD)	NA (NA)	4.12 (0.944)	4.12 (0.944)	1
Median [Min, Max]	NA [NA, NA]	4.00 [2.00, 5.00]	4.00 [2.00, 5.00]	
Missing	29 (100%)	0 (0%)	29 (30.9%)	
t2_NR_1				
Mean (SD)	NA (NA)	3.50 (0.926)	3.50 (0.926)	1
Median [Min, Max]	NA [NA, NA]	4.00 [1.00, 5.00]	4.00 [1.00, 5.00]	
Missing	29 (100%)	1 (1.5%)	30 (31.9%)	
t2_NR_2				
Mean (SD)	NA (NA)	3.22 (0.927)	3.22 (0.927)	1
Median [Min, Max]	NA [NA, NA]	3.00 [1.00, 5.00]	3.00 [1.00, 5.00]	
Missing	29 (100%)	0 (0%)	29 (30.9%)	
t2_AA_1				
Mean (SD)	NA (NA)	3.45 (1.16)	3.45 (1.16)	1
Median [Min, Max]	NA [NA, NA]	4.00 [1.00, 5.00]	4.00 [1.00, 5.00]	

	Control (N=29)	Intervention (N=65)	Overall (N=94)	P-value
Missing	29 (100%)	0 (0%)	29 (30.9%)	
t2_AA_2r				
Mean (SD)	NA (NA)	3.29 (0.879)	3.29 (0.879)	1
Median [Min, Max]	NA [NA, NA]	3.00 [1.00, 5.00]	3.00 [1.00, 5.00]	
Missing	29 (100%)	0 (0%)	29 (30.9%)	
t2_AA_3r				
Mean (SD)	NA (NA)	3.29 (0.879)	3.29 (0.879)	1
Median [Min, Max]	NA [NA, NA]	3.00 [1.00, 5.00]	3.00 [1.00, 5.00]	
Missing	29 (100%)	0 (0%)	29 (30.9%)	
t2_ffmq_sum				
Mean (SD)	NA (NA)	29.5 (4.53)	29.5 (4.53)	1
Median [Min, Max]	NA [NA, NA]	29.0 [12.0, 40.0]	29.0 [12.0, 40.0]	
Missing	29 (100%)	1 (1.5%)	30 (31.9%)	
t2_ffmq_nj				
Mean (SD)	NA (NA)	5.38 (2.45)	5.38 (2.45)	1
Median [Min, Max]	NA [NA, NA]	5.00 [3.00, 12.0]	5.00 [3.00, 12.0]	
Missing	29 (100%)	0 (0%)	29 (30.9%)	
t2_ffmq_nr				
Mean (SD)	NA (NA)	6.73 (1.59)	6.73 (1.59)	1
Median [Min, Max]	NA [NA, NA]	7.00 [2.00, 10.0]	7.00 [2.00, 10.0]	
Missing	29 (100%)	1 (1.5%)	30 (31.9%)	
t2_ffmq_aa				
Mean (SD)	NA (NA)	6.58 (1.76)	6.58 (1.76)	1
Median [Min, Max]	NA [NA, NA]	6.00 [2.00, 10.0]	6.00 [2.00, 10.0]	
Missing	29 (100%)	0 (0%)	29 (30.9%)	
t2_ffmq_obs				
Mean (SD)	NA (NA)	3.45 (1.16)	3.45 (1.16)	1
Median [Min, Max]	NA [NA, NA]	4.00 [1.00, 5.00]	4.00 [1.00, 5.00]	
Missing	29 (100%)	0 (0%)	29 (30.9%)	
t1_MHC_1				
Mean (SD)	2.72 (1.22)	3.68 (0.831)	3.38 (1.06)	<0.001

	Control (N=29)	Intervention (N=65)	Overall (N=94)	P-value
Median [Min, Max]	3.00 [0, 5.00]	4.00 [2.00, 5.00]	4.00 [0, 5.00]	
t1_MHC_3				
Mean (SD)	2.72 (1.28)	3.65 (1.07)	3.36 (1.21)	0.00248
Median [Min, Max]	3.00 [0, 5.00]	4.00 [1.00, 5.00]	4.00 [0, 5.00]	
t1_MHC_4				
Mean (SD)	1.90 (1.32)	2.92 (1.36)	2.61 (1.42)	0.00481
Median [Min, Max]	1.00 [0, 4.00]	3.00 [0, 5.00]	3.00 [0, 5.00]	
t1_MHC_5				
Mean (SD)	2.62 (1.68)	2.97 (1.57)	2.86 (1.60)	0.624
Median [Min, Max]	3.00 [0, 5.00]	3.00 [0, 5.00]	3.00 [0, 5.00]	
t1_MHC_11				
Mean (SD)	3.48 (1.30)	3.83 (1.01)	3.72 (1.11)	0.374
Median [Min, Max]	4.00 [1.00, 5.00]	4.00 [1.00, 5.00]	4.00 [1.00, 5.00]	
t1_MHC_14				
Mean (SD)	2.62 (1.72)	3.55 (1.37)	3.27 (1.54)	0.0238
Median [Min, Max]	3.00 [0, 5.00]	4.00 [0, 5.00]	4.00 [0, 5.00]	
t1_mhc_sum				
Mean (SD)	16.1 (6.88)	20.6 (5.49)	19.2 (6.27)	0.00474
Median [Min, Max]	17.0 [4.00, 27.0]	21.0 [8.00, 30.0]	20.0 [4.00, 30.0]	
t1_mhc_swb				
Mean (SD)	4.52 (2.69)	5.89 (2.41)	5.47 (2.57)	0.0548
Median [Min, Max]	4.00 [0, 9.00]	6.00 [1.00, 10.0]	5.50 [0, 10.0]	
t1_mhc_ewb				
Mean (SD)	5.45 (2.29)	7.32 (1.79)	6.74 (2.13)	<0.001
Median [Min, Max]	5.00 [1.00, 10.0]	8.00 [3.00, 10.0]	7.00 [1.00, 10.0]	
t1_mhc_pwb				
Mean (SD)	6.10 (2.79)	7.38 (2.07)	6.99 (2.38)	0.0531
Median [Min, Max]	6.00 [1.00, 10.0]	8.00 [2.00, 10.0]	8.00 [1.00, 10.0]	
t2_MHC_1				
Mean (SD)	2.93 (1.22)	3.78 (0.838)	3.52 (1.04)	<0.001

	Control (N=29)	Intervention (N=65)	Overall (N=94)	P-value
Median [Min, Max]	3.00 [0, 5.00]	4.00 [2.00, 5.00]	4.00 [0, 5.00]	
t2_MHC_3				
Mean (SD)	2.97 (1.32)	3.74 (1.02)	3.50 (1.17)	0.0117
Median [Min, Max]	3.00 [1.00, 5.00]	4.00 [1.00, 5.00]	4.00 [1.00, 5.00]	
t2_MHC_4				
Mean (SD)	2.00 (1.25)	3.28 (1.27)	2.88 (1.39)	<0.001
Median [Min, Max]	2.00 [0, 4.00]	3.00 [0, 5.00]	3.00 [0, 5.00]	
t2_MHC_5				
Mean (SD)	2.90 (1.42)	3.14 (1.46)	3.06 (1.44)	0.755
Median [Min, Max]	3.00 [0, 5.00]	3.00 [0, 5.00]	3.00 [0, 5.00]	
t2_MHC_11				
Mean (SD)	3.52 (1.33)	4.02 (0.944)	3.86 (1.09)	0.123
Median [Min, Max]	4.00 [1.00, 5.00]	4.00 [1.00, 5.00]	4.00 [1.00, 5.00]	
t2_MHC_14				
Mean (SD)	2.86 (1.57)	3.61 (1.35)	3.38 (1.46)	0.0712
Median [Min, Max]	3.00 [0, 5.00]	4.00 [0, 5.00]	4.00 [0, 5.00]	
Missing	0 (0%)	1 (1.5%)	1 (1.1%)	
t2_mhc_sum				
Mean (SD)	17.2 (6.95)	21.6 (5.52)	20.2 (6.31)	0.00666
Median [Min, Max]	18.0 [4.00, 28.0]	22.0 [7.00, 30.0]	21.0 [4.00, 30.0]	
Missing	0 (0%)	1 (1.5%)	1 (1.1%)	
t2_mhc_swb				
Mean (SD)	4.90 (2.40)	6.42 (2.43)	5.95 (2.51)	0.0239
Median [Min, Max]	5.00 [0, 9.00]	7.00 [1.00, 10.0]	6.00 [0, 10.0]	
t2_mhc_ewb				
Mean (SD)	5.90 (2.44)	7.52 (1.77)	7.02 (2.13)	0.00239
Median [Min, Max]	7.00 [1.00, 10.0]	8.00 [3.00, 10.0]	7.50 [1.00, 10.0]	
t2_mhc_pwb				
Mean (SD)	6.38 (2.69)	7.64 (2.07)	7.25 (2.34)	0.0539
Median [Min, Max]	6.00 [1.00, 10.0]	8.00 [2.00, 10.0]	8.00 [1.00, 10.0]	

	Control (N=29)	Intervention (N=65)	Overall (N=94)	P-value
Missing	0 (0%)	1 (1.5%)	1 (1.1%)	
t1_Other_Comp_1				
Mean (SD)	NA (NA)	3.78 (0.893)	3.78 (0.893)	1
Median [Min, Max]	NA [NA, NA]	4.00 [2.00, 5.00]	4.00 [2.00, 5.00]	
Missing	29 (100%)	0 (0%)	29 (30.9%)	
t1_Other_Comp_2				
Mean (SD)	NA (NA)	3.60 (0.898)	3.60 (0.898)	1
Median [Min, Max]	NA [NA, NA]	4.00 [1.00, 5.00]	4.00 [1.00, 5.00]	
Missing	29 (100%)	0 (0%)	29 (30.9%)	
t1_Other_Comp_3				
Mean (SD)	NA (NA)	3.98 (0.718)	3.98 (0.718)	1
Median [Min, Max]	NA [NA, NA]	4.00 [2.00, 5.00]	4.00 [2.00, 5.00]	
Missing	29 (100%)	0 (0%)	29 (30.9%)	
t1_Other_Comp_4				
Mean (SD)	NA (NA)	3.97 (0.749)	3.97 (0.749)	1
Median [Min, Max]	NA [NA, NA]	4.00 [2.00, 5.00]	4.00 [2.00, 5.00]	
Missing	29 (100%)	0 (0%)	29 (30.9%)	
t1_Other_Comp_5				
Mean (SD)	NA (NA)	4.06 (0.768)	4.06 (0.768)	1
Median [Min, Max]	NA [NA, NA]	4.00 [2.00, 5.00]	4.00 [2.00, 5.00]	
Missing	29 (100%)	0 (0%)	29 (30.9%)	
t1_comp_sum				
Mean (SD)	NA (NA)	19.4 (2.89)	19.4 (2.89)	1
Median [Min, Max]	NA [NA, NA]	19.0 [13.0, 25.0]	19.0 [13.0, 25.0]	
Missing	29 (100%)	0 (0%)	29 (30.9%)	
t1_comp_engage				
Mean (SD)	NA (NA)	7.38 (1.49)	7.38 (1.49)	1
Median [Min, Max]	NA [NA, NA]	7.00 [4.00, 10.0]	7.00 [4.00, 10.0]	
Missing	29 (100%)	0 (0%)	29 (30.9%)	
t1_comp_act				
Mean (SD)	NA (NA)	12.0 (1.83)	12.0 (1.83)	1

	Control (N=29)	Intervention (N=65)	Overall (N=94)	P-value
Median [Min, Max]	NA [NA, NA]	12.0 [8.00, 15.0]	12.0 [8.00, 15.0]	
Missing	29 (100%)	0 (0%)	29 (30.9%)	
t2_Other_Comp_1				
Mean (SD)	NA (NA)	3.55 (1.02)	3.55 (1.02)	1
Median [Min, Max]	NA [NA, NA]	4.00 [1.00, 5.00]	4.00 [1.00, 5.00]	
Missing	29 (100%)	0 (0%)	29 (30.9%)	
t2_Other_Comp_2				
Mean (SD)	NA (NA)	3.77 (0.806)	3.77 (0.806)	1
Median [Min, Max]	NA [NA, NA]	4.00 [2.00, 5.00]	4.00 [2.00, 5.00]	
Missing	29 (100%)	0 (0%)	29 (30.9%)	
t2_Other_Comp_3				
Mean (SD)	NA (NA)	4.05 (0.672)	4.05 (0.672)	1
Median [Min, Max]	NA [NA, NA]	4.00 [2.00, 5.00]	4.00 [2.00, 5.00]	
Missing	29 (100%)	0 (0%)	29 (30.9%)	
t2_Other_Comp_4				
Mean (SD)	NA (NA)	4.03 (0.770)	4.03 (0.770)	1
Median [Min, Max]	NA [NA, NA]	4.00 [2.00, 5.00]	4.00 [2.00, 5.00]	
Missing	29 (100%)	0 (0%)	29 (30.9%)	
t2_Other_Comp_5				
Mean (SD)	NA (NA)	4.16 (0.739)	4.16 (0.739)	1
Median [Min, Max]	NA [NA, NA]	4.00 [2.00, 5.00]	4.00 [2.00, 5.00]	
Missing	29 (100%)	1 (1.5%)	30 (31.9%)	
t2_comp_sum				
Mean (SD)	NA (NA)	19.6 (2.80)	19.6 (2.80)	1
Median [Min, Max]	NA [NA, NA]	20.0 [11.0, 25.0]	20.0 [11.0, 25.0]	
Missing	29 (100%)	1 (1.5%)	30 (31.9%)	
t2_comp_engage				
Mean (SD)	NA (NA)	7.32 (1.43)	7.32 (1.43)	1
Median [Min, Max]	NA [NA, NA]	8.00 [4.00, 10.0]	8.00 [4.00, 10.0]	
Missing	29 (100%)	0 (0%)	29 (30.9%)	
t2_comp_act				

	Control (N=29)	Intervention (N=65)	Overall (N=94)	P-value
Mean (SD)	NA (NA)	12.3 (1.80)	12.3 (1.80)	1
Median [Min, Max]	NA [NA, NA]	12.0 [7.00, 15.0]	12.0 [7.00, 15.0]	
Missing	29 (100%)	1 (1.5%)	30 (31.9%)	
t1_SCSS_Self_1				
Mean (SD)	NA (NA)	3.48 (0.903)	3.48 (0.903)	1
Median [Min, Max]	NA [NA, NA]	4.00 [1.00, 5.00]	4.00 [1.00, 5.00]	
Missing	29 (100%)	0 (0%)	29 (30.9%)	
t1_SCSS_Self_2				
Mean (SD)	NA (NA)	3.37 (1.08)	3.37 (1.08)	1
Median [Min, Max]	NA [NA, NA]	3.00 [1.00, 5.00]	3.00 [1.00, 5.00]	
Missing	29 (100%)	0 (0%)	29 (30.9%)	
t1_SCSS_Self_3				
Mean (SD)	NA (NA)	3.51 (1.12)	3.51 (1.12)	1
Median [Min, Max]	NA [NA, NA]	4.00 [1.00, 5.00]	4.00 [1.00, 5.00]	
Missing	29 (100%)	0 (0%)	29 (30.9%)	
t1_SCSS_Self_4				
Mean (SD)	NA (NA)	3.35 (1.05)	3.35 (1.05)	1
Median [Min, Max]	NA [NA, NA]	3.00 [1.00, 5.00]	3.00 [1.00, 5.00]	
Missing	29 (100%)	0 (0%)	29 (30.9%)	
t1_SCSS_Self_5				
Mean (SD)	NA (NA)	3.78 (0.739)	3.78 (0.739)	1
Median [Min, Max]	NA [NA, NA]	4.00 [2.00, 5.00]	4.00 [2.00, 5.00]	
Missing	29 (100%)	0 (0%)	29 (30.9%)	
t1_SCSS_Self_6				
Mean (SD)	NA (NA)	3.89 (1.08)	3.89 (1.08)	1
Median [Min, Max]	NA [NA, NA]	4.00 [1.00, 5.00]	4.00 [1.00, 5.00]	
Missing	29 (100%)	0 (0%)	29 (30.9%)	
t1_scs_sum				
Mean (SD)	NA (NA)	21.4 (3.96)	21.4 (3.96)	1
Median [Min, Max]	NA [NA, NA]	22.0 [10.0, 29.0]	22.0 [10.0, 29.0]	
Missing	29 (100%)	0 (0%)	29 (30.9%)	

	Control (N=29)	Intervention (N=65)	Overall (N=94)	P-value
t1_scs_selfkind				
Mean (SD)	NA (NA)	6.83 (1.71)	6.83 (1.71)	1
Median [Min, Max]	NA [NA, NA]	7.00 [2.00, 10.0]	7.00 [2.00, 10.0]	
Missing	29 (100%)	0 (0%)	29 (30.9%)	
t2_SCSS_Self_1				
Mean (SD)	NA (NA)	3.54 (0.937)	3.54 (0.937)	1
Median [Min, Max]	NA [NA, NA]	4.00 [1.00, 5.00]	4.00 [1.00, 5.00]	
Missing	29 (100%)	0 (0%)	29 (30.9%)	
t2_SCSS_Self_2				
Mean (SD)	NA (NA)	3.69 (0.934)	3.69 (0.934)	1
Median [Min, Max]	NA [NA, NA]	4.00 [2.00, 5.00]	4.00 [2.00, 5.00]	
Missing	29 (100%)	0 (0%)	29 (30.9%)	
t2_SCSS_Self_3				
Mean (SD)	NA (NA)	3.51 (0.954)	3.51 (0.954)	1
Median [Min, Max]	NA [NA, NA]	4.00 [1.00, 5.00]	4.00 [1.00, 5.00]	
Missing	29 (100%)	0 (0%)	29 (30.9%)	
t2_SCSS_Self_4				
Mean (SD)	NA (NA)	3.75 (0.926)	3.75 (0.926)	1
Median [Min, Max]	NA [NA, NA]	4.00 [1.00, 5.00]	4.00 [1.00, 5.00]	
Missing	29 (100%)	1 (1.5%)	30 (31.9%)	
t2_SCSS_Self_5				
Mean (SD)	NA (NA)	3.80 (0.642)	3.80 (0.642)	1
Median [Min, Max]	NA [NA, NA]	4.00 [2.00, 5.00]	4.00 [2.00, 5.00]	
Missing	29 (100%)	0 (0%)	29 (30.9%)	
t2_SCSS_Self_6				
Mean (SD)	NA (NA)	3.92 (1.11)	3.92 (1.11)	1
Median [Min, Max]	NA [NA, NA]	4.00 [1.00, 5.00]	4.00 [1.00, 5.00]	
Missing	29 (100%)	0 (0%)	29 (30.9%)	
t2_scs_sum				
Mean (SD)	NA (NA)	22.3 (3.55)	22.3 (3.55)	1
Median [Min, Max]	NA [NA, NA]	23.0 [10.0, 30.0]	23.0 [10.0, 30.0]	

	Control (N=29)	Intervention (N=65)	Overall (N=94)	P-value
Missing	29 (100%)	1 (1.5%)	30 (31.9%)	
t2_scs_selfkind				
Mean (SD)	NA (NA)	7.30 (1.57)	7.30 (1.57)	1
Median [Min, Max]	NA [NA, NA]	7.00 [2.00, 10.0]	7.00 [2.00, 10.0]	
Missing	29 (100%)	1 (1.5%)	30 (31.9%)	
t1_Interconnected				
Mean (SD)	NA (NA)	4.18 (1.96)	4.18 (1.96)	1
Median [Min, Max]	NA [NA, NA]	5.00 [1.00, 7.00]	5.00 [1.00, 7.00]	
Missing	29 (100%)	0 (0%)	29 (30.9%)	
t2_Interconnected				
Mean (SD)	4.14 (1.16)	5.20 (1.44)	4.87 (1.44)	0.00369
Median [Min, Max]	4.00 [3.00, 7.00]	5.00 [1.00, 7.00]	5.00 [1.00, 7.00]	
t1_Nature_Value				
Mean (SD)	NA (NA)	3.25 (0.811)	3.25 (0.811)	1
Median [Min, Max]	NA [NA, NA]	3.00 [1.00, 4.00]	3.00 [1.00, 4.00]	
Missing	29 (100%)	0 (0%)	29 (30.9%)	
t2_Nature_Value				
Mean (SD)	3.24 (0.951)	3.08 (0.835)	3.13 (0.870)	0.7
Median [Min, Max]	3.00 [1.00, 4.00]	3.00 [1.00, 4.00]	3.00 [1.00, 4.00]	
t1_Trust_gen				
Mean (SD)	NA (NA)	8.52 (1.26)	8.52 (1.26)	1
Median [Min, Max]	NA [NA, NA]	9.00 [5.00, 11.0]	9.00 [5.00, 11.0]	
Missing	29 (100%)	0 (0%)	29 (30.9%)	
t2_Trust_gen				
Mean (SD)	7.93 (1.67)	8.38 (1.44)	8.24 (1.52)	0.411
Median [Min, Max]	8.00 [4.00, 10.0]	9.00 [3.00, 11.0]	8.00 [3.00, 11.0]	
t1_Trust_1				
Mean (SD)	NA (NA)	2.48 (1.16)	2.48 (1.16)	1
Median [Min, Max]	NA [NA, NA]	3.00 [1.00, 4.00]	3.00 [1.00, 4.00]	
Missing	29 (100%)	0 (0%)	29 (30.9%)	

	Control (N=29)	Intervention (N=65)	Overall (N=94)	P-value
t1_Trust_2				
Mean (SD)	2.59 (0.867)	3.20 (1.05)	3.01 (1.03)	0.0274
Median [Min, Max]	2.00 [1.00, 4.00]	4.00 [1.00, 4.00]	3.00 [1.00, 4.00]	
t2_Trust_1				
Mean (SD)	2.31 (1.26)	2.46 (1.15)	2.41 (1.18)	0.848
Median [Min, Max]	3.00 [1.00, 4.00]	3.00 [1.00, 4.00]	3.00 [1.00, 4.00]	
t2_Trust_2				
Mean (SD)	2.62 (1.05)	3.22 (1.04)	3.03 (1.07)	0.0441
Median [Min, Max]	2.00 [1.00, 4.00]	4.00 [1.00, 4.00]	3.50 [1.00, 4.00]	

Note: p-value based on two-sample t-tests

3 Distribution of key demographics

Figure S1. Distribution of variables pre- and post-intervention (control group). Related to STAR Methods

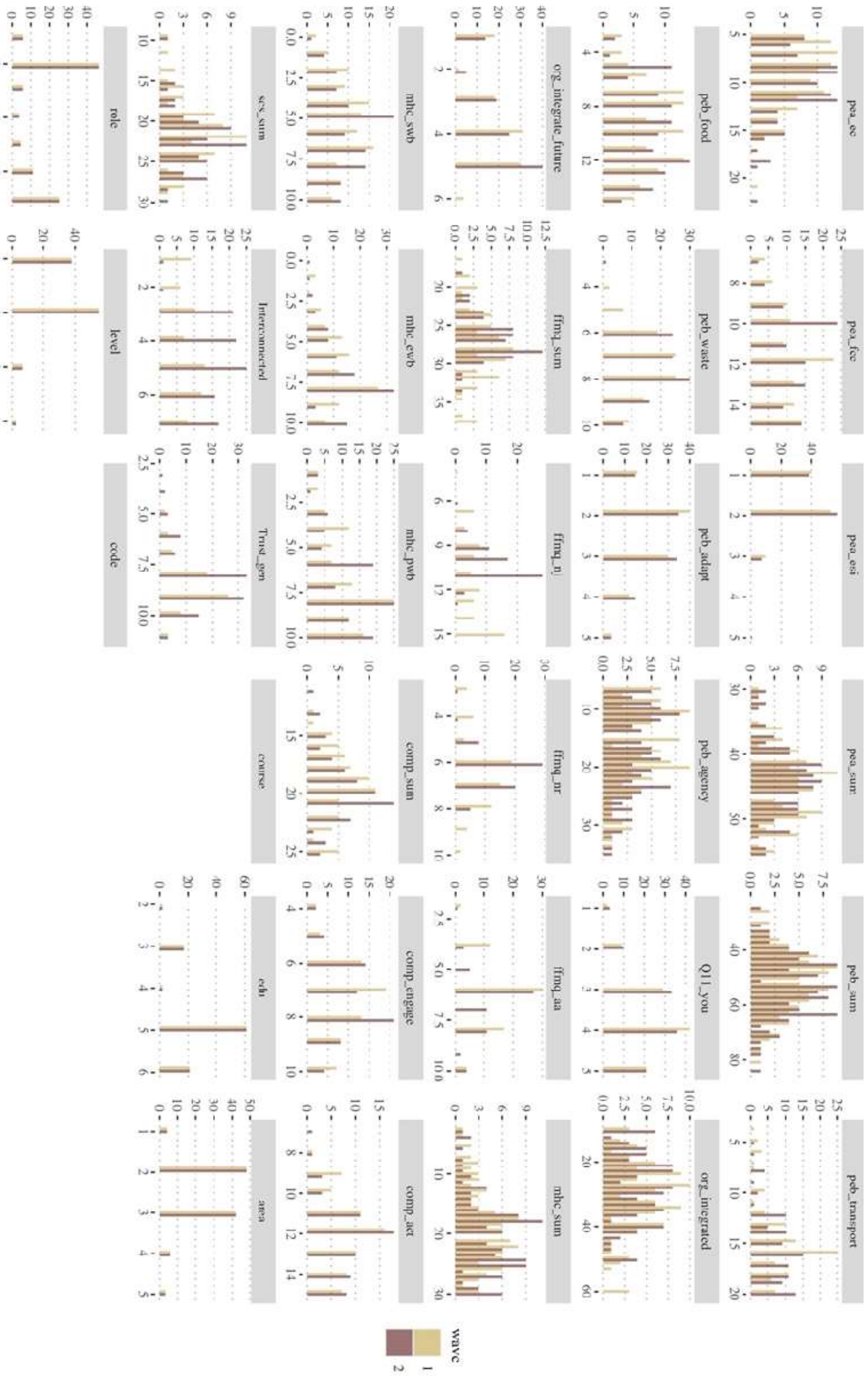

Figure S2. Distribution of variables pre- and post-intervention (intervention group). Related to STAR Methods

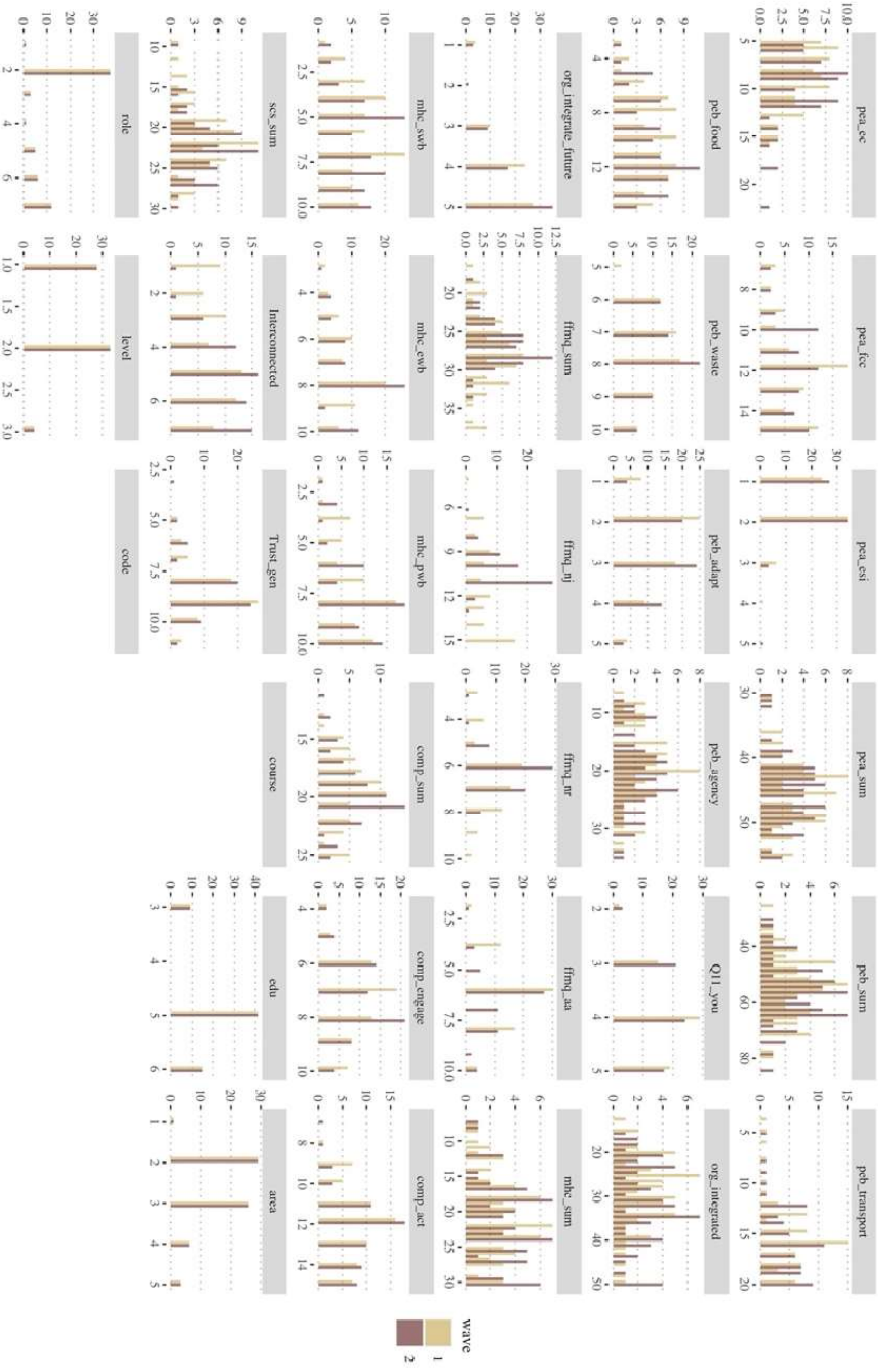

Figure S3. Distribution of Age. Related to STAR Methods

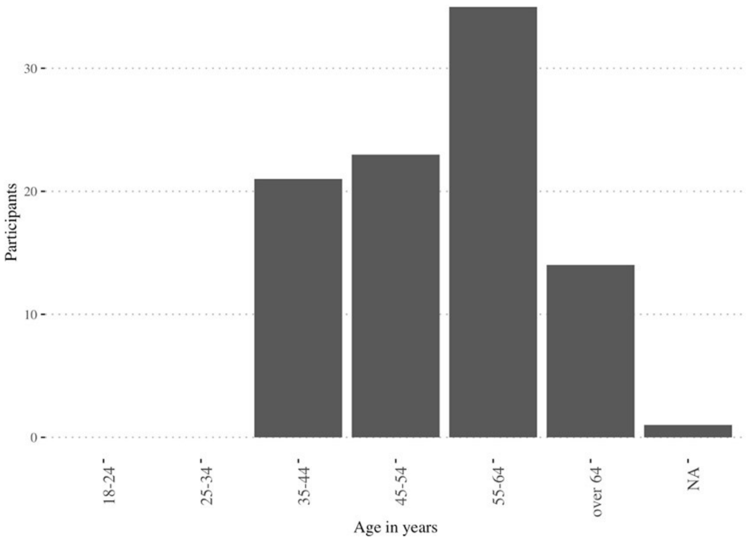

Figure S4. Distribution of Gender. Related to STAR Methods

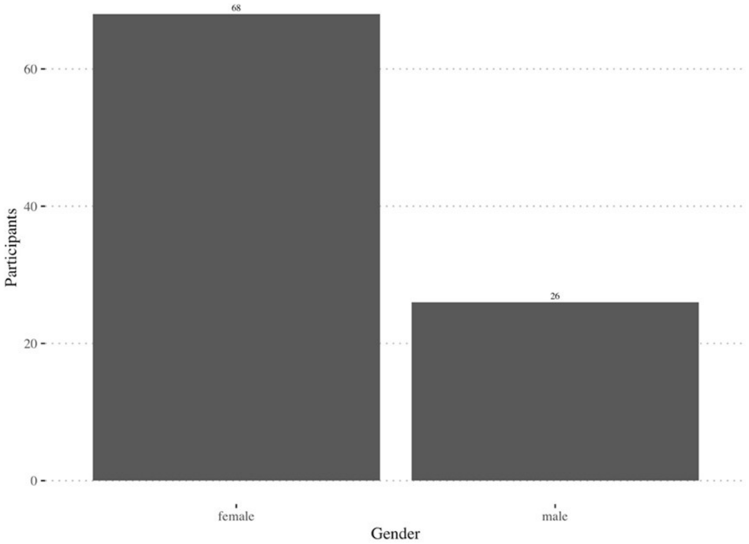

Figure S5. Distribution of Education. Related to STAR Methods

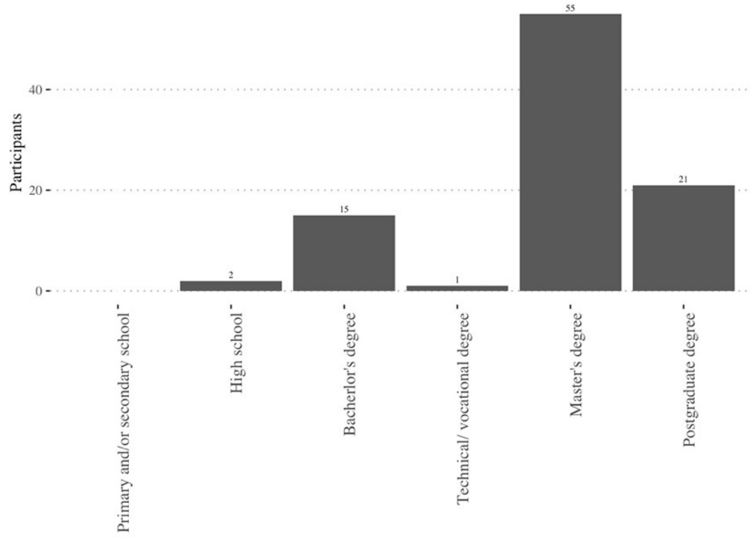

Figure S6. Distribution of Work Area. Related to STAR Methods

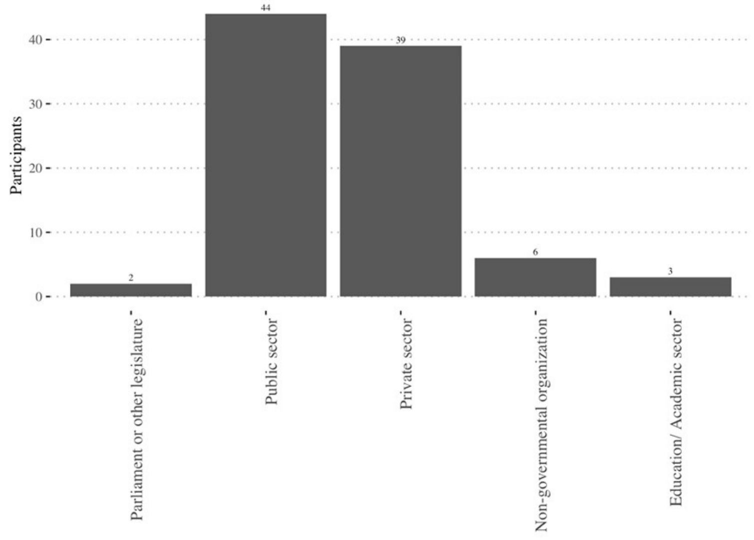

Figure S7. Distribution of Level of Work. Related to STAR Methods

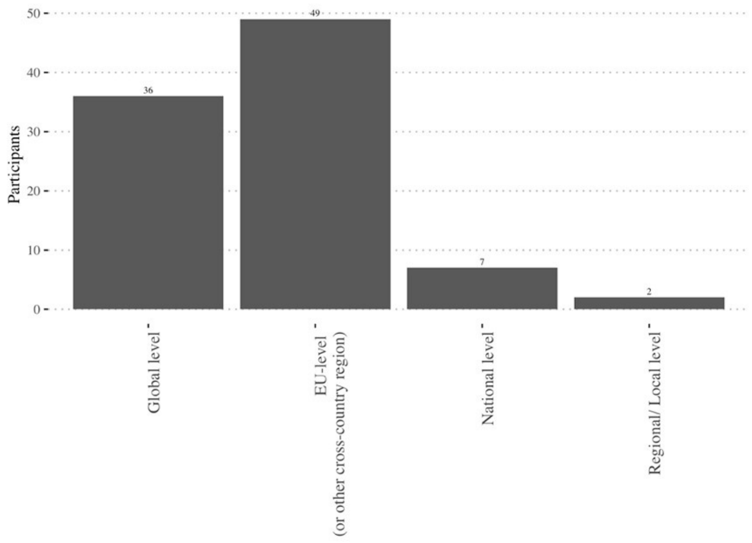

Figure S8. Distribution of Occupation/ Role. Related to STAR Methods

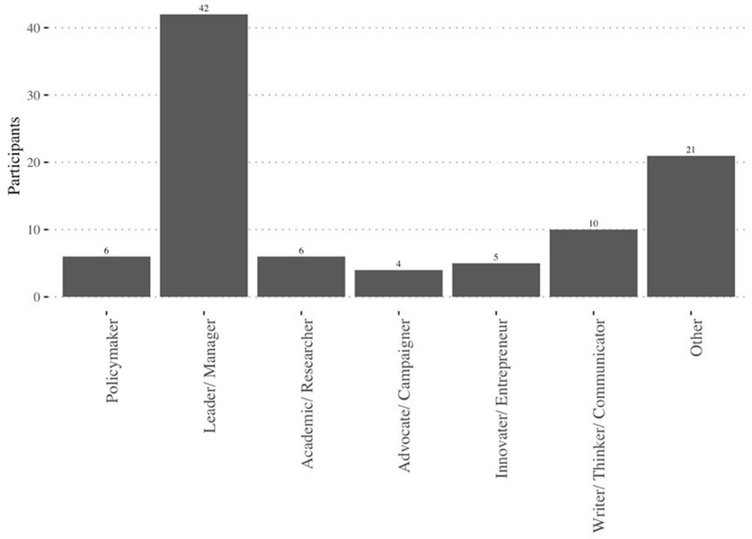

4 Theoretical background

Figure S9. Potential mechanisms of change. Related to STAR Methods and Section 2 of main manuscript

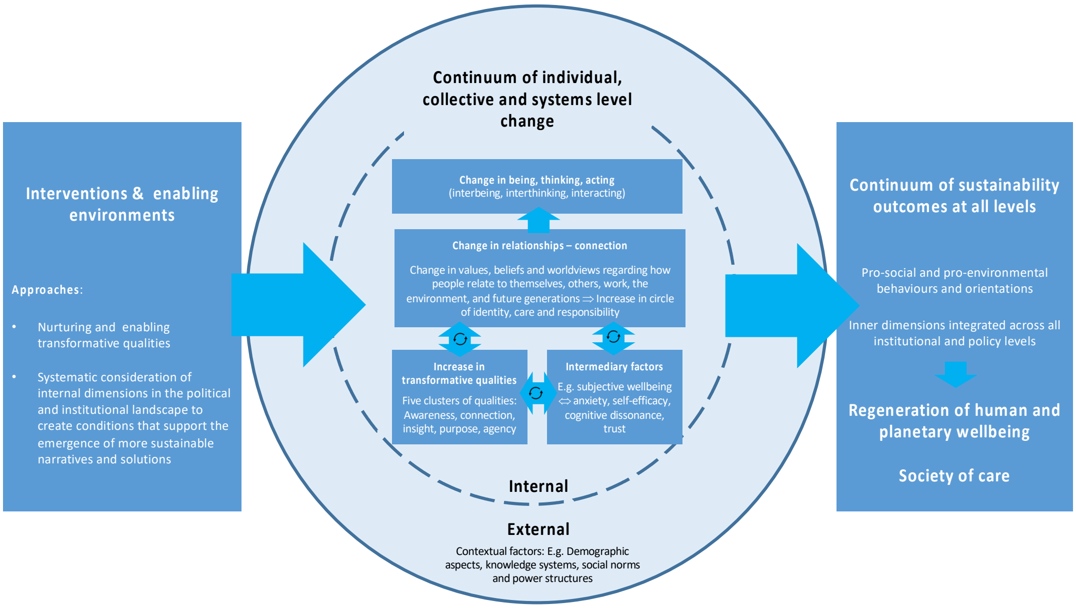

Source: https://www.sciencedirect.com/science/article/pii/S0959378021001527?via%3Dihub

5 Recruitment

Data S2. Recruitment procedure. Related to STAR Methods

We recruited a total of five distinct groups that underwent the training. Participants consisted of high-level decision-makers working in the field of sustainability and climate change, mainly from the European Commission, the European Parliament, other European, national or local policy institutions, and a multinational private company. The training company delivering these trainings (Awaris) had already worked within these organizations and established relationships with some human resources employees, which enabled them to advertise this new pilot training through their communication channels, such as the European Union's internal learning website, and internal sustainability/leadership networks, as well as social media platforms.

Advertisement

Courses were advertised on LinkedIn using texts similar to the following:

“We are launching our first series of online Climate Leadership programs for policy makers! You can join these FREE pilots if you send us a brief motivation. The pilots will be researched by […].
Don’t wait too long, half of the places are already taken!
FOR: policy-makers in and outside the EU Institutions. No formal leadership role required – just the willingness to lead!
DURATION: 7 online modules of around 2,5 hrs.
DATES: 7 Tuesdays or 7 Thursdays starting March 9 or 11.

Vulnerability Screening

After expressing interest in the programme, potential participants had to complete a pre-programme screening which served two purposes: First, to assess their commitment to the programme and ability to make space for home practices and survey completion. Participants were asked to reserve 20 to 30 minutes per day for self-paced practices and commit to completing the surveys and reflections. In addition, we wanted to identify participants suffering from mental health challenges. Participants that mentioned suffering from depression or another mental health challenge within the last 12 months were contacted by a trainer to make sure they are able actively take part in the course.

List of screening items:

- In order to achieve the programme's objective, we ask for substantial participant commitment. Do you feel able to reserve around 20 to 30 minutes per day for self-paced home practice (mindfulness or micro-habit practices, walks in nature, reflection, engagement on the learning platform)?
- This pilot program is in collaboration with our research partner […]. Participants will be asked to complete a pre-and post-survey (during workshop time) as well as short follow up surveys after each workshop module.
- In the last month, how often have you felt that you were unable to control the important things in your life?
- In the last 12 months, have you suffered from or been treated for a serious mental health challenge (e.g., depression, burnout, anxiety, addiction)?

6 Intervention details and comparison with other interventions

Table S3. Comparison of the Beyond Climate Leadership Training with Mindfulness-based Trainings. Related to STAR Methods

	Beyond Climate Leadership Training (intervention group)	Workplace Mindfulness Training (WorkingMind) (active control)	Typical MBIs (e.g. MBSR, MBCT)
Duration	10 weeks	10 weeks	8 weeks
Total Intervention hours		32	31
Target Groups	Leaders in Organisation and Governments seeking to act on climate change and/or sustainability	Individuals seeking to improve resilience and integrate mindfulness at work	Individuals seeking to improve stress management, symptom management
Focus	Individual wellbeing, acting on climate change, building a network to do that	Individual wellbeing and performance	Individual wellbeing
Number of Trainers	2	2	1
Daily required formal home practice	20-30 minutes	10 min or more	40 min or more
Guided Home Practice	Yes, app-based	Yes, app-based	Yes, various forms of delivery
Types of formal Practices	Body Awareness, Mindfulness, Compassion Practices, including Gratitude practice and climate specific adaptations/practices, e.g., observing emotions related to climate crisis, cultivating compassion for others	Body Awareness, Attention regulation Mindfulness, compassion practices	Body Awareness, Mindfulness, Compassion practices, yoga,
Types of informal practices	Connecting to nature, e.g., mindful nature walks	Mindfulness in work life, e.g., mindful meetings	Mindfulness in daily life, e.g., mindful cup of coffee
Psychoeducational Content	**Inner dimension:** Body-mind processes, emotion regulation, role of trust and psychological safety **Climate science:** Understanding of the latest climate science, system thinking, defining opportunities for actions that link	Focus and attention at work, mindfully working with time, positive outlook, communication, collaboration and trust, self-management and leadership	Automatic pilot, awareness of body and emotions, understanding and managing stress, mindful communication

	inner and outer dimensions		
Access to additional content	Yes, via a self-paced learning platform	Yes, via an app	No
Taking social actions	Yes, sustainability/ climate-related actions are defined in an action lab	No	No
Collaboration with others	Yes, a distinct goal is to build a network of change agents	No	No
Sustaining Mindfulness in an organisation	Developing internal champions	Developing internal champions	No

7 Sample overview

Table S4. Overview of Sample Structure. Related to STAR Methods

	**Intervention (N=65)**				**Control (N=29)**			
	EU Groups (D21EUC008) – 2 training groups	Lanxess Group (D21LAX010)	Mixed Group A (D21AWA054)	Mixed Group B (D21AWA066)	Lanxess (D21LAX083)	[…]Basecamp (D21-EUC-076)	People interested in sustainability (D21-CTR-001)	N total (intervention)
Number recruited (N)	46	19	20	21	20	74	20	220 (85)
Number of participants at t1 (N)	46	19	8	13	26	52	14	178 (73)
Number of participants at t2 (N)	28	18	12	15	12	23	6	(58)
Match t1/t2 (N)	29	17	6	13	11	14	4	94 (65)

7 Aggregated variables

Table S5. Overview of aggregated variables. Related to STAR Methods

Variable	Wording
Environmental attitudes	
Environmental concern	I would feel guilty if I did not participate in actions to protect the environment/ address climate change.
	I feel morally obliged to participate in actions to protect the environment/ address climate change.
	If we continue like this, we will head for a climate/ an environmental catastrophe.
	It makes me sad when I think about the loss of species and territories due to global warming.
Beliefs about fighting climate change	Modern science will solve our environmental problems with little change to our way of living. **[reverse]**
	In order to protect the environment the country needs economic growth. **[reverse]**
	We worry too much about the future of the environment and not enough about prices and jobs. **[reverse]**
Environmental Behavior	
Adapt	Take measures to be less affected/ more prepared for climate impacts.
Agency	Take part in a social movements/ protest about an environmental issue.
	Vote or support environmentally-oriented political candidates/ parties.
	Support environmental organizations/ groups (e.g. through donations or volunteering).
	Be an active member in an environmental organization.
	Actively spread awareness (e.g. through social media or other means).
	Create initiatives that addresses specific environmental needs/ challenges.
Transport	Reduce car transport
	Cut down on the amount I fly (e.g. no transatlantic and short-distance flights).
	Use public transportation whenever possible.
	Walk or cycle for non-recreational purposes.
	Live car-free.
	Use a battery electric car.
Food	Adopt a vegetarian diet.
	Adopt a vegan diet.
	Buy/ eat organic food.
	Reduce material consumption (e.g. reusing, less clothes/ electronics, minimalist lifestyle).
Waste	Minimize waste (e.g. food, packaging).
Mindfulness	
Mindfulness: Sum	sum score of all items
Mindfulness: Non-judgment	I think some of my emotions are bad or inappropriate and I shouldn't feel them.[reversed]
	I tell myself I shouldn't be feeling the way I'm feeling.[reversed]
Mindfulness: Non-reacting	When I have distressing thoughts or images, I "step back" and am aware of the thought or image without getting taken over by it.
	When I have distressing thoughts or images, I am able just to notice them without reacting.

Mindfulness: Acting with awareness	I do jobs or tasks automatically without being aware of what I'm doing.[reversed]<br>I find myself doing things without paying attention.[reversed]<br>I notice how foods and drinks affect my thoughts, bodily sensations, and emotions.
Mindfulness: Observe	
Subjective well-being	
Subjective well-being: Sum	sum score of all items
Subjective well-being: Social well-being	that you had something important to contribute to society
	that you belonged to a community
Subjective well-being: Emotional well-being	happy with life
	satisfied with life
Subjective well-being: Psychological well-being	that you had warm and trusting relationships with others
	that your life has a sense of direction or meaning to it
Self-Compassion: Sum	
Compassion for others: Sum	sum score of all items
Compassion for others: Engage	I notice and am sensitive to distress in others when it arises.
	I am accepting, non-critical and non-judgmental of other peoples' distress.
Compassion for others: Act	I direct attention to what is likely to be helpful to others.
	I take the actions and do the things that will be helpful to others.<br>I express feelings of support, helpfulness and encouragement to others.

8 Correlational Analyses

Table S6. Correlation coefficients with means, standard deviations, and confidence intervals. Related to STAR Methods and Section 3 of main manuscript

Variable	M	SD	1	2	3	4	5	6	7	8	9	10	11	12	13	14	15	16	17	18
1. ffmq_sum_d	2.28	4.72																		
2. mhc_sum_d	1.01	4.02	.05 [-.20, .30]																	
3. comp_sum_d	0.20	2.71	.12 [-.13, .36]	.13 [-.12, .36]																
4. scs_sum_d	0.94	3.05	.25 [-.00, .47]	.38** [.14, .57]	.27* [.03, .49]															
5. cwn_d	1.02	2.05	-.09 [-.33, .16]	.07 [-.18, .31]	.17 [-.08, .40]	.12 [-.13, .35]														
6. pea_ec_d	-0.83	2.61	.04 [-.22, .28]	-.00 [-.21, .20]	.17 [-.09, .40]	-.14 [-.37, .12]	-.02 [-.26, .23]													
7. pea_fcc_d	-0.19	1.62	.07 [-.18, .32]	.07 [-.14, .27]	-.11 [-.35, .15]	.13 [-.13, .37]	.17 [-.09, .40]	-.04 [-.25, .17]												
8. pea_wtpp_d	0.04	0.76	.22	-.06	.08	.01	.10	.11	.04											

			[-.03, .44]	[-.27, .14]	[-.17, .32]	[-.24, .26]	[-.15, .34]	[-.10, .30]	[-.17, .24]								
9. pea_wtps_d	-0.18	0.83	.25*	.04	.18	-.04	-.05	.19	-.00	.15							
			[.00, .47]	[-.17, .24]	[-.07, .41]	[-.28, .21]	[-.29, .20]	[-.02, .38]	[-.21, .20]	[-.06, .34]							
10. pea_esi_d	0.00	0.55	-.07	-.04	.24	.12	-.06	-.03	-.11	.05	-.09						
			[-.31, .18]	[-.25, .16]	[-.01, .46]	[-.13, .35]	[-.30, .19]	[-.23, .18]	[-.31, .10]	[-.15, .25]	[-.29, .11]						
11. peb_sum_d	1.48	5.96	.16	-.07	.29*	.19	.09	.21	-.14	.37**	.07	.29**					
			[-.10, .40]	[-.28, .14]	[.03, .51]	[-.08, .42]	[-.17, .34]	[-.00, .40]	[-.34, .08]	[.17, .54]	[-.14, .27]	[.08, .47]					
12. peb_food_d	0.20	1.79	-.14	.07	.12	-.00	.01	.25*	-.05	.10	.18	.05	.38**				
			[-.38, .11]	[-.14, .27]	[-.13, .36]	[-.25, .25]	[-.24, .25]	[.05, .44]	[-.25, .16]	[-.11, .30]	[-.03, .37]	[-.16, .25]	[.19, .55]				
13. peb_transport_d	0.61	3.02	.01	-.06	.30*	.11	-.03	.12	-.10	.30**	-.13	.27*	.72**	.25*			
			[-.24, .26]	[-.27, .14]	[.06, .51]	[-.15, .35]	[-.28, .22]	[-.09, .32]	[-.30, .11]	[.10, .48]	[-.32, .08]	[.06, .45]	[.61, .81]	[.05, .44]			
14. peb_waste_d	0.16	1.30	-.17	.00	.06	-.12	.09	.01	-.15	.07	-.00	.17	.38**	-.00	.25*		
			[-.41, .08]	[-.21, .21]	[-.19, .31]	[-.36, .14]	[-.16, .33]	[-.20, .22]	[-.34, .07]	[-.13, .28]	[-.21, .21]	[-.03, .37]	[.18, .54]	[-.21, .21]	[.04, .43]		
15. peb_agency_d	0.49	3.42	.38**	-.08	.16	.22	.02	.15	-.04	.23*	.14	.13	.67**	-.03	.11	.05	
			[.15, .58]	[-.28, .13]	[-.10, .39]	[-.03, .44]	[-.23, .26]	[-.06, .34]	[-.24, .17]	[.03, .42]	[-.07, .33]	[-.08, .32]	[.53, .77]	[-.24, .18]	[-.10, .31]	[-.16, .25]	
16. peb_adapt_d	0.17	1.07	.19	-.07	.01	.27*	.13	-.15	.04	.15	.01	.06	.27*	-.12	.03	.04	.19

			[-.06, .42]	[-.27, .13]	[-.24, .26]	[.02, .48]	[-.12, .36]	[-.35, .05]	[-.17, .25]	[-.06, .34]	[-.19, .21]	[-.15, .26]	[.06, .45]	[-.32, .09]	[-.18, .23]	[-.17, .25]	[-.01, .38]			
17. oms_you_d	-0.10	0.75	.08	-.02	-.17	-.10	.17	-.00	.03	.08	-.13	-.05	.13	-.04	.12	.09	.13	-.03		
			[-.17, .32]	[-.22, .18]	[-.39, .08]	[-.34, .15]	[-.07, .40]	[-.21, .20]	[-.18, .23]	[-.12, .28]	[-.33, .07]	[-.25, .15]	[-.08, .33]	[-.25, .16]	[-.09, .32]	[-.11, .29]	[-.08, .32]	[-.24, .17]		
18. oms_integrated_d	-0.10	7.39	.18	-.09	-.02	.06	-.18	-.21*	-.06	-.08	-.07	.16	-.13	-.11	-.11	-.15	.02	-.03	.01	
			[-.08, .42]	[-.29, .13]	[-.28, .24]	[-.20, .32]	[-.41, .08]	[-.41, -.00]	[-.27, .15]	[-.29, .13]	[-.27, .15]	[-.05, .36]	[-.34, .08]	[-.31, .10]	[-.31, .11]	[-.36, .06]	[-.19, .23]	[-.24, .18]	[-.20, .22]	
19. oms_integrate_personal_d	0.21	1.13	-.01	.06	.11	.22	.01	-.09	-.08	-.01	-.02	.16	.12	-.09	.06	.23*	.04	.22*	.23*	.04
			[-.25, .24]	[-.14, .26]	[-.14, .34]	[-.03, .44]	[-.24, .25]	[-.29, .11]	[-.28, .13]	[-.21, .19]	[-.22, .19]	[-.05, .35]	[-.09, .32]	[-.29, .11]	[-.14, .26]	[.03, .42]	[-.17, .24]	[.02, .41]	[.03, .41]	[-.17, .25]

Note. *M* and *SD* are used to represent mean and standard deviation, respectively. Values in square brackets indicate the 95% confidence interval for each correlation. The confidence interval is a plausible range of population correlations that could have caused the sample correlation (Cumming, 2014). * indicates $p < .05$. ** indicates $p < .01$.

Figure S10. Correlation heatmap of change scores. Related to STAR Methods and Section 3 of main manuscript

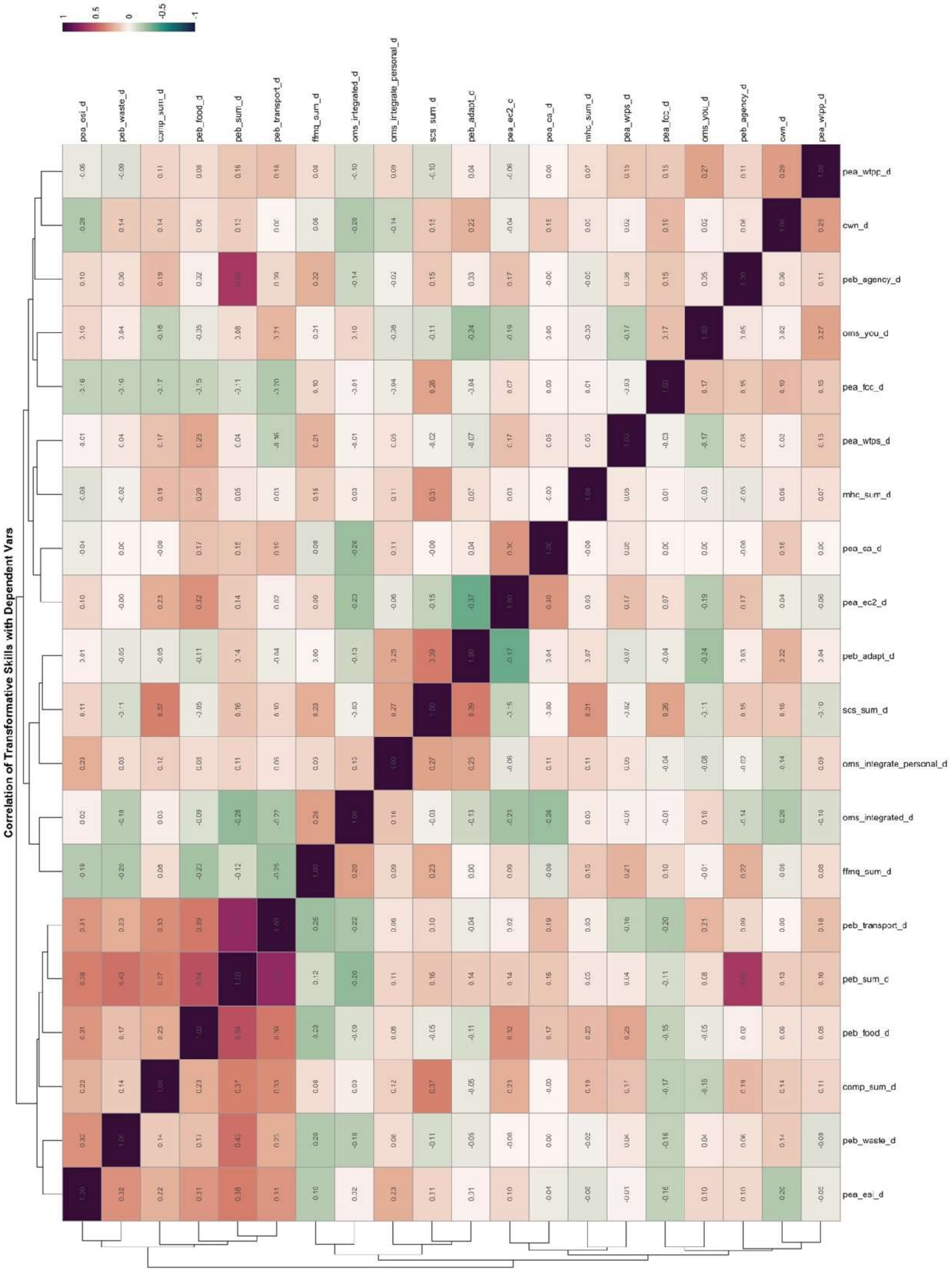

Supplement: Document S1. Figures S1–S10 and Tables S1–S6 [file mmc1.pdf]
